# Lipoprotein Lipase: Structure, Function, and Genetic Variation

**DOI:** 10.3390/genes16010055

**Published:** 2025-01-05

**Authors:** Shehan D. Perera, Jian Wang, Adam D. McIntyre, Robert A. Hegele

**Affiliations:** 1 Robarts Research Institute, Schulich School of Medicine and Dentistry, Western University, 4288A-1151 Richmond Street North, London, ON N6A 5B7, Canada; sperera8@uwo.ca (S.D.P.); jwang@robarts.ca (J.W.); amcintyre@robarts.ca (A.D.M.); 2 Department of Biochemistry, Schulich School of Medicine and Dentistry, Western University, 1151 Richmond Street North, London, ON N6A 5B7, Canada; 3 Department of Medicine, Schulich School of Medicine and Dentistry, Western University, 1151 Richmond Street North, London, ON N6A 5B7, Canada

**Keywords:** lipoprotein lipase, chylomicronemia, hypertriglyceridemia, complex trait, human genetics, genomic disorders

## Abstract

Biallelic rare pathogenic loss-of-function (LOF) variants in lipoprotein lipase (*LPL*) cause familial chylomicronemia syndrome (FCS). Heterozygosity for these same variants is associated with a highly variable plasma triglyceride (TG) phenotype ranging from normal to severe hypertriglyceridemia (HTG), with longitudinal variation in phenotype severity seen often in a given carrier. Here, we provide an updated overview of genetic variation in *LPL* in the context of HTG, with a focus on disease-causing and/or disease-associated variants. We provide a curated list of 300 disease-causing variants discovered in *LPL*, as well as an exon-by-exon breakdown of the *LPL* gene and protein, highlighting the impact of variants and the various functional residues of domains of the LPL protein. We also provide a curated list of variants of unknown or uncertain significance, many of which may be upgraded to pathogenic/likely pathogenic classification should an additional case and/or segregation data be reported. Finally, we also review the association between benign/likely benign variants in *LPL*, many of which are common polymorphisms, and the TG phenotype.

## 1. Introduction

Lipoprotein lipase (LPL) is the major enzyme responsible for regulating plasma triglyceride (TG) levels in many animal species, including humans [1]. Biallelic loss-of-function (LOF) variants in the *LPL* gene are the predominant cause of familial chylomicronemia syndrome (FCS) cases worldwide, accounting for ~80% of all cases [2]. This form of FCS is synonymous with LPL deficiency or the former Frederickson hyperlipoproteinemia type 1. FCS is characterized by severely compromised if not completely absent plasma lipolytic activity, leading to the pathogenic accumulation of predominantly intestinally derived chylomicrons (CMs), which produce refractory, severe hypertriglyceridemia (HTG; severe HTG is defined as fasting plasma TG ≥ 10 mmol/L), characteristic physical findings (such as eruptive xanthomas, lipemia retinalis, etc.), failure to thrive in infants, and most seriously, significantly increased lifetime risk for developing acute pancreatitis [2].

The phenotype associated with monoallelic LOF variants in *LPL* is less appreciated and familiar. We previously found that patients possessing a single affected *LPL* copy present with highly variable fasting plasma TG levels, both within and between patients longitudinally, ranging from normal TG levels to severe HTG, with secondary factors likely playing a major role in modulating the TG phenotype expressed by carriers of monoallelic LOF variants in the *LPL* gene [3]. Prior to our report, this variability was not widely known or reported, and our results may have seemed counterintuitive to previously held assumptions about the TG phenotype associated with heterozygosity for LOF variants in *LPL*, the potential severity of which may have been underestimated.

Here, we synthesize this new understanding of the phenotype of monoallelic LOF variants in *LPL* with recent findings regarding LPL structure, isoforms, regulation, and functionality, alongside previously well-established findings, to provide an up-to-date characterization of the *LPL* variants as they relate to HTG.

## 2. Synthesis and Expression of LPL

LPL is predominantly synthesized by the parenchymal cells of adipose, skeletal muscle, and cardiac muscle tissues and ultimately expressed in the vascular lumen of capillaries supplying these tissues, bound to the surface of endothelial cells [4,5].

LPL expression is highly regulated and complex, with varying physiological states, such as fasting and fed states, invoking tissue-specific differential LPL expression and regulation patterns. The synthesis, secretion, and expression of mature LPL have been previously reviewed by Wu, Kersten and Qi in 2021 [6]. Upon translation of the signal peptide sequence, the nascent LPL peptide is targeted to and transported into the endoplasmic reticulum (ER) via the signal peptide.

Once in the ER and after cleavage of the signal peptide, the nascent LPL peptide is completely translated and then undergoes two major post-translational processes, N-linked glycosylation at two sites, Asn70 and Asn386 (note: amino acid numbering throughout this review includes the signal peptide residues as residues 1–27), and the formation of five intra-molecular disulfide bridges, Cys54-Cys67, Cys243-Cys266, Cys291-Cys302, Cys305-Cys310, and Cys445-Cys465. Glycosylation of Asn70 has been previously shown to be necessary for LPL activity and proper intracellular trafficking [7]. Similarly, the Cys243-Cys266, Cys291-Cys302, and Cys305-Cys310 disulfide bridges have also been shown to be essential for LPL activity [8].

Either as part of the process of facilitating these post-translational modifications or directly following them, the action of lipase maturation factor (LMF1), one of the four essential cofactors of LPL in which biallelic LOF variants have also been found to cause FCS [2], and Sel-1 suppressor of Lin-12-Like 1 (SEL1L), both found on the ER membrane, ensure proper folding and maturation of the nascent LPL peptide. However, the exact roles of both LMF1 and SEL1L in the LPL maturation process are not well defined and remain somewhat unclear.

Some evidence indicates that LMF1 may help facilitate and promote proper formation of the five disulfide bridges in LPL by interacting with several other ER-resident chaperone proteins, such as Erp72, Erp44, ERdj5, and thioredoxin [9]. SEL1L is best known for its role as an obligatory co-factor for the E3 ligase HRD1 involved in ER-associated degradation (ERAD), a critical mechanism that specifically targets ER proteins for proteasomal degradation in the cytosol [10]. It has been suggested that SEL1L may facilitate and promote LPL maturation through both SEL1L-HRD1 ERAD-related and ERAD-independent pathways.

It has been observed in mice that high molecular weight aggregates of LPL peptide form within the ER in the absence of SEL1L. Given this, it has been speculated that nascent LPL peptide may be prone to misfolding, and SEL1L-HRD1 ERAD may play a critical role in clearing misfolded peptide from the ER to prevent aggregate formation and allow proper LPL maturation to take place [6]. Additionally, there is some in vitro evidence indicating that SEL1L physically interacts with both nascent LPL peptide and LMF1 and promotes their interaction within the ER, indicating that SEL1L has an additional role in LPL maturation, independent of the SEL1L-HRD1 ERAD mechanism [11]. Ultimately, after the action of LMF1 and SEL1L, LPL is secreted from the ER to the Golgi apparatus for packaging and transport to its secretory pathway.

Within the Golgi apparatus, LPL monomers are further modified with the addition of complex oligosaccharide groups [12]. LPL is then targeted to one of two different fates within the *trans*-Golgi network: (1) if the mature LPL binds to the heparan sulfate chain of Syndecan-1 (SDC-1), an integral membrane heparan sulfate proteoglycan (HSPG), it is preferentially sorted to exocytic vesicles of the sphingomyelin secretory pathway [13]; or (2) if mature LPL binds to sortilin-related receptor (SorLA-1), which has been observed to occur in neutral and acidic conditions within vesicular structures, LPL is targeted to a lysosomal degradation pathway [14]. It has been noted that LPL is still constitutively secreted even in the absence of SDC-1 [15], likely through the “bulk-flow” mechanism [16] thought to underlie constitutive secretion of many soluble secretory proteins, although, to the best of our knowledge, it currently remains unknown whether there are any secretion rate differences between the SDC-1-mediated secretion pathway and the “bulk-flow” mechanism for LPL [15].

Within secretory vesicles, LPL may form, in a concentration-dependent manner, catalytically inactive dihedral LPL dimers that assemble into helical oligomers, hypothesized to be a form of LPL storage prior to release and secretion of the catalytically active form in response to substrate/nutritional signaling [17]. In response to substrate availability/nutritional signaling, LPL is released and secreted into the interstitial space, where it binds to negatively charged HSPGs on the parenchymal cell surface via a positively charged region, where LPL eventually interacts with/is captured by glycosylphosphatidylinositol-anchored high-density lipoprotein binding protein 1 (GPIHBP1) (another one of the four essential cofactors of LPL in which biallelic LOF variants have also been found to cause FCS [2]) expressed on the basal surface of endothelial cells [18,19,20]. The newly formed LPL-GPIHBP1 complex transcytoses from the interstitial space to the capillary luminal surface of the endothelial cell [21], where LPL is anchored to catalyze the hydrolysis of circulating TGs from various lipoprotein species, primarily CMs and very-low-density lipoproteins (VLDLs) [1].

## 3. Physiological Roles of LPL in Lipoprotein Metabolism and Their Regulation

LPL has two primary functions related to plasma lipid metabolism: (1) catalysis of the rate-limiting step of plasma TG clearance, specifically of the hydrolysis of circulating plasma TGs, and (2) mediating tissue uptake of several circulating lipoprotein species via mechanisms independent of its catalytic activity.

The lipolytic function of what we now know to be LPL was first observed by Paul Hahn in 1943, when he found that injection of heparinized whole blood or plasma from donor dogs into lipemic dogs abolished their lipemia [22]. However, the specific heparin-induced clearing factor was not isolated and characterized as “lipoprotein lipase” until 1955 by Edward Korn [23,24]. Since then, a rich body of research has accumulated characterizing the biochemistry of LPL-mediated lipolysis.

The basic series of events that lead to LPL-mediated hydrolysis of TGs has been reviewed previously [25,26]. Briefly, the likely sequence of events begins when circulating TG-rich lipoproteins (TRLs), such as VLDLs and/or CMs, bind to endothelial cell membrane-bound LPL-GPIHBP1 unit(s), leading to a confirmational change in LPL that alters the lid region from a closed configuration that blocks substrate access to the active site to an open configuration [25,26,27,28]. This interaction is mediated by TRL-bound apolipoprotein (apo) C-II, an essential activator of LPL, binding to and stabilizing the lid region of LPL (discussed in more detail in the “Protein Structure of LPL” section of this review) [29,30] and TRL-bound apo A-V, another essential cofactor of LPL, binding to GPIHBP1 [31,32], which enhances the association of apo A-V containing lipoproteins with the endothelial cell surface features associated with LPL. We have previously reviewed apo A-V (*APOA5*) genetic variation and its roles in plasma lipid metabolism [33].

Either simultaneously or following this confirmational change upon TRL binding, the β5 loop of the N-terminal domain of LPL likely folds back, bringing the oxyanion hole into a catalytically competent position [25]. The sum of these conformational changes is the exposure of hydrophobic residues within and lining the active site, which attracts the FA chains of TG molecules to the catalytic triad and enables access of the glycerol backbone of TG molecules to the oxyanion hole, enabling hydrolysis of the TG molecule to two FFAs and one 2-monoacylglycerol molecule [25].

Interaction of hydrophobic residues of the lid region and the C-terminal lipid binding domain found on the same face of the 3-dimensional structure of LPL (discussed in more detail in the “Protein Structure of LPL” section of this review) with lipid substrate have been suggested as a potential mechanism by which LPL binds lipid substrate with the correct orientation to facilitate substrate access and entry to the active site [28].

As membrane-bound LPL hydrolyzes TGs to produce FFAs, their accumulation locally triggers the dissociation of LPL from GPIHBP1 [34], releasing the LPL monomer into the plasma where it has been recently proposed to undergo a tail-to-tail homodimerization interaction forming a circulating LPL homodimer [35] that binds to TRLs and their remnants, further hydrolyzing their TG content as they circulate [36], eventually being absorbed by the liver via the action of hepatic LDL receptor-related protein 1 (LRP1), which has been shown to interact with LPL homodimers to enhance hepatic uptake of LDL remnants [37,38].

Rather interestingly, the lipolytic activity of LPL has been associated with several additional physiological roles/effects. First, it has been shown that, at least partially through its enzymatic activity, LPL promotes the proliferation of vascular smooth muscle cells [39,40]. Second, it has been shown that increased LPL lipolytic activity is associated with reduced coronary heart disease and diabetes risk in humans [41,42], with increased insulin sensitization being observed in patients with mutations inactivating angiopoietin-like protein 3 (ANGPTL3), an inhibitor of LPL, and in ANGPTL3 knockout mice [43]. Third, LPL activity has been shown to be necessary for hematopoietic stem progenitor cell maintenance during definitive hematopoiesis in a zebrafish model by regulating and maintaining the LPL-mediated release of the essential fatty acid docosahexaenoic acid [44].

LPL catalytic activity and expression are regulated by two primary groups of proteins: (1) the angiopoietin-like (ANGPTL) protein family members ANGPTL3, ANGPTL4, and ANGPTL8 [4,6,45,46,47,48], and (2) several apolipoproteins, most significantly apo A-V, apo C-I, apo C-II, apo C-III, and apo E [4,6,29,45,49,50,51,52,53,54,55,56,57].

The ANGPTLs listed all inhibit LPL activity and are involved in the differential tissue expression of LPL in fed versus fasted states [45,58,59,60,61]. Specifically, increased insulin signaling in the fed state induces hepatic and adipose tissue ANGPTL8 expression, while downregulating hepatic apo A-V production and adipose tissue ANGPTL4 expression. This leads to an increase in the amount of circulating ANGPTL3/8 complex uninhibited by apo A-V, leading to the suppression of LPL activity in oxidative tissues. In turn, LPL-mediated TG hydrolysis is increased in adipose tissue as ANGPTL8 is expressed in adipose tissue complexes with remaining ANGPTL4, leading to formation of the ANGPTL4/8 complex, which has reduced LPL-inhibitory ability than ANGPTL4 alone. Additionally, ANGPTL4/8 complex binding to LPL in adipose tissue prevents the circulating ANGPTL4 and ANGPTL3/8 complex from binding them instead. The net effect of this is that most circulating plasma TG in the fed state is selectively hydrolyzed and taken up by adipose tissues for energy storage.

The opposite occurs in the fasted state, where increased apo A-V levels due to lack of insulin signaling enable apo A-V to block the inhibitory effects of the circulating ANGPTL3/8 complex and the reduced adipose tissue ANGPTL8 expression leads to increased ANGPTL4-mediated inhibition of adipose tissue LPL. With respect to the second group of regulators, the action of apo C-II and apo A-V has already been discussed (and apo C-II binding to LPL is further discussed in the “Protein Structure of LPL” section), but the action of apo C-I, apo C-III, and apo E has not been discussed.

**Figure 1:**
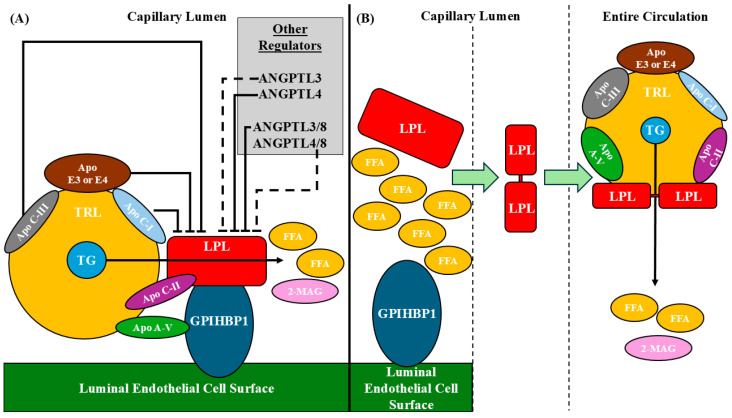
Summary of the catalytic functionality of lipoprotein lipase (LPL). (**A**) Monomeric LPL is bound to the luminal surface of endothelial cells by GPIHBP1 [1,21] in capillaries supplying skeletal muscle, cardiac muscle, or adipose tissues. Triglycerides (TGs) are transported throughout the body via TG-rich lipoproteins (TRLs), specifically chylomicrons and very-low-density lipoproteins, which carry TGs of dietary and hepatic origin, respectively. Apolipoproteins (apo) found on the surface of TRLs facilitate their interaction with membrane-bound LPL-GPIHBP1. TRL-bound apo A-V binds to the GPIHBP1 component, enhancing the association of the TRLs with LPL and the cell-surface features associated with LPL [31,32]. TRL-bound apo C-II interacts with multiple motifs in LPL itself, including the lid region of the LPL protein, inducing confirmational changes in LPL that enable the entry of TGs from the TRL into the LPL active site [29,30]. Membrane-bound LPL then hydrolyzes the TGs to 2 free fatty acid (FFA) molecules and one 2-monoacylglycerol molecule (2-MAG) [25]. The catalytic activity of LPL may be inhibited by other apolipoproteins on the surface of TRL, namely apo C-III, apo C-I, and/or possibly apo E (specifically, the E3 or E4 isoforms) [54,63]. Membrane-bound LPL activity may also be inhibited by angiopoietin-like proteins (ANGPTL) 3, 4, or 8 [4,6,45,46,47,48], but ANGPTL8 only inhibits LPL when bound to either ANGPTL3 or 4 [64,65]. Furthermore, the ANGPTL3/8 complex has an increased inhibitory effect compared to ANGPTL3 alone (represented in the figure with a dashed inhibition arrow for ANGPTL3 and a solid inhibition arrow for ANGPTL3/8) [65]. Conversely, the ANGPTL4/8 complex has a decreased inhibitory effect compared to ANGPTL4 alone (represented in the figure with a dashed inhibition arrow for ANGPTL4/8 and a solid inhibition arrow for ANGPTL4) [65]. (**B**) The accumulation of FFA locally due to the action of membrane-bound LPL triggers the dissociation of LPL and membrane-bound GPIHBP1 [34], releasing an LPL monomer into the circulation. It has been proposed that following this, LPL rapidly undergoes tail-to-tail dimerization, forming an LPL homodimer [35] that then binds to circulating TRL and TRL remnants (not shown) and continues to hydrolyze their remaining TG content [35,36]. Abbreviations: Angiopoietin-like protein, ANGPTL; Apolipoprotein, Apo; Free fatty acid, FFA; Glycosylphosphatidylinositol anchored high-density lipoprotein binding protein 1, GPIHBP1; Lipoprotein Lipase, LPL; 2-monoacylglycerol, 2-MAG; Triglyceride, TG; and TG-rich lipoprotein, TRL.

While the exact molecular mechanism is unknown, both apo C-I and apo C-III, which are bound to circulating lipoproteins, act as inhibitors of LPL activity, with some evidence suggesting that they do so by preventing the binding of LPL to the lipid/water interface of TRLs [54]. Interestingly, it has been observed that apo C-III exerts a greater inhibitory effect on GPIHBP1-bound LPL than on free LPL [62]. This is unexpected given that GPIHBP1 typically protects LPL from such effects by stabilizing it, as noted by the authors of this report on apo C-III [62]. Finally, the role of apo E in regulating LPL activity is isoform-dependent, with apo E3 and apo E4 being found to limit LPL-mediated TG hydrolysis [63]. Figure 1 summarizes the core elements of the catalytic functionality of LPL.

Independent of its lipolytic functionality, LPL has been observed to be involved in the tissue uptake of lipoproteins and their remnants by interacting with various cell surface features. Specifically, LPL has been found to (1) mediate the cellular uptake of LDL particles by interacting with both the LDL receptor (LDLR) and proteoglycans on the endothelial cell surface [66], (2) mediate hepatocyte selective uptake of high-density lipoprotein (HDL)-associated cholesteryl esters in a manner dependent on hepatocyte cell surface heparan sulfate proteoglycans but independent of LDLR- and LRP1-mediated endocytosis pathways [67], and (3) mediate TRL and TRL-remnant uptake in hepatocytes by interacting with LRP1 [68,69,70].

Finally, there is emerging evidence that LPL has important physiological roles in the central nervous system; while these are beyond the scope of this review, they are discussed at length elsewhere [71,72].

## 4. Protein Structure of LPL

To this day, the molecular structure of LPL in isolation has not been definitively characterized, due primarily to the fact that the catalytic hydrolase domain of LPL is inherently unstable and prone to spontaneous unfolding that corresponds to loss of catalytic functionality [73]. However, the finding that binding of the intrinsically disordered acidic domain of GPIHBP1 to LPL stabilizes the catalytic domain of LPL [73] has enabled two teams to characterize the crystal structure of the LPL-GPIHBP1 complex instead of LPL in isolation, allowing for new insights into the functional unit of plasma TG metabolism [27,28]. Prior to this, much of our knowledge of the LPL structure was derived from inference, primarily based on homology between LPL and pancreatic lipase, a related protein in the same lipase protein superfamily [74,75].

Following the cleavage of the 27 amino acid signal peptide, mature LPL is composed of 448 amino acids with a molecular mass of ~55 kDa [76]. Both crystallography studies characterizing the LPL-GPIHBP1 crystal structure confirmed that LPL, as predicted by homology with pancreatic lipase [74], has two primary functional domains: (1) an N-terminal α/β-hydrolase domain containing a catalytic triad and (2) a C-terminal lipid-binding domain formed by a β-barrel, which are connected by a hinge region [27,28].

The N-terminal α/β-hydrolase domain spans residues 28–340 [28] and includes several notable functional domains and residues. Ser159, Asp183, and His268 form a serine protease-like catalytic triad that is responsible for the catalytic functionality of LPL [27,28], with Trp82 and Leu160 forming an oxyanion hole that the glycerol backbone of TGs is thought to interact with for hydrolysis [25,28,77]. Related to these active site features are a number of hydrophobic residues whose side chains were found to line the active site cleft in the 3-dimensional structure of the LPL-GPIHBP1 complex, namely Trp82, Val84, Trp113, Tyr121, Tyr158, Leu160, Ala185, Pro187, Phe212, Ile221, Phe239, Val260, Val264, and Lys265, and have been stated to be involved in van der Waals interactions that likely guide and/or stabilize the hydrophobic tails of lipid substrate (i.e., TG) in the active site [27]. It should be noted that two of these residues identified by Birrane and colleagues in their study are in fact the residues forming the oxyanion hole described above (Trp82 and Leu160).

Also related to the active site is the lid region, which is thought to regulate substrate availability and specificity to the active site by adopting open or closed confirmations [27,28]. The lid region is formed by a loop of residues extending from the Cys243-Cys266 (with at least one review specifically identifying residues 245–265 as the lid region [6]) disulfide bridge in mature LPL [27,28]. A number of the lid region residues are also included in the above-discussed list of hydrophobic residues lining the active site cleft outlined by Birrane and colleagues in their study [27]. As noted by Arora and colleagues, in the open confirmation, several lid region hydrophobic residues (Ile245, Ile249, Val251, Ile252, Leu257, Val-260, Leu263, and Val264) in one of the structures they analyzed created a hydrophobic patch on the surface of the 3D structure of LPL, which was on the same face as a second hydrophobic patch formed by several residues (Tyr414, Phe415, Trp417, Trp-420, and Trp421) in the C-terminal Trp-rich lipid-binding domain [28]. This was posited by Arora and colleagues as a potential mechanism by which LPL binds to TRL substrates with the correct orientation to facilitate the entry of the substrate to the active site [28].

Another key structural component of LPL found within the N-terminal domain are five residues (Ala194, Arg197, Ser199, Asp201, and Asp202) that coordinate a calcium (Ca^2+^) ion necessary for proper folding of the mature LPL peptide [27]. Recent work has identified that the essential cofactor of LPL, apo C-II, likely exerts a significant part of its enhancing effect on LPL activity by augmenting the thermal stability of LPL (i.e., stabilizing the architecture of the catalytic pocket) and by interacting with and stabilizing the peptide sequences anchoring the lid region of LPL, most likely enabling efficient substrate entry into the catalytic pocket by keeping the lid in open confirmation [30].

Apo C-II was found to interact with four regions within the N-terminal domain of LPL, specifically residues 77–87 (corresponding to the segment connecting β-strand 2 and α-helix 2), 114–126 (corresponding to the segment connecting β-strand 3 and α-helix 3), 212–244 (corresponding to β-strand 7 and the segment connecting it to the lid region), and 245–263 (residues of the lid region) [30]. Interestingly, Kumari and colleagues observed that, with the exception of residues 212–244, these regions correspond with previously identified binding sites for ANGPTL4, a well-known inhibitor of LPL [30,78]. The binding of ANGPTL4 to these regions of LPL has been observed to have an opposite effect to the binding of apo C-II to them, leading to allosteric changes in LPL that destabilize and ultimately lead to the irreversible unfolding of the N-terminal α/β hydrolase domain [30,78].

As noted in the above section on the synthesis and expression of LPL, one of the two N-linked glycosylation sites is found within the N-terminal domain, namely Asn70, with the other being located within the C-terminal domain at Asn386 [27,28]. Finally, four of the five disulfide bridges (Cys54-Cys67, Cys243-Cys266, Cys291-Cys302, and Cys305-Cys310) are formed by residues within the N-terminal domain, which were also previously described in the “Synthesis and Expression of LPL” section [27,28].

The smaller C-terminal domain has two major sets of functional domains and/or residues: (1) a Trp-rich lipid-binding domain and (2) residues involved in interactions with and/or binding to GPIHBP1 [27,28]. The Trp-rich lipid-binding domain is contained within residues 412–422 [27,28,79]. This domain is responsible for substrate recognition and has been posited (as discussed above) to be involved in guiding the substrate to the active site in concert with hydrophobic side chains of lid region residues [28]. Finally, many residues in the C-terminal domain of LPL have been found to interact with GPIHBP1. GPIHBP1 interacts with the C-terminal domain of LPL via a Ly6/uPAR (LU) domain with a three-fingered fold conformation stabilized by five disulfide bonds in GPIHBP1 [27,80,81,82]. LPL residues 443–447 and 465–466 interact with finger 1, residues 447–448 and 463–467 interact with finger 2, and residues 367, 369, 374, 403–406, 447, and 464 interact with finger 3 [27].

Additionally, numerous other residues in the C-terminal domain of LPL are involved in hydrophobic interactions stabilizing the LPL-GPIHBP1 complex [27]. There are also two specific LPL residues found to be involved in hydrogen bonding between LPL and GPIHBP1, Arg447, and Glu384 [27]. GPIHBP1 also binds to an “interdomain interface” formed by LPL residues 306–317 via its intrinsically disordered acidic domain, which is an interaction that seems to be responsible for the ability of GPIHBP1 to stabilize and mitigate the spontaneous unfolding of the N-terminal α/β-hydrolase domain [73]. As noted previously, the second N-linked glycosylation site (Asn386) and the final disulfide bridge-forming residues (Cys445-Cys465) are also found within the C-terminal domain of LPL.

With respect to structural isoforms of LPL, historically it was thought that LPL monomers were catalytically inactive/non-functional and head-to-tail homodimerization, previously thought to be facilitated by the action of LMF1 in the ER, was required to produce a catalytically active LPL unit [76,83,84,85]. This head-to-tail homodimer structure was also observed in both recent crystallographic studies of the LPL-GPIHBP1 complex [27,28].

However, as discussed at length in both reports of the crystal structure of the LPL-GPIHBP1 complex, logically, the head-to-tail homodimer configuration produced by the intercalation of the Trp-rich lipid-binding domain of one LPL monomer with the active site of the opposite monomer would prevent substrate binding and catalytic activity [27,28]. Indeed, Arora and colleagues determined that the head-to-tail homodimer structure they observed and studied was likely not physiologically relevant, as it was an artifact of the crystal packing interaction utilized in their study [28]. This is consistent with recent observations of catalytically active LPL, both alone and complexed to GPIHBP1, challenging the historical view of the head-to-tail homodimer as the sole functional unit [76].

However, the picture has been further complicated by the recent discovery of two new LPL isoforms, including a new homodimer scheme. First, as previously discussed in the “Synthesis and Expression of LPL” section, it was found that LPL may form catalytically inactive helical polymers within secretory vesicles prior to their secretion, likely as a form of storage, indicating a new physiologically relevant non-active form of the protein [17]. Then, even more recently, a new LPL homodimer scheme was discovered.

Specifically, it was found in an in vitro model that the C-terminals of LPL monomers can interface (i.e., a tail-to-tail homodimer) to produce a catalytically active LPL unit [35]. Given the tail-to-tail interaction, it should be noted that this homodimer does not appear to be bound to the endothelial cell surface and instead likely circulates through the bloodstream, further hydrolyzing TGs in TRLs and their remnants as it does so [35]. In fact, this finding, while in vitro, is consistent with previous findings of dimeric LPL bound to circulating TRLs that continue to hydrolyze them as they circulate [36,86]. Analysis of the structure of this novel LPL homodimer revealed a hydrophobic pore adjacent to the active site, which was demonstrated to be able to accommodate TG fatty acid chains [35]. It was hypothesized that this hydrophobic pore was responsible for substrate specificity and may also be responsible for facilitating a unidirectional release of FFA from TG hydrolysis, which is rather interesting considering that the membrane-bound LPL-GPIHBP1 complex releases FFA following TG hydrolysis in a bidirectional manner [35].

## 5. Genomic Structure of *LPL*

The *LPL* gene is located on the short arm of chromosome 8 (8p21.3) and comprises 10 exons and 9 introns spanning ~30 kb [25,87]. Exons 1–9 are all roughly similar in size (ranging from 105 to 276 bp in length), with exon 10 being considerably larger at 1950 bp in length, as it encodes the entirety of the 3′ UTR region [25,87]. Given the structural similarities between LPL and other members of the lipase superfamily of proteins, namely hepatic lipase and pancreatic lipase, it seems likely that they derive from a common ancestral gene [25]. As discussed in previous sections, *LPL* is primarily expressed in adipose tissue, skeletal muscle, and cardiac muscle tissues [4].

## 6. A Curated Assembly of *LPL* Variants

Here, we report a curated assembly of *LPL* variants that have been reported in the literature and/or in variant databases as being causative for and/or associated with HTG (Supplementary Tables S1 and S2). We have subdivided this assembly into two tables based on variant pathogenicity determined according to the American College of Medical Genetics and Genomic (ACMG) guidelines [88], with Pathogenic/Likely Pathogenic variants in Supplementary Table S1 and variants of uncertain significance (VUS) in Supplementary Table S2. Supplementary Table S2 additionally contains several variants that may be upgraded from VUS to Likely Pathogenic upon the reporting of new case and/or segregation data and are listed as VUS* to indicate them. Except where the molecular defect is obvious (i.e., premature truncation variants, loss of a large number of nucleotides, etc.), detailed notes are provided regarding the evidence and molecular defect(s) associated with each variant (Supplementary Tables S1 and S2). Simple notes are provided where the associated defects are obvious. A brief overview and summary of these data are provided in Table 1, Table 2 and Table 3.

Additionally, we also compiled a non-comprehensive list of common polymorphisms in *LPL* that have been associated with plasma TG levels (Supplementary Table S3). This list is non-comprehensive as the focus of this review is rare disease-causing variants, not common polymorphisms. We also provided notes regarding the evidence for any molecular effect associated with these variants (Supplementary Table S3).

This curated list of *LPL* variants was obtained via a methodology similar to that used in our previous review of variants in *APOA5* in HTG [33]. Briefly, we began by compiling *LPL* variants listed as disease-causing or associated variants in the Human Gene Mutation Database (HGMD) [89], ClinVar [90], and the Leiden Open Variation Database 3.0 (LOVD3) [91] and double-checking the reporting of these variants in the literature where available. We then independently assessed the pathogenicity of these variants using our laboratory pipeline as we have previously described [3,92,93], utilizing the Franklin by Genoox tool (https://franklin.genoox.com) followed by manual curation to determine the pathogenicity classifications for all variants according to the ACMG guidelines [88]. We also included variants found in our own clinical testing at the Lipid Genetics Clinic, London, Ontario, Canada, if they were considered pathogenic or likely pathogenic under the ACMG guidelines and had not been previously reported in the literature.

We also included several variants from ClinVar and LOVD3 without literature references if they were reported as pathogenic/likely pathogenic in those sources prior to manual curation. Upon manual curation, a number of these variants were downgraded to VUS or VUS* (Supplementary Table S2). In total, we report a total of 75 variants in our curated assembly without literature references (Supplementary Tables S1 and S2) from our clinic, ClinVar, and LOVD3. We feel that inclusion of these variants is warranted given that they either (a) produce obvious molecular defects (i.e., premature truncation, splice site disruption, etc.), (b) have multiple forms of indirect evidence supporting a pathogenic effect (i.e., impacting a known or purported functional domain, multiple other variants reported as pathogenic impacting the same residue, etc.), or (c) have been observed/reported in clinical testing results as the cause of HTG.

We excluded variants reported in these sources from our curated assembly based on one or more of the following criteria: (a) large-scale variants extending to genes other than *LPL*, (b) synonymous variants with no evidence indicating association with TG phenotype, and (c) *LPL* variants reported in association with non-TG-related diseases such as neurological and/or developmental disorders. Additionally, we excluded 180 variants reported in HGMD from analysis and review, as at the time of writing, the only literature reference for these variants is a brief report from Deshotels and colleagues regarding the association of these variants and a polygenic risk score constructed utilizing (in part) these variants with HTG in academic lipid clinics [94]. The case data for the carriers of these 180 variants are unclear and/or unavailable, and to the best of our knowledge, they have only been reported in this report by Deshotels and colleagues. Thus, even if some of the variants reported by Deshotels and colleagues are likely deleterious, we felt that there is insufficient evidence to warrant including them for detailed analysis in our review, so they have been excluded from analysis and review. We include them in Supplementary Table S4 for readers’ reference.

In total, we compiled a total of 358 unique variants in *LPL* reported in the literature and/or in variant databases as causative for and/or associated with HTG and related phenotypes (Supplementary Tables S1 and S2). Specifically, we observed 300 pathogenic/likely pathogenic variants (Supplementary Table S1) and 58 VUS/VUS* variants (Supplementary Table S2).

**Figure 2:**
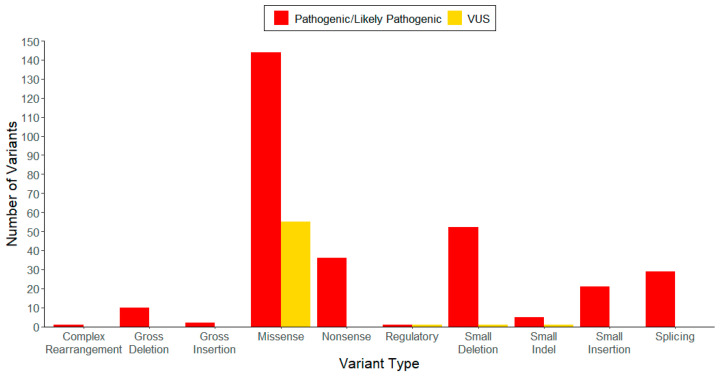
Distribution of *LPL* gene pathogenic/likely pathogenic variants and variants of uncertain significance (VUS) by variant type. The grouped bar chart shows the number of each variant type we identified as pathogenic/likely pathogenic (red bars) or VUS (gold bars) according to the American College of Medical Genetics and Genomics (ACMG) guidelines. The X-axis indicates the variant type and the Y-axis indicates the number of variants identified in our compiled lists (See Supplementary Data for more details).

Of the 300 pathogenic/likely pathogenic variants we compiled, 144 are missense variants, 36 are nonsense variants, 52 are small deletions, 21 are small insertions, five are small indels, 29 are splicing variants, 10 are gross deletions, two are gross insertions, one is a complex rearrangement, and one is a regulatory region variant (Table 1, Table 2 and Table 3). Of the 58 VUS variants, 55 are missense variants, one is a small deletion, one is a small indel, and one is a regulatory region variant (Table 1 and Table 2). These data are also displayed in Figure 2.

Several mechanisms of disease have been reported with respect to pathogenic variants in *LPL*. Some nonsense variants have been hypothesized to trigger nonsense-mediated mRNA decay [95,96,97,98,99]. The principal mechanism of disease, however, appears to be the disruption of functionally and/or structurally important domains and/or residues of the LPL peptide, particularly domains and residues involved in the catalytic functionality of the protein. Additionally, catalytically active LPL can have its secretion prevented by variants enhancing the susceptibility of the mature LPL peptide to endoproteolytic cleavage, leading to the loss of the C-terminal lipid-binding domain [100]. Finally, it has also been shown that it is possible for the immune system to produce antibodies against LPL, leading to a loss of their functionality [101]. More details regarding all these mechanisms and their related variants are presented in the following sections.

**Figure 3:**
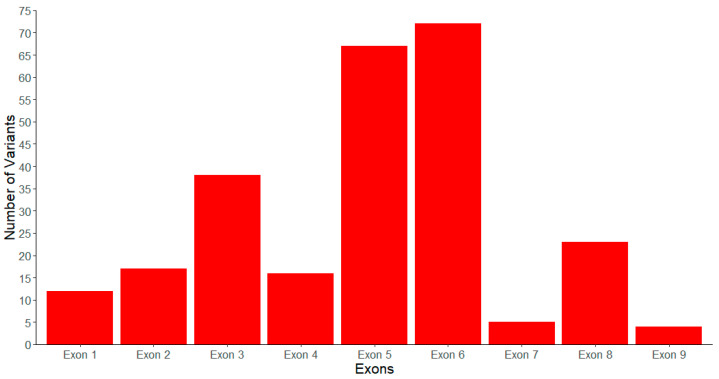
Distribution of pathogenic/likely pathogenic variants across all coding exons of the *LPL* gene. The bar chart shows the number of pathogenic/likely pathogenic variants (as identified according to the ACMG guidelines) found in each coding exon of the *LPL* gene. The X-axis indicates the exons and the Y-axis indicates the number of variants identified in our compiled list (See Supplementary Data for more details).

This is demonstrated by the concentration of pathogenic/likely pathogenic variants observed in the exons encoding these regions compared to the rest of the *LPL* gene. Specifically, not including large-scale variants and variants in non-coding regions of the *LPL* gene (regulatory and splicing region variants), there are 254 pathogenic/likely pathogenic variants impacting the coding sequence of *LPL* (Table 1). The distribution of these pathogenic variants across the coding sequence is as follows and is also shown graphically in Figure 3: 12 (4.72%) are in exon 1, 17 (6.69%) are in exon 2, 38 (14.96%) are in exon 3, 16 (6.30%) are in exon 4, 67 (26.38%) are in exon 5, 72 (28.35%) are in exon 6, five (1.97%) are in exon 7, 23 (9.06%) are in exon 8, and the final four (1.57%) are in exon 9. The vast majority of these impact exons 2–6 (82.68%), which together encode the N-terminal α/β-hydrolase domain of mature LPL [28], with exons 5 and 6 accounting for a particularly large proportion of the coding region pathogenic/likely pathogenic variants observed at 54.72%.

The increased concentration of pathogenic/likely pathogenic variants specifically in exons 5 and 6 likely stems from the fact that these two exons encode a large number of functionally and structurally important residues in mature LPL, including but not limited to (1) the majority of the known residues forming the active site, including two of the catalytic triad residues (Asp183 and H268), (2) the entirety of the lid region (residues between Cys243 and Cys266), (3) all five calcium ion coordination residues (Ala194, Arg197, Ser199, Asp201, and Asp202), and (4) three disulfide bridge-forming residues known to be essential (Cys243–Cys266, Cys291–Cys302, and Cys305–Cys310) (see “Protein Structure of LPL” section for more details on these domains). Exons 2–4 also encode many residues that line the active site and are likely involved in several functions such as apo C-II binding and lipid substrate interactions, as previously discussed in the “Protein Structure of LPL” section.

**Figure 4:**
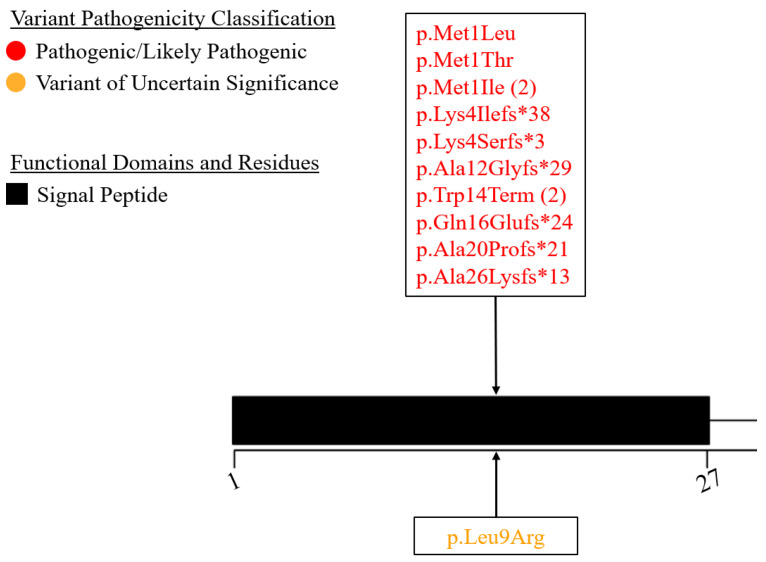
Protein map of reported *LPL* coding sequence variants encoded in exon 1. The number line indicates amino acid residue numbering in the primary structure of newly synthesized LPL peptide, with the specific numbers indicating the beginning and/or end of the functional domain they border. The black box indicates the residues forming the signal peptide. All variants are color-coded according to their ACMG pathogenicity classification: red indicates pathogenic or likely pathogenic and yellow orange indicates a variant of uncertain significance (VUS). Pathogenic/likely pathogenic variants are shown above the linear map, while VUS variants are shown below. For frameshift variants resulting in a premature stop codon, the notation ‘fs*(number)’ indicates that the frameshift variant results in stop codon at (number) of residues downstream of the variant site.

**Figure 5:**
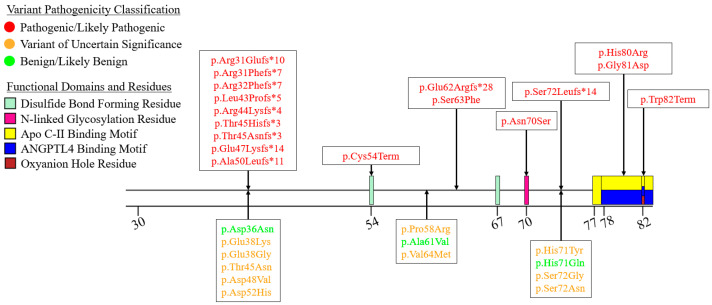
Protein map of reported *LPL* coding sequence variants encoded in exon 2. The number line indicates amino acid residue numbering in the primary structure of newly synthesized LPL peptide, with the specific numbers indicating the beginning and/or end of the functional domain they border. The light-green box indicates cysteine residues involved in the formation of intramolecular disulfide bonds. The pink box represents asparagine residues at which N-linked glycosylation has been found to occur. Yellow boxes represent residues involved in apo C-II binding. Blue boxes represent residues involved in ANGPTL4 binding. The brown box represents residues involved in the formation of an oxyanion hole in mature protein. Where residues have been found to be involved in multiple functions, multiple boxes are used to represent overlap. All variants are color-coded according to their ACMG pathogenicity classification: red indicates pathogenic or likely pathogenic, yellow orange indicates a variant of uncertain significance (VUS), and green indicates benign or likely benign. Pathogenic/likely pathogenic variants are shown above the linear map, while VUS and benign/likely benign variants are shown below. For frameshift variants resulting in a premature stop codon, the notation ‘fs*(number)’ indicates that the frameshift variant results in stop codon at (number) of residues downstream of the variant site.

**Figure 6:**
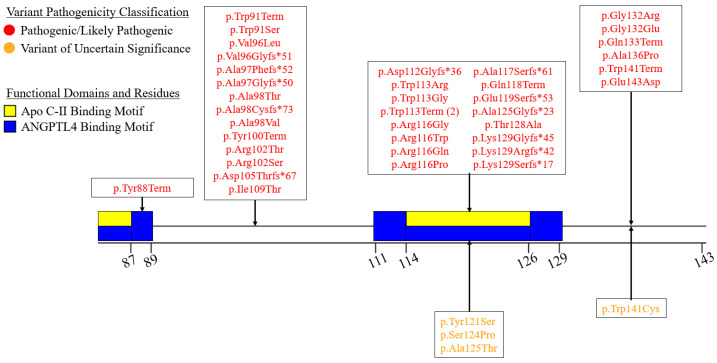
Protein map of reported *LPL* coding sequence variants encoded in exon 3. The number line indicates amino acid residue numbering in the primary structure of newly synthesized LPL peptide, with the specific numbers indicating the beginning and/or end of the functional domain they border. Yellow boxes represent residues involved in apo C-II binding. Blue boxes represent residues involved in ANGPTL4 binding. Where residues have been found to be involved in multiple functions, multiple boxes are used to represent overlap. All variants are color-coded according to their ACMG pathogenicity classification: red indicates pathogenic or likely pathogenic and yellow orange indicates a variant of uncertain significance (VUS). Pathogenic/likely pathogenic variants are shown above the linear map, while VUS variants are shown below. For frameshift variants resulting in a premature stop codon, the notation ‘fs*(number)’ indicates that the frameshift variant results in stop codon at (number) of residues downstream of the variant site.

**Figure 7:**
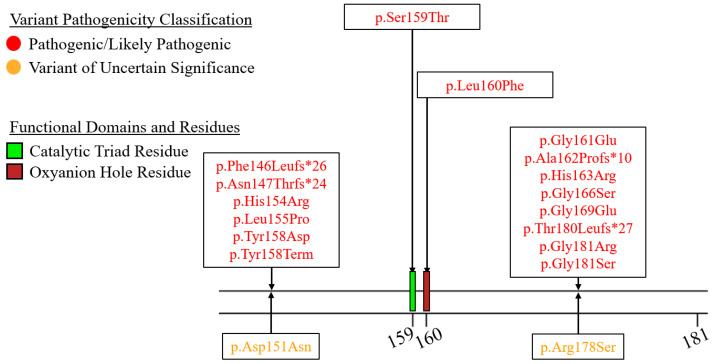
Protein map of reported *LPL* coding sequence variants encoded in exon 4. The number line indicates amino acid residue numbering in the primary structure of newly synthesized LPL peptide, with the specific numbers indicating the beginning and/or end of the functional domain they border. Green boxes represent residues forming the catalytic triad of the LPL hydrolase domain. The brown box represents residues involved in the formation of an oxyanion hole in mature protein. All variants are color-coded according to their ACMG pathogenicity classification: red indicates pathogenic or likely pathogenic and yellow orange indicates a variant of uncertain significance (VUS). Pathogenic/likely pathogenic variants are shown above the linear map, while VUS variants are shown below. For frameshift variants resulting in a premature stop codon, the notation ‘fs*(number)’ indicates that the frameshift variant results in stop codon at (number) of residues downstream of the variant site.

**Figure 8:**
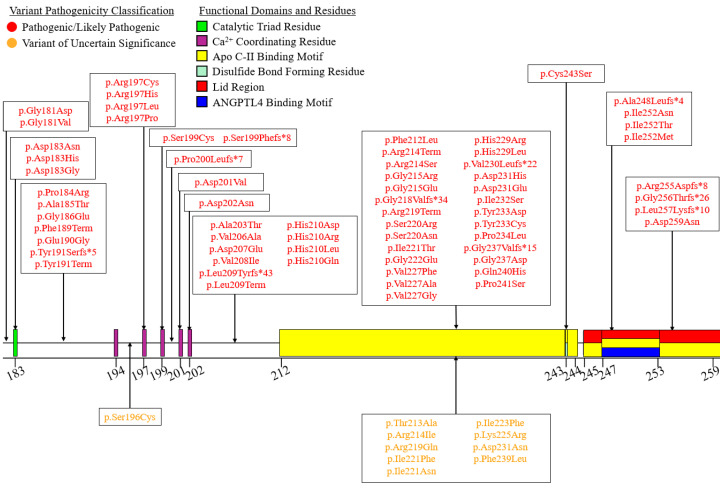
Protein map of reported *LPL* coding sequence variants encoded in exon 5. The number line indicates amino acid residue numbering in the primary structure of newly synthesized LPL peptide, with the specific numbers indicating the beginning and/or end of the functional domain they border. Green boxes represent residues forming the catalytic triad of the LPL hydrolase domain. Purple boxes represent residues involved in the coordination of Ca^2+^ into the mature LPL protein. Yellow boxes represent residues involved in apo C-II binding. The light-green box indicates cysteine residues involved in the formation of intramolecular disulfide bonds. Red boxes represent residues forming the lid region involved in regulating substrate access to the active site of mature LPL. Blue boxes represent residues involved in ANGPTL4 binding. Where residues have been found to be involved in multiple functions, multiple boxes are used to represent overlap. All variants are color-coded according to their ACMG pathogenicity classification: red indicates pathogenic or likely pathogenic and yellow orange indicates a variant of uncertain significance (VUS). Pathogenic/likely pathogenic variants are shown above the linear map, while VUS variants are shown below. For frameshift variants resulting in a premature stop codon, the notation ‘fs*(number)’ indicates that the frameshift variant results in stop codon at (number) of residues downstream of the variant site.

**Figure 9:**
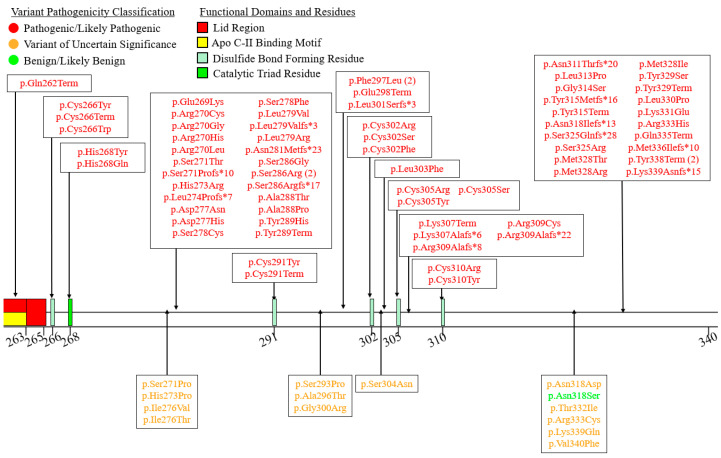
Protein map of reported *LPL* coding sequence variants encoded in exon 6. The number line indicates amino acid residue numbering in the primary structure of newly synthesized LPL peptide, with the specific numbers indicating the beginning and/or end of the functional domain they border. Red boxes represent residues forming the lid region involved in regulating substrate access to the active site of mature LPL. Yellow boxes represent residues involved in apo C-II binding. The light-green boxes indicate cysteine residues involved in the formation of intramolecular disulfide bonds. Green boxes represent residues forming the catalytic triad of the LPL hydrolase domain. All variants are color-coded according to their ACMG pathogenicity classification: red indicates pathogenic or likely pathogenic, yellow orange indicates a variant of uncertain significance (VUS), and green indicates benign or likely benign. Pathogenic/likely pathogenic variants are shown above the linear map, while VUS and benign/likely benign variants are shown below. Pathogenic/likely pathogenic variants are shown above the linear map, while VUS and benign/likely benign variants are shown below. For frameshift variants resulting in a premature stop codon, the notation ‘fs*(number)’ indicates that the frameshift variant results in stop codon at (number) of residues downstream of the variant site.

**Figure 10:**
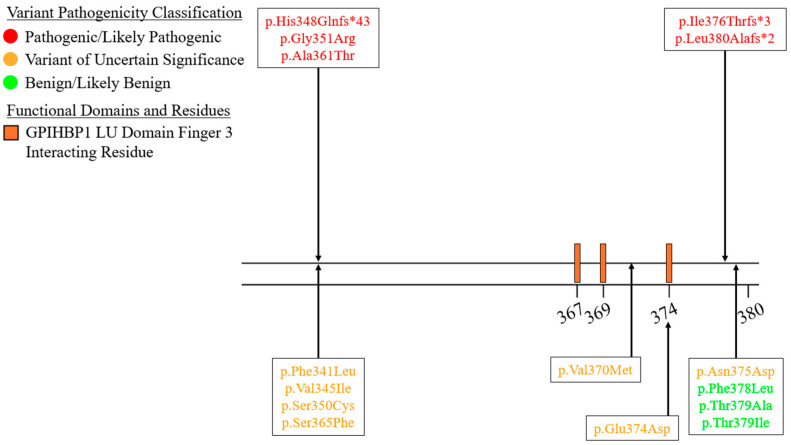
Protein map of reported *LPL* coding sequence variants encoded in exon 7. The number line indicates amino acid residue numbering in the primary structure of newly synthesized LPL peptide, with the specific numbers indicating the beginning and/or end of the functional domain they border. Orange boxes represent residues involved in interacting with finger 3 of the LU domain of GPIHBP1. All variants are color-coded according to their ACMG pathogenicity classification: red indicates pathogenic or likely pathogenic, yellow orange indicates a variant of uncertain significance (VUS), and green indicates benign or likely benign. Pathogenic/likely pathogenic variants are shown above the linear map, while VUS and benign/likely benign variants are shown below. For frameshift variants resulting in a premature stop codon, the notation ‘fs*(number)’ indicates that the frameshift variant results in stop codon at (number) of residues downstream of the variant site.

**Figure 11:**
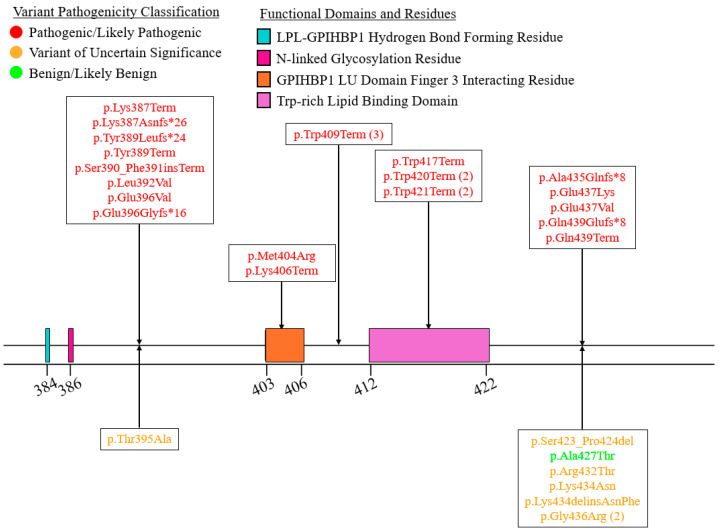
Protein map of reported *LPL* coding sequence variants encoded in exon 8. The number line indicates amino acid residue numbering in the primary structure of newly synthesized LPL peptide, with the specific numbers indicating the beginning and/or end of the functional domain they border. Turquoise boxes represent residues involved in the formation of a stabilizing hydrogen bond between LPL and GPIHBP1. The pink box represents asparagine residues at which N-linked glycosylation has been found to occur. Orange boxes represent residues involved in interacting with finger 3 of the LU domain of GPIHBP1. Light pink-purple boxes represent the Trp-rich lipid-binding domain. All variants are color-coded according to their ACMG pathogenicity classification: red indicates pathogenic or likely pathogenic, yellow orange indicates a variant of uncertain significance (VUS), and green indicates benign or likely benign. Pathogenic/likely pathogenic variants are shown above the linear map, while VUS and benign/likely benign variants are shown below. For frameshift variants resulting in a premature stop codon, the notation ‘fs*(number)’ indicates that the frameshift variant results in stop codon at (number) of residues downstream of the variant site.

**Figure 12:**
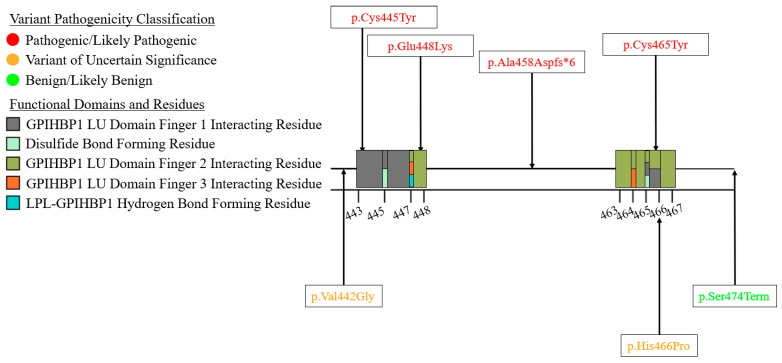
Protein map of reported *LPL* coding sequence variants encoded in exon 9. The number line indicates amino acid residue numbering in the primary structure of newly synthesized LPL peptide, with the specific numbers indicating the beginning and/or end of the functional domain they border. Dark gray boxes represent residues involved in interacting with finger 1 of the LU domain of GPIHBP1. The light-green boxes indicate cysteine residues involved in the formation of intramolecular disulfide bonds. Yellow-green boxes represent residues involved in interacting with finger 2 of the LU domain of GPIHBP1. Orange boxes represent residues involved in interacting with finger 3 of the LU domain of GPIHBP1. Turquoise boxes represent residues involved in the formation of a stabilizing hydrogen bond between LPL and GPIHBP1. All variants are color-coded according to their ACMG pathogenicity classification: red indicates pathogenic or likely pathogenic, yellow orange indicates a variant of uncertain significance (VUS), and green indicates benign or likely benign. Pathogenic/likely pathogenic variants are shown above the linear map, while VUS and benign/likely benign variants are shown below. For frameshift variants resulting in a premature stop codon, the notation ‘fs*(number)’ indicates that the frameshift variant results in stop codon at (number) of residues downstream of the variant site.

**Figure 13:**
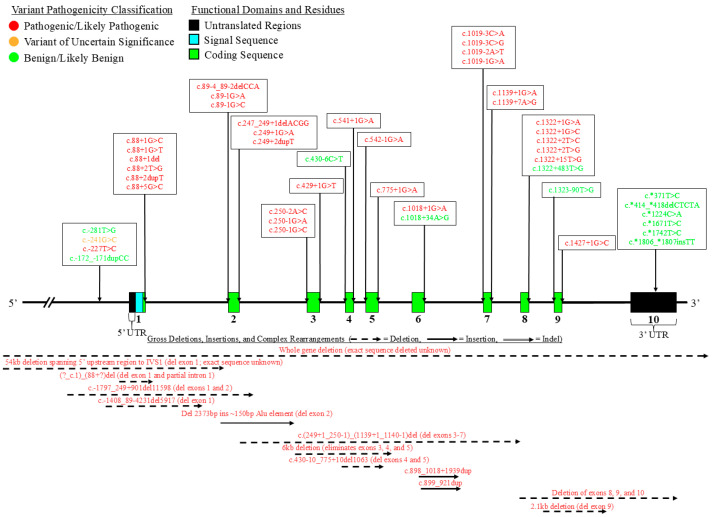
Gene map of reported *LPL* noncoding sequence and large-scale variants. Gene map of *LPL* annotated with variants discovered in regulatory regions 5′ and 3′ untranslated regions (UTRs), promoter regions, etc., splice donor and acceptor sites, and introns. Numbering underneath boxes indicates exons. Black boxes indicate untranslated sequences, blue boxes indicate the sequence encoding the LPL signal peptide, and green boxes indicate coding sequences. For large-scale variants, arrows below the gene map represent the approximate region of the gene affected with variant annotations above the arrows. All variants are color-coded according to their ACMG pathogenicity classification: red indicates pathogenic or likely pathogenic, yellow orange indicates a variant of uncertain significance (VUS), and green indicates benign or likely benign.

We summarized the coding sequence variants resulting in amino acid changes alongside the relative positions of the major functional domains of LPL in Figure 4, Figure 5, Figure 6, Figure 7, Figure 8, Figure 9, Figure 10, Figure 11 and Figure 12, with each figure presenting the region of the LPL peptide encoded by exons 1 to 9, respectively. The nucleic acid changes for noncoding region variants and large-scale variants (i.e., gross deletions, gross insertions, complex rearrangements, etc.) are summarized in Figure 13. A discussion and analysis of the variants summarized in these figures are presented below, with notes on some specific variants of particular interest. Detailed notes for each variant can be found in Supplementary Tables S1 and S2. We also briefly discuss the roles and associations of some benign and likely benign variants for which specific notes for each variant can be found in Supplementary Table S3.

## 7. Coding Region Variants

### 7.1. Exon 1—Signal Peptide

Exon 1 encodes the entirety of the signal peptide and the first few residues of the mature LPL peptide. Within our curated assembly, all variants reported within exon 1 impact codons of the signal peptide, though several do result in frameshifts that lead to premature termination codon downstream of the signal peptide region (Figure 4). Additionally, all pathogenic variants reported within exon 1 are predicted to lead to prematurely truncated proteins (Figure 4).

This includes four variants eliminating the normal translation initiation codon. Of these, the most interesting is the LPL:c.3G>C:p.Met1Ile variant, as two of the in vitro characterizations of LPL activity and mass for the variant peptide have some contradictions. Specifically, the first report of this variant found that the variant had near completely abolished activity at just 2% of the wild-type (WT) level and protein mass at ~3% of WT level [102]. The second study that analyzed this variant in vitro found that the variant has an activity level of 55.93 ± 3.28% with completely abolished protein mass [103], indicating a potential secretion defect. The residual LPL mass level detected in the initial report of this variant may be because it is possible for non-AUG translation initiation codons to be used to initiate translation, though this typically occurs at a reduced efficiency compared to the normal translation initiation codon [104,105]. The protein activity level discrepancy is far more surprising. In theory, if the protein was synthesized and secreted appropriately, then it should not have an activity defect. Further investigation is needed to verify what the true molecular defect is, as currently, an explanation for this discrepancy is not obvious.

The single VUS variant we identified, LPL:c.26T>G:p.Leu9Arg, is quite interesting. The variant has been reported in the heterozygous state in an individual with severe HTG, and the variant was found to be associated with mildly reduced LPL mass [106]. While no direct experimental evidence is available, it is plausible that this variant has an associated true molecular defect, as the mutated residue seems to be part of the hydrophobic core of the signal peptide, with the non-polar Leu at this position considered important in humans and other mammals [107,108]. Thus, the substitution of a charged amino acid like Arg at this position may disrupt the normal functioning and/or folding of the signal peptide. In fact, in at least one analysis [108], it has been demonstrated that polar residues, and likely charged residues by extension, are not found within WT h-motifs, and it seems that this variant lies within an h-motif of the LPL signal peptide. However, experimental confirmation is needed to state for sure whether this variant is truly pathogenic or not.

### 7.2. Exons 2–6—N-Terminal α/β-Hydrolase Domain

Exon 2 encodes several key functional motifs, including two disulfide bond forming residues (Cys54-Cys67) [27,28], the first N-linked glycosylation site (Asn70), which is considered essential for proper LPL folding and functionality [7,109], a portion of a motif that has been demonstrated to interact with both apo C-II and ANGPTL4 [30,78], and the first oxyanion hole forming residue (Trp82) (Figure 5). All but four of the pathogenic variants found in exon 2 are premature truncating variants, which have obvious molecular consequences.

Of the four remaining pathogenic variants, the molecular defect was only determined for two. First, the LPL:c.188C>T:p.Ser63Phe variant was observed in compound heterozygosity with a pathogenic exon 5 variant LPL:c.662T>C:p.Ile221Thr in a patient with lipemic plasma, and an in vitro study in HEK 293 cells determined that this variant decreased LPL activity by ~62%, which was mirrored by ~54% reduction in post-heparin plasma LPL activity in the patient compared to WT [103]. The second is LPL:c.209A>G:p.Asn70Ser which eliminates the first, essential N-linked glycosylation site and was observed in the homozygous state in an LPL deficient patient [7,109].

Exon 3 begins with encoding the remainder of the apo C-II and ANGPTL4 binding motifs encoded in exon 2 and encodes an additional purported apo C-II and ANGPTL4 binding motif [30,78] (Figure 6). Exon 3 contains 38 pathogenic/likely pathogenic coding variants, with 20 of these being premature truncation variants with obvious consequences. The remaining 18 pathogenic variants consist of missense variants, with 7 impacting residues in the 2nd apo C-II and ANGPTL4 binding motif (Figure 6).

While it may appear that a number of these pathogenic missense variants do not directly impact functionally important residues, this is not the case, as a number of hydrophobic residues, including some residues encoded in exon 3, have been purported to be involved in stabilizing and/or guiding the lipid substrate in the active site (See “Protein Structure of LPL” section for more details regarding these residues) [27].

Generally, the pathogenic missense variants of this exon seem to produce one or more of the following molecular defects (as determined by in vitro functional studies): (1) reduced catalytic activity, (2) reduced protein secretion, (3) reduced protein mass, and/or (4) reduced protein stability (Supplementary Table S1). Perhaps the most interesting of these variants is LPL:c.429G>T:p.Glu143Asp. The molecular defect of this variant most likely arises from the nucleotide change and not from the amino acid change, as this variant impacts the final nucleotide of exon 3, which places it in the canonical donor splice site. In silico analyses performed by the original reporters of this variant found that this variant is predicted to decrease the functionality of the adjacent donor splice site, and in turn may activate an alternate donor splice site in intron 3, leading to altered splicing of intron 3 [110]. Thus, while the amino acid change may not be deleterious in and of itself, the DNA change likely is.

Exon 4 encodes three primary sets of functional residues: (1) Ser159 is the first catalytic triad residue [27,28], (2) Leu160 is the final oxyanion hole residue [25,28,77], and (3) many of the other residues in this portion of the protein are hydrophobic residues lining the active site that are purported to be involved in van der Waals interactions with lipid substrate entering the active site [27] (Figure 7).

At the time of writing, only one pathogenic variant of the catalytic triad codon has been reported, LPL:c.476G>C:p.Ser159Thr [111]. The molecular defect of this variant is rather obvious, given that it alters a highly conserved key residue directly involved in the catalytic functionality of LPL [111]. Specifically, it was found that this variant, reported in two homozygous siblings presenting with FCS, was associated with a normal pre-heparin LPL mass, but heparin administration showed no increase in plasma LPL mass, indicating a loss of catalytic function [111].

Similarly, only one pathogenic variant has been reported directly affecting the codon for the final oxyanion hole residue, LPL:c.478C>T:p.Leu160Phe [112]. This variant was reported in the homozygous state in a neonate presenting with FCS [112]. While functional data are not available, it is likely that this variant eliminates the functionality of the oxyanion hole, severely hampering the catalytic functionality of the protein.

Like the LPL:c.429G>T:p.Glu143Asp variant in exon 3, two variants in exon 4, LPL:c.541G>C:p.Gly181Arg and LPL:c.541G>A:p.Gly181Ser, have been observed to impact the final nucleotide. Interestingly, while LPL:c.541G>C:p.Gly181Arg is predicted to result in alternative splicing, LPL:c.541G>A:p.Gly181Ser was shown to not impact splicing at all [113].

The remaining pathogenic coding sequence variants in this exon effect residues that form the active site (Figure 7).

With respect to the VUS variants located within exon 4, the LPL:c.451G>A:p.Asp151Asn is interesting as it was observed to co-segregate with familial combined hyperlipidemia (FCHL) in a pedigree from Iran and was absent in unaffected individuals [114]. Unfortunately, functional characterization data are not available, making it difficult to determine if this variant has a deleterious effect itself or if it is linked to another variant that does.

Exon 5 accounts for a large proportion of all pathogenic coding variants observed in *LPL* (Figure 8). This seems to be due to the large concentration of functional residues and domains encoded by exon 5, as well as the fact that most of the residues encoded by exon 5 form part of the structure of the active site [27,28]. Exon 5 encodes (1) the second catalytic triad residue, Asp183 [27,28], (2) all five residues involved in coordinating a Ca^2+^ into the 3D LPL structure [27], (3) an apo C-II binding motif [30], (4) the lid region [27,28], (5) an ANGPTL4 binding motif [78], and (6) a cysteine residue involved in forming a disulfide bridge [8,27,28].

Notably, most pathogenic/likely pathogenic variants we observed in this exon appear to directly alter functional domains and/or residues. Three separate variants have been reported altering the second catalytic triad residue, of which only LPL:c.547G>A:p.Asp183Asn and LPL:c.548A>G:p.Asp183Gly have been functionally characterized. The former was found to be normally secreted but had essentially zero catalytic activity [115], while the latter was observed in two separate in vitro studies to result in reduced LPL mass and completely abolished activity [115,116]. This difference is most likely explained by the differences in size, structure, and/or electrochemical properties between Asn and Gly. While both variants eliminate the negatively charged Asp, Asn is much more similar in size to Asp, whereas Gly is much smaller, which may explain why LPL:c.548A>G:p.Asp183Gly has an additional mass defect associated with it compared to the LPL:c.547G>A:p.Asp183Asn, for which only catalytic activity is reduced.

Eight variants were found to directly alter residues involved in the coordination of Ca^2+^ ion into the structure of LPL (Figure 8). Of these, four impact residue 197, two impact residue 199, one impacts residue 201, and one impacts residue 202. Based on functional characterizations performed for three of these variants (LPL:c.590G>A:p.Arg197His, LPL:c.596C>G:p.Ser199Cys, and LPL:c.602A>T:p.Asp201Val), it appears that variants altering these Ca^2+^ coordinating residues all lead to reduced or completely abolished secretion of protein [27,117,118], with additional defects observed based on the specific variant (Supplementary Table S1). Supporting this observation is further in vitro functional characterization of alterations to Asp202, which found that LPL:Asp202Glu is not secreted [27]. Interestingly though, it was shown that LPL:Asp201Glu would be tolerated, as while Asp201 interfaces with the Ca^2+^ ion indirectly via an intervening water molecule, Glu at the 201 residue can interface directly with the Ca^2+^ ion by displacing the water molecule [27].

The frameshift variant impacting one of the Ca^2+^ coordinating residues, LPL:c.596delC:p.Ser199Phefs*8, is particularly interesting, as it was discovered in a heterozygous patient with no other TG-elevating variants suffering from recurrent HTG-linked pancreatitis episodes, and it was found that this variant was uniquely associated with an anti-LPL antibody that partially inhibited WT LPL activity in vitro and immunosuppressive therapy via azathioprine treatment significantly reduced plasma TG levels in this patient [101]. As the authors of this report note, it is not known whether the presence of prematurely truncated LPL peptide due to the variant triggered the autoimmune response against LPL [101], but autoantibodies against LPL have been found in patients with autoimmune diseases, such as systemic lupus erythematosus [119,120,121,122]. However, the heterozygous patient carrying the LPL:c.596delC:p.Ser199Phefs*8 variant had no personal history of autoimmune disease, but their father had vitiligo [101]. Furthermore, as Pruneta-Deloche and colleagues note, the autoantibodies against LPL observed in patients with autoimmune disease seem to only result in a ~10% elevation in plasma TG levels, which on its own would be insufficient to induce the severe HTG required to cause pancreatitis [101]. Consequently, they hypothesized that in heterozygous LPL-deficient conditions, such as those produced by heterozygosity for the premature truncation variant LPL:c.596delC:p.Ser199Phefs*8, anti-LPL autoantibodies may induce severe HTG by inhibiting the lower amount of WT LPL available [101]. This presents a unique disease mechanism and is consistent with our findings of secondary factors often being necessary to induce severe HTG in heterozygosity for LOF variants in *LPL* [3].

Interestingly, while functional data are unavailable, the variant affecting the final nucleotide of exon 5, LPL:c.775G>A:p.Asp259Asn, is predicted to result in alternative splicing by both MES and SpliceAI (Supplementary Table S1), as the final nucleotide of exon 5 lies within the canonical donor splice site for intron 5.

The pathogenic LOF variant, LPL:c.644G>A:p.Gly215Glu, is perhaps the quintessential example of a LOF variant in *LPL*, having been discussed in several studies [2,123,124]. This variant illustrates a point of interest regarding population-specific consequences. For instance, this variant has shown a founder effect in the French-Canadian population, leading to a higher allele frequency, making it a relatively more common cause of FCS in this population [125].

Exon 6 contains the largest number of pathogenic coding variants (Figure 9). Exon 6 encodes several key functional residues/domains: (1) a portion of the lid region [27,28], (2) several disulfide bridge-forming residues [8,27,28], and (3) the final catalytic triad residue, His268 [27,28]. The other residues encoded in this exon appear to contribute to the active site structure [27,28].

Two variants, LPL:c.802C>T:p.His268Tyr and LPL:c.804C>A:p.His268Gln, were observed to impact the final catalytic triad residue (Figure 9). Direct functional data are not available for these variants, but analysis of LPL protein expression in a proband homozygous for LPL:c.802C>T:p.His268Tyr found abolished protein expression [126].

Like the variant affecting the final nucleotide of exon 5, the variant affecting the final nucleotide of exon 6, LPL:c.1018G>T:p.Val340Phe, is also predicted to result in alternative splicing (Supplementary Table S2). However, unlike the variant in exon 5, this variant is currently considered VUS due to a paucity of evidence that can be used to assess its pathogenicity.

Most variants in exon 6 do not directly affect residues forming functional domains (Figure 9), but functional characterizations for many of these variants (summarized in Supplementary Table S1 with references) consistently found that variants in this region result in reduced or completely abolished catalytic activity, with additional defects in synthesis and secretion also observed for some variants. This is consistent with the fact that exon 6 encodes a large portion of the active site of mature LPL.

### 7.3. Exons 7–9—C-Terminal Lipid Binding Domain

Exon 7 has very few pathogenic variants—five in total (Figure 10). This is perhaps in line with the fact that exon 7 only contains a small number of directly functionally important residues, namely residues 367, 369, and 374, which are involved in the interaction between LPL and finger 3 of the GPIHBP1 LU domain [27].

Of the five pathogenic coding variants reported in this exon, three are premature truncating variants with rather obvious molecular consequences. The remaining two are missense variants (LPL:c.1051G>A:p.Gly351Arg and LPL:c.1081G>A:p.Ala361Thr), but functional data are not available for either of them. They are considered pathogenic as both have been reported to produce FCS in the homozygous state [127,128,129].

Functional data are unavailable for all but one VUS variant, LPL:c.1094C>T:p.Ser365Phe. While this variant was observed in a patient with normal post-heparin plasma LPL activity and mass, an in vitro experiment found that this variant mildly impaired protein secretion but simultaneously increased specific activity of the enzyme [130]. This increased specific activity was concluded to be sufficient to compensate for the reduced protein secretion [130]. However, it is plausible that additional factors elevating plasma TGs and/or impacting LPL functionality may overwhelm this compensatory effect, leading to a net deleterious impact for this variant.

Exon 8 contains the bulk of the functional residues and domains of the C-terminal lipid-binding domain (Figure 11). Exon 8 encodes (1) several GPIHBP1 interacting residues, including a residue that forms a hydrogen bond with GPIHBP1 and residues involved in interacting with finger 3 of the GPIHBP1 LU domain [27], (2) the final N-linked glycosylation site, Asn386 [27,28], and (3) the Trp-rich lipid-binding domain formed by residues 412–422 [27,28,79].

Most pathogenic variants in this exon are premature truncating variants, with only five pathogenic missense variants observed (Figure 11). Of these five, functional data supporting a deleterious effect is available for LPL:c.1187A>T:p.Glu396Val [131], LPL:c.1211T>G:p.Met404Arg [97], LPL:c.1309G>A:p.Glu437Lys [132], and LPL:c.1310A>T:p.Glu437Val [100]. LPL:c.1310A>T:p.Gly437Val is particularly interesting, as it was found that this variant renders the LPL peptide susceptible to cleavage by the endoproteolytic enzyme furin at residue 324, a known furin cleavage site in LPL [100]. This presents a potential post-translational mechanism of disease, which is supported by the finding of FCS in a homozygote for this variant, in which LPL activity was reduced to 11% of WT level [133].

Exon 9 has the fewest pathogenic variants observed in any exon: four in total (Figure 12). The major functional features encoded in exon 9 are a number of GPIHBP1 interacting residues, as well as the final two disulfide bridge forming residues (Cys445–Cys465) [27,28]. All four pathogenic variants are missense variants. However, of these, only the LPL:c.1334G>A:p.Cys445Tyr and LPL: c.1342G>A:p.Glu448Lys variants have been previously reported in the literature (Supplementary Table S1). It has been demonstrated that both of these variants abolish the ability of LPL to bind to GPIHBP1 while retaining the functionality of the N-terminal α/β hydrolase domain [134]. While other studies have observed a reduction in LPL activity due to these variants, a similar reduction in LPL mass was also observed [135,136], which would indicate that the specific activity of LPL is unchanged, which supports the finding that the functionality of the N-terminal α/β hydrolase domain is retained. As a result of the loss of GPIHBP1 binding, LPL transport to the apical surface is eliminated, leading to the loss of plasma LPL-mediated lipolysis [134].

## 8. Non-Coding and Large-Scale Variants

Figure 13 outlines the non-coding and large-scale (gross deletions, gross insertions, and large-scale complex rearrangements) variants reported in *LPL* to be associated with and/or produce disease. To the best of our knowledge, only one regulatory region variant in *LPL* reported in the literature is not considered benign or likely benign according to the ACMG guidelines, LPL:c.-227T>C [137]. This variant was reported in a compound heterozygous state with LPL:c.-281T>G in a patient diagnosed with familial combined hyperlipidemia and reduced post-heparin plasma LPL activity, with this variant specifically impacting an Oct-1 transcription factor binding site leading to reduction in transcriptional activity of the variant promoter to less than 15% of WT [137].

With respect to splicing variants, of the 29 pathogenic variants found to affect either the donor or acceptor splice sites in *LPL*, the most affected intron is intron 1, with 6 variants impacting the donor splice site and three affecting the acceptor splice site (Figure 13). All pathogenic/likely pathogenic splicing variants have been demonstrated or strongly predicted to abolish the affected splice site functionality, leading to alternative pre-mRNA splicing (Supplementary Table S1).

With respect to large-scale variants, there are 10 gross deletions, two gross insertions, and one complex rearrangement for a total of 13 included in our curated list (Figure 13). Apart from the two gross insertion variants, all of these variants lead to loss of one or more exons. Rather interesting is the complex rearrangement variant, which, to the best of our knowledge, presents a novel mutational mechanism by which variants in *LPL* may occur. Specifically, a novel variant leading to LPL deficiency was reported by Okubo and colleagues resulting from *Alu* retro transposition, producing a complex large indel [96]. The major consequence of this variant was the deletion of exon 2 [7,109], and the beginning portion of the first apo C-II and ANGPTL4 binding motif [30,78] (Figure 13). While this appears to be the only *LPL* variant reported due to such a mechanism, this is nevertheless evidence of a novel mechanism by which LPL deficiency may occur.

## 9. Benign/Likely Benign Variants: Deleterious and Gain-of-Function Variants

We include here a discussion of 21 select benign/likely benign variants for the purpose of highlighting the role of these common variants and various features of the *LPL* gene in determining the plasma TG phenotype (Supplementary Table S3).

Most of these variants are common, with only three variants considered to be rare. The first of these, LPL:c.182C>T:p.Ala61Val, was found to likely be in linkage disequilibrium with the pathogenic splicing variant LPL:c.250-1G>C [2]. The second, LPL:c.1134C>G:p.Phe378Leu, was shown to have no effect on mass or activity in in vitro studies, but it was observed in a heterozygote with severe HTG [138]. The third, LPL:c.*414_*418delCTCTA, was shown to modestly reduce *LPL* gene expression and very slightly reduce translation, likely due to the fact that this small deletion in the 3′UTR disrupts a putative insulin response element [139].

While several of the common benign/likely benign variants we list have been associated with HTG and/or functional defects, such as reduced promoter activity, catalytic activity, etc., the fact that these variants are relatively common in population databases indicates that they are not true LOF variants (Supplementary Table S3). For example, LPL:c.953A>G:p.Asn318Ser has been shown in multiple in vitro studies to reduce LPL catalytic activity [140,141,142] and has also been reported to be modestly associated with HTG [42,142,143,144]. However, the variant is extremely common in population databases, and 36 homozygous individuals have been reported in gnomAD [145], which indicates that even if there is a deleterious effect for this variant, it is small and not enough to qualify as an LOF variant.

Nevertheless, several of these common benign/likely benign variants associated with a deleterious effect are quite interesting, such as LPL:c.-281T:G and LPL:c.1322+483T>G. The promoter variant LPL:c.-281T>G was shown to reduce the activity of the *LPL* promoter in in vitro experiments [137,146]. Interestingly though, LPL:c.-281T>G was later associated with a protective effect on plasma TG levels during the third trimester of pregnancy in women of African American descent [147]. The intron 8 variant LPL:c.1322+483T>G was shown to be associated with a ~20% reduction in gene transcription, likely due to this variant altering a transcription factor binding site in intron 8 [148]. This, alongside the discovery of a common gain-of-function (GOF) variant in intron 8, LPL:c.1323-90T>G, that increases gene expression by ~1.7 times the WT level [149] demonstrate the importance of intron 8 in regulating *LPL* expression.

While our list of variants is not necessarily comprehensive, there is an interesting trend in that nearly all common GOF variants in our list are concentrated toward the 3′-end of the coding region of the *LPL* gene, with most located in exons 9 and 10, the latter of which encodes the 3′ UTR (Figure 13 and Supplementary Table S3).

Perhaps the most notable of these GOF variants is LPL:1421C>G:p.Ser474Term (also commonly known as p.Ser474Ter), a common GOF nonsense variant. This variant has been observed in an extremely large number of studies [42,143,150,151,152,153,154,155,156,157,158,159,160,161,162,163,164,165,166,167,168,169,170,171,172,173,174,175,176,177,178,179,180,181,182,183,184,185,186,187,188,189,190,191,192,193,194,195] and has been reported to increase LPL mass and activity [196] and reduce the inhibition of translation [172]. Interestingly, it has also been shown that this variant is in linkage disequilibrium with a separate TG-lowering haplotype, which exerts its effect by abolishing miRNA-mediated post-transcription inhibition of LPL [197,198]. It has been suggested that, at least in part, the association of LPL:1421C>G:p.Ser474Term with this haplotype explains its TG-lowering effect [197]. Several of the common 3′ UTR variants included in our list are part of this haplotype (Supplementary Table S3).

## 10. Conclusions

LPL is an extremely important regulator of plasma TG metabolism. Here, we summarized the current understanding of the regulation, expression, structure, and physiological roles of LPL as they relate to plasma TG metabolism, and we provided a comprehensive overview of genetic variation in LPL as it pertains to HTG. We consider this curation and archiving of *LPL* DNA variants to be the first draft for future efforts to create a public reference database for both research and clinical purposes. With respect to the latter, there is growing interest in the potential clinical relevance of *LPL* gene variants detected by clinical next-generation DNA sequencing efforts in patients with various HTG phenotypes, particularly those who are suspected to have FCS. We hope that our present curation effort will serve as a bridge towards a definitive *LPL* variant reference list assembled via a sanctioned and recognized variant curation expert panel and process.

## Figures and Tables

**Table 1:** Summary of coding region pathogenic/likely pathogenic and VUS variants in *LPL*.

	Variant Type (P-LP ^1^/VUS ^2^)					
Genomic Location	Missense	Nonsense	Small Deletion	Small Insertion	Small Indel	Total
**Exon 1**	4/1	2/0	3 */0	2/0	1/0	12/1
**Exon 2**	4/10	2/0	6/0	5/0	0/0	17/10
**Exon 3**	18/4	7/0	6/0	4/0	3/0	38/4
**Exon 4**	11/2	1/0	4/0	0/0	0/0	16/2
**Exon 5**	51/10	4/0	11/0	1/0	0/0	67/10
**Exon 6**	46/14	10/0	12/0	4/0	0/0	72/14
**Exon 7**	2/7	0/0	3/0	0/0	0/0	5/7
**Exon 8**	5/5	10/0	5/1	3/0	0/1	23/7
**Exon 9**	3/2	0/0	1/0	0/0	0/0	4/2
**Total**	144/55	36/0	51/1	19/0	4/1	254/57

^1^ P-LP = pathogenic/likely pathogenic. ^2^ VUS = variant of uncertain significance. * One of these small deletion variants also affects the first nucleotide of intron 1.

**Table 2:** Summary of non-coding region pathogenic/likely pathogenic and VUS variants in *LPL*.

	Variant Type (P-LP ^1^/VUS ^2^)				
Genomic Location	Splicing	Small Deletion	Small Insertion	Other	Total
**Regulatory** **(Promoter, 5′ UTR, and 3′ UTR)**	N/A ^3^	0/0	0/0	1/1	1/1
**Intron 1**	7/0	1/0	1/0	N/A	9/0
**Intron 2**	5 */0	0/0	1/0	N/A	6/0
**Intron 3**	1/0	0/0	0/0	N/A	1/0
**Intron 4**	2/0	0/0	0/0	N/A	2/0
**Intron 5**	1/0	0/0	0/0	N/A	1/0
**Intron 6**	5/0	0/0	0/0	N/A	5/0
**Intron 7**	2/0	0/0	0/0	N/A	2/0
**Intron 8**	5/0	0/0	0/0	N/A	5/0
**Intron 9**	1/0	0/0	0/0	N/A	1/0
**Total**	29/0	1/0	2/0	1/0	33/1

^1^ P-LP = pathogenic/likely pathogenic. ^2^ VUS = variant of uncertain significance. ^3^ N/A = not applicable. * One of these small deletion variants also affects the first nucleotide of intron 1.

**Table 3:** Summary of pathogenic/likely pathogenic and VUS large-scale variants in *LPL*.

Variant Type (P-LP ^1^/VUS ^2^)		
Gross Deletions	Gross Insertions	Complex Rearrangements
10/0	2/0	1/0

^1^ P-LP = pathogenic/likely pathogenic. ^2^ VUS = variant of uncertain significance.

## Data Availability

Additional data are available upon request from R.A.H.

## Supplementary Materials

The following supporting information can be downloaded at: https://www.mdpi.com/article/10.3390/genes16010055/s1, Supplementary Table S1: Curated list of pathogenic/likely pathogenic variants in LPL; Supplementary Table S2: Curated list of variants of uncertain significance in LPL; Supplementary Table S3: Select benign/likely benign variants reported in association with various TG phenotypes; Supplementary Table S4: LPL variants reported in HGMD excluded from analysis and review due to unavailability of case data.
