# Supplementary material for: Lipoprotein Lipase: Structure, Function, and Genetic Variation
Source: Genes (Basel). 2025 Jan 5;16(1):55. doi: 10.3390/genes16010055 (PMC11764694; doi:10.3390/genes16010055)
Supplement: Supplementary file 1 [file genes-16-00055-s001.zip › genes-3401157-supplementary.pdf]

Supplementary Table S1. Curated List of Pathogenic/Likely Pathogenic Variants in *LPL*.

Variant Type	Exon/ Intron	Nucleotide Change[b]	Amino Acid Change	ACMG Classification	Molecular Defect Notes (and others where needed)	Previously reported in literature?	ClinVar ID
**Gross Deletion**	N/A[a]	(incl. entire gene)	N/A	Pathogenic	Whole gene deletion.	Yes [1]	-
**Gross Deletion**	N/A	(54kb 5-prime to upstream IVS1)	N/A	Pathogenic	Eliminates a large region upstream of the *LPL* gene (including the promoter region), exon 1, and a portion of intron 1.	Yes [2]	-
**Gross Deletion**	N/A	(?_c.1)_(88+?)del	N/A	Likely Pathogenic	Eliminates exon 1 and a portion of intron 1. Breakpoints unknown.	No (reported in ClinVar)	1459737
**Gross Deletion**	N/A	c.-1797_249+901del11598 (11598 bp incl. ex. 1-2)	N/A	Likely Pathogenic	Eliminates the 5'UTR, exon 1, intron 1, exon 2, and a small portion of intron 2.	Yes [3]	-
**Gross Deletion**	N/A	c.-1408_89-4231del5917 (5917 bp incl. ex. 1)	N/A	Likely Pathogenic	Eliminates exon 1 and a portion of intron 1.	Yes [3]	-
**Regulatory**	N/A	c.-227T>C	N/A	Likely Pathogenic	*In vitro* studies found that this variant reduces transcriptional activity of the *LPL* promoter to less than 15% of wild-type, likely due to altering the binding site for the transcription factor Oct-1 [4].<br><br>Variant was observed in a heterozygote diagnosed with familial combined hyperlipidemia (FCHL) [4].	Yes [4]	1549
**Missense**	Exon 1	c.1A>C	p.Met1Leu	Likely Pathogenic	Alters/eliminates normal translation initiation start codon.	Yes [5]	-
**Missense**	Exon 1	c.2T>C	p.Met1Thr	Likely Pathogenic	Alters/eliminates normal translation initiation start codon.	No (reported in LOVD3)	-
**Missense**	Exon 1	c.3G>C	p.Met1Ile	Likely Pathogenic	Alters/eliminates normal translation initiation start codon.	Yes [6,7]	-
**Missense**	Exon 1	c.3G>A	p.Met1Ile	Likely Pathogenic	Alters/eliminates normal translation initiation start codon.	No (reported in ClinVar)	2006935
**Small Insertion**	Exon 1	c.10_11insTTCG	p.Lys4Ilefs*38	Likely Pathogenic	Altered reading frame leads to premature truncation eliminating ~91.4% of peptide sequence.	Yes [8]	-
**Small Deletion**	Exon 1	c.10_19del	p.Lys4Serfs*3	Pathogenic	Altered reading frame leads to premature truncation eliminating ~98.7% of the peptide sequence.	No (reported in ClinVar)	1455947

**Small Insertion**	Exon 1	c.32dupT	p.Ala12Glyfs*29	Likely Pathogenic	Altered reading frame leads to premature truncation eliminating ~91.6% of peptide sequence.	Yes [9]	-
**Nonsense**	Exon 1	c.41G>A	p.Trp14Term	Pathogenic	Substitution produces premature stop codon leading to elimination of ~97.3% of the peptide sequence.	Yes [10,11]	643218
**Nonsense**	Exon 1	c.42G>A	p.Trp14Term	Likely Pathogenic	Substitution produces premature stop codon leading to elimination of ~97.3% of the peptide sequence.	Yes [10,11]	-
**Small Deletion**	Exon 1	c.46_47delCA	p.Gln16Glufs*24	Pathogenic	Altered reading frame leads to premature truncation variant eliminating ~91.8% of the peptide sequence.	Yes [12–14]	1431010
**Small Indel**	Exon 1	c.58_71delinsCCTC	p.Ala20Profs*21	Pathogenic	Altered reading frame leads to premature truncation variant eliminating ~91.6% of the peptide sequence.	No (reported in ClinVar)	1749778
**Small Deletion**	Exon 1/ Intron 1	c.77_88+1del13	p.Ala26Lysfs*13	Likely Pathogenic	Altered reading frame leads to premature truncation variant eliminating ~92% of the peptide sequence. Interestingly, while it initially appears that this variant may impact splicing of intron 1, it was determined that intron 1 splicing was identical between wild-type pre-mRNA and the pre-mRNA of this variant [9].	Yes [9]	-
**Splicing**	Intron 1	c.88+1G>C	N/A	Pathogenic	Abolishes splice donor site for intron 1. Likely leading to defective pre-mRNA splicing.	Yes [15,16]	1542
**Splicing**	Intron 1	c.88+1G>T	N/A	Pathogenic	Abolishes splice donor site of intron 1, likely leading to defective pre-mRNA splicing.	Yes [17–19]	1685928
**Splicing**	Intron 1	c.88+1del	N/A	Pathogenic	Abolishes splice donor site of intron 1, likely leading to defective pre-mRNA splicing.	No (reported in ClinVar)	2823414
**Splicing**	Intron 1	c.88+2T>G	N/A	Likely Pathogenic	Abolishes splice donor site of intron 1, likely leading to defective pre-mRNA splicing.	Yes [18]	-
**Small Insertion**	Intron 1	c.88+2dupT	N/A	Likely Pathogenic	Abolishes the splice donor site of intron 1, likely leading to defective pre-mRNA splicing.	Yes [20–23]	2735123
**Splicing**	Intron 1	c.88+5G>C	N/A	Likely Pathogenic	Functional data are unavailable.<br><br>This variant has been reported in compound heterozygosity with the pathogenic missense variant LPL:c.644G>A:p.Gly215Glu to cause familial chylomicronemia syndrome (FCS) [24], indicating that this variant has a deleterious effect, likely by altering the proper splicing of intron 1.	Yes [12,24]	-
**Complex Rearrangement**	N/A	(Del 2373bp incl ex.2, ins ~150bp Alu element)	N/A	Pathogenic	Eliminates exon 2.<br><br>Reported in a homozygous patient presenting with complete LPL deficiency [25]. Appears to be the first and, to date, only report of a complex deletion-insertion event mediated by Alu retrotransposition [25].	Yes [25]	-
**Small Deletion**	Intron 1	c.89-4_89-2delCCA	N/A	Likely Pathogenic	Alters acceptor splice site of intron 1 which was found to result in skipping of exon 2 during splicing, leading to	Yes [26]	-

					production of a premature stop codon 6 codons downstream in exon 3 [26].<br><br>Variant was observed in a FCS patient found to be a compound heterozygote for this variant and the pathogenic missense variant LPL:c.628C>G:His210Asp [26].		
**Splicing**	Intron 1	c.89-1G>A	N/A	Pathogenic	Abolishes the acceptor splice site of intron 1, likely leading to defective pre-mRNA splicing.	No (reported in ClinVar)	1494261
**Splicing**	Intron 1	c.89-1G>C	N/A	Pathogenic	Abolishes the acceptor splice site of intron 1, likely leading to defective pre-mRNA splicing.<br><br>Variant was observed in a homozygous individual diagnosed with FCS [20].	Yes [20]	1492400
**Small Insertion**	Exon 2	c.90_91insG	p.Arg31Glufs*10	Likely Pathogenic	Altered reading frame leads to premature truncation eliminating ~91.6% of the peptide sequence.	No (reported in LOVD3)	-
**Small Deletion**	Exon 2	c.91_95del	p.Arg31Phefs*7	Pathogenic	Altered reading frame leads to premature truncation eliminating ~92.2% of the peptide sequence.	No (reported in our clinic)	-
**Small Deletion**	Exon 2	c.94_98delAGAGA	p.Arg32Phefs*7	Pathogenic	Altered reading frame leads to premature truncation eliminating ~92% of the peptide sequence.	Yes [12,27]	950321
**Small Insertion**	Exon 2	c.127dup	p.Leu43Profs*5	Pathogenic	Altered reading frame leads to premature truncation eliminating ~90.1% of the peptide sequence	No (reported in ClinVar)	2793081
**Small Insertion**	Exon 2	c.128dupT	p.Arg44Lysfs*4	Pathogenic	Altered reading frame leads to premature truncation eliminating ~90.1% of peptide sequence	Yes [12,28]	-
**Small Deletion**	Exon 2	c.133_143del11	p.Thr45Hisfs*3	Likely Pathogenic	Altered reading frame leads to premature truncation eliminating ~90.1% of peptide sequence.	Yes [29]	-
**Small Insertion**	Exon 2	c.133dupA	p.Thr45Asnfs*3	Likely Pathogenic	Altered reading frame leads to premature truncation eliminating ~90.1% of peptide sequence.	Yes [5]	-
**Small Deletion**	Exon 2	c.138del	p.Glu47Lysfs*14	Pathogenic	Altered reading frame leads to premature truncation eliminating ~87.4% of the peptide sequence.	No (reported in ClinVar)	1399464
**Small Deletion**	Exon 2	c.147del	p.Ala50Leufs*11	Pathogenic	Altered reading frame leads to premature truncation eliminating ~87.4% of the peptide sequence.	No (reported in ClinVar)	2863224
**Nonsense**	Exon 2	c.162C>A	p.Cys54Term	Likely Pathogenic	*In vitro* study in COS-7 cells revealed that this variant resulted in significantly reduced LPL mRNA expression compared to wild-type and significantly reduced enzymatic activity in both cell lysate and cell medium, but there were no significantly changes in LPL mass [30].	Yes [30,31]	-

					This substitution produces premature stop codon leading to elimination of ~88.8% of the protein sequence. This variant has been reported in compound heterozygosity with the pathogenic missense variant LPL:c.835C>G:p.Leu279Val in one LPL deficiency case [31] and with the likely pathogenic splicing variant LPL:c.1322+1G>A in a case of severe HTG with acute pancreatitis [30].		
**Small Insertion**	Exon 2	c.183dupA	p.Glu62Argfs*28	Likely Pathogenic	Altered reading frame leads to premature truncation eliminating ~81.3% of peptide sequence.	Yes [12,24,32]	-
**Missense**	Exon 2	c.188C>T	p.Ser63Phe	Likely Pathogenic	*In vitro* study in HEK 293 cells found that this variant led to a ~62% reduction in LPL activity compared to wild-type control [7].<br><br>This variant was observed in compound heterozygosity with the pathogenic missense LPL:c.662T>C:p.Ile221Thr in a patient with lipemic plasma [7].	Yes [7]	-
**Missense**	Exon 2	c.209A>G	p.Asn70Ser	Pathogenic	*In vitro* study in HEK 293 cells revealed that this variant results in abolished LPL activity and additionally, the variant peptide was not secreted into the cell medium, only being detected intracellularly [33]. The likely mechanism is because this variant eliminates the first N-linked glycosylation site of LPL, which has been previously shown to be essential for proper synthesis, folding, and secretion of LPL [34,35].<br><br>This variant was initially reported in a homozygous patient diagnosed with FCS [33] and we have also observed an FCS case that was also found to be homozygous for this variant in our own clinic as well [12].	Yes [12,33]	-
**Small Deletion**	Exon 2	c.214_224del	p.Ser72Leufs*14	Pathogenic	Altered reading frame leads to premature truncation eliminating ~82.1% of the peptide sequence.	No (reported in ClinVar)	2769182
**Missense**	Exon 2	c.239A>G	p.His80Arg	Likely Pathogenic	Functional data are unavailable.<br><br>Variant was identified in compound heterozygosity with the pathogenic missense variant LPL:c.644G>A:p.Gly215Glu in an severe HTG patient [36].	Yes [36,37]	2307988
**Missense**	Exon 2	c.242G>A	p.Gly81Asp	Pathogenic	Functional data are unavailable.<br><br>Variant was initially reported in the homozygous state in an infant with FCS [38]. The initial reporters of this variant speculated that, given the residue altered by this variant forms a portion of the β5 loop of LPL, which has been noted to undergo key conformational changes in the	Yes [12,38]	1339047

					presence of substrate [39], it is possible that the introduction of a new negative charge in this loop by this variant may interfere with this functionality [38].		
**Nonsense**	Exon 2	c.245G>A	p.Trp82Term	Likely Pathogenic	Substitution produces a premature stop codon leading to elimination of ~82.9% of the peptide sequence.<br><br>This variant was reported in compound heterozygosity with the pathogenic missense variant, LPL:c.835C>G:p.Leu279Val in a patient reported to have recurrent pancreatitis secondary to hypertriglyceridemia [40].	Yes [40]	-
**Splicing**	Exon 2/Intron 2	c.247_249+1delACGG	N/A	Likely Pathogenic	Abolishes the donor splice site of intron 2, likely leading to defective pre-mRNA splicing.<br><br>Variant was observed in two homozygotes presenting with severe HTG [41].	Yes [41]	-
**Splicing**	Intron 2	c.249+1G>A	N/A	Pathogenic	Abolishes the donor splice site of intron 2, likely leading to defective pre-mRNA splicing.<br><br>Variant was initially reported in two siblings, both homozygous for this variant, but interestingly, the proband had severe HTG while his sister had mild-to-moderate HTG with both demonstrating reduced LPL activity [42]. It was also found that in both of these patients, essentially no LPL enzyme mass was detected in their post-heparin plasma [42].	Yes [17,20,21,42,43]	1535
**Gross Deletion**	N/A	c.(249+1_250-1)_(1139+1_1140-1)del (ex. 3-7)	p.Val84Alafs*2	Likely Pathogenic	Eliminates exons 3-7, but the exact break points are unknown. This leads to frameshift, producing a premature stop codon.	Yes [44]	-
**Small Insertion**	Intron 2	c.249+2dupT	N/A	Likely Pathogenic	Abolishes the donor splice site of intron 2, likely leading to defective pre-mRNA splicing.<br><br>Specifically, it was predicted that this variant disrupts the consensus splice donor site sequence of intron 2, leading to aberrant splicing that adds 38 nucleotides to exon 2 thereby leading to a frameshift culminating in a premature stop codon 13 amino acids into exon 3 [45]. This would eliminate ~79.6% of the peptide sequence. Variant was reported in a heterozygous proband with severe HTG [45].	Yes [45]	-
**Gross Deletion**	N/A	NG_008855.2:g.(51625_55052)_(57638_59124)del	N/A	Pathogenic	Eliminates exons 3-5.	Yes [46]	-

**Splicing**	Intron 2	c.250-2A>C	N/A	Likely Pathogenic	Disrupts the acceptor splice site of intron 2, likely leading to aberrant splicing.	No (reported in ClinVar)	2843115
**Splicing**	Intron 2	c.250-1G>A	N/A	Likely Pathogenic	Predicted to abolish intron 2 splice acceptor site leading aberrant splicing.	Yes [47]	1526
**Splicing**	Intron 2	c.250-1G>C	N/A	Likely Pathogenic	Predicted to abolish intron 2 splice acceptor site leading to aberrant splicing.<br><br>This variant was in an FCS patient that was a double homozygote for this variant and for the likely benign missense variant LPL:c.182C>T:p.Ala61Val [24]. It is likely that this variant is in linkage disequilibrium with the LPL:c.250-1G>C splicing variant.	Yes [12,18]	-
**Nonsense**	Exon 3	c.264T>A	p.Tyr88Term	Pathogenic	Substitution produces a premature stop codon leading to elimination of ~81.7% of the peptide sequence.	Yes [42,48]	1536
**Nonsense**	Exon 3	c.272G>A	p.Trp91Term	Pathogenic	This substitution produces a premature stop codon leading to elimination of ~81.1% of the peptide sequence,	Yes [12,49,50]	1541
**Missense**	Exon 3	c.272G>C	p.Trp91Ser	Likely Pathogenic	Functional data unavailable.<br><br>This variant has been reported in homozygous state from our clinic to produce FCS symptoms.	No (reported in our clinic and ClinVar)	2662202
**Missense**	Exon 3	c.286G>C	p.Val96Leu	Pathogenic	*In vitro* study in COS cells revealed that this variant does not impact protein mass nor protein stability and only impacts specific catalytic activity, reducing it to ~20% of the normal level [51].<br><br>Variant was first reported in an FCS patient that was found to be compound heterozygous for this variant and the pathogenic missense variant LPL:c.644G>A:p.Gly215Glu [51].	Yes [12,17,27,51–53]	1284647
**Small Deletion**	Exon 3	c.287_288delTG	p.Val96Glyfs*51	Likely Pathogenic	Altered reading frame leads to premature truncation eliminating ~69.3% of peptide sequence.	Yes [54]	-
**Small Indel**	Exon 3	c.289_294delinsTTTGCCAAAA	p.Ala97Phefs*52	Likely Pathogenic	Altered reading frame leads to premature truncation eliminating ~68.8% of peptide sequence.	Yes [18]	968107
**Small Indel**	Exon 3	c.290_293delCCGCinsGG	p.Ala97Glyfs*50	Likely Pathogenic	Altered reading frame leads to premature truncation eliminating ~69.3% of peptide sequence	Yes [55]	-
**Missense**	Exon 3	c.292G>A	p.Ala98Thr	Pathogenic	*In vitro* study in COS-1 cells found that this variant has reduced catalytic activity, reduced secretion, and reduced total LPL mass synthesized compared to wild-type [56].<br><br>Variant has been observed in a severe HTG patient found to be compound heterozygous for this variant and the	Yes [8,12,13,27,56–59]	1203042

					pathogenic missense variant LPL:c.835C>G:p.Leu279Val [57].<br><br>A likely pathogenic missense variant, LPL:c.293C>T:p.Ala98Val, has been observed impacting this codon, which provides additional indirect evidence for a deleterious impact of altering this codon.		
**Small Deletion**	Exon 3	c.292_295delGCCC	p.Ala98Cysfs*73	Likely Pathogenic	Altered reading frame leads to premature truncation eliminating ~64.2% of peptide sequence.	Yes [55]	-
**Missense**	Exon 3	c.293C>T	p.Ala98Val	Likely Pathogenic	Functional data unavailable.<br><br>We have observed this variant in our clinic in patients sequenced on LipidSeq [12], with multiple HTG cases observed.<br><br>A pathogenic missense variant, LPL:c.292G>A:p.Ala98Thr, has been observed impacting this codon, which provides additional indirect evidence for a deleterious impact of altering this codon.	Yes [12]	-
**Nonsense**	Exon 3	c.300C>A	p.Tyr100Term	Pathogenic	Substitution produces a premature stop codon leading to elimination of ~79.2% of the peptide.	Yes [60]	1531
**Missense**	Exon 3	c.305G>C	p.Arg102Thr	Likely Pathogenic	*In vitro* study in COS-7 cells revealed that this variant is expressed at normal levels, but is not secreted from cells, and it demonstrates significantly reduced enzymatic activity as well [61].<br><br>Variant was observed in compound heterozygosity with the likely pathogenic frameshift variant LPL:c.334dupG:p.Asp112Glyfs*36 in a 1-month old child with severe HTG, indicating FCS [61].<br><br>Another likely pathogenic missense variant, LPL:c.306A>C:p.Arg102Ser, has been observed impacting this codon, which provides additional indirect evidence for a deleterious impact of altering this codon.	Yes [61]	2988064
**Missense**	Exon 3	c.306A>C	p.Arg102Ser	Likely Pathogenic	*In vitro* studies in COS-1 cells revealed that this variant produces catalytically inactive protein that is normally secreted [60]. Interestingly, it was also observed that a small amount of catalytically competent variant LPL protein produced from this variant was secreted [60]. Thus, it was hypothesized that this variant impacts catalytic activity of the produced enzyme by impacting stability, hence the small amount of catalytically competent variant protein observed *in vitro* [60].	Yes [60]	1545

					Variant was observed in two patients with compound heterozygosity for the pathogenic nonsense variant LPL:c.300C>A:p.Tyr100Term in LPL and this variant and both were observed to have severe HTG, with the proband also experiencing multiple bouts of pancreatitis [60].<br><br>Another likely pathogenic missense variant, LPL:c.305G>C:p.Arg102Ser, has been observed impacting this codon, which provides additional indirect evidence for a deleterious impact of altering this codon.		
**Small Deletion**	Exon 3	c.312delA	p.Asp105Thrfs*67	Likely Pathogenic	Altered reading frame leads to premature truncation eliminating ~64% of peptide sequence.	Yes [62]	-
**Missense**	Exon 3	c.326T>C	p.Ile109Thr	Likely Pathogenic	Functional data are unavailable.<br><br>Variant has been observed in multiple homozygous individuals diagnosed with FCS [18,19].	Yes [18,19]	-
**Small Insertion**	Exon 3	c.334dupG	p.Asp112Glyfs*36	Likely Pathogenic	Altered reading frame leads to premature truncation eliminating ~69.1% of peptide sequence.	Yes [61]	-
**Missense**	Exon 3	c.337T>C	p.Trp113Arg	Pathogenic	*In vitro* study in COS cells revealed that this variant results in near complete loss of enzymatic activity and LPL mass compared to wild-type, with minimal LPL mass and activity detected in both cell media and lysate [63].<br><br>Variant was first reported in compound heterozygosity in an FCS patient alongside the pathogenic nonsense variant LPL:c.397C>T:p.Gln133Term [63] and has also been reported in homozygous state in a separate FCS patient [64].<br><br>Another likely pathogenic missense variant LPL:c.337T>G:p.Trp113Gly has been observed impacting this codon, which provides additional indirect evidence for a deleterious impact of altering this codon.	Yes [12,29,52,63–65]	1540
**Missense**	Exon 3	c.337T>G	p.Trp113Gly	Likely Pathogenic	Functional data are unavailable.<br><br>Variant has been reported in a homozygous individual in a cohort of patients with familial lipoprotein lipase deficiency [66]. Exact lipid values, LPL mass, and LPL activity values were not reported for this patient.<br><br>Another pathogenic missense variant LPL:c.337T>C:p.Trp113Arg has been observed impacting	Yes [66]	-

					this codon, which provides additional indirect evidence for a deleterious impact of altering this codon.		
**Small Insertion**	Exon 3	c.338_339ins17	p.Trp113Term	Likely Pathogenic	Insertion produces a premature termination codon leading to truncation eliminating ~76.4% of peptide sequence.	Yes [8]	-
**Nonsense**	Exon 3	c.339G>A	p.Trp113Term	Pathogenic	Substitution produces a premature stop codon leading to elimination of ~76.4% of the peptide sequence	No (reported in ClinVar)	2085152
**Missense**	Exon 3	c.346C>G	p.Arg116Gly	Likely Pathogenic	Functional data are unavailable.<br><br>Variant was observed in double heterozygosity with a premature truncation variant in LMF1 (LMF1:p.Arg233Term) to cause FCS [67].<br><br>Three other likely pathogenic missense variants, LPL:c.346C>T:p.Arg116Trp, LPL:c.347G>A:p.Arg116Gln, and LPL:c.347G>C:p.Arg116Pro, have been observed impacting this codon, which gives additional indirect evidence for a deleterious impact of altering this codon.	Yes [67]	-
**Missense**	Exon 3	c.346C>T	p.Arg116Trp	Likely Pathogenic	Functional data are unavailable.<br><br>Variant was observed in a heterozygous patient with mild-to-moderate HTG [5].<br><br>Three other likely pathogenic missense variants, LPL:c.346C>G:p.Arg116Gly, LPL:c.347G>A:p.Arg116Gln, and LPL:c.347G>C:p.Arg116Pro, have been observed impacting this codon, which gives additional indirect evidence for a deleterious impact of altering this codon.	Yes [5]	-
**Missense**	Exon 3	c.347G>A	p.Arg116Gln	Likely Pathogenic	Functional data are unavailable.<br><br>Variant has been reported in a patient that is a compound heterozygote for this variant and the common *APOA5* polymorphism APOA5:c.-1131T>C [18]. This patient was reported with severe hypertriglyceridemia and pancreatitis during pregnancy.<br><br>Three other likely pathogenic missense variants, LPL:c.346C>G:p.Arg116Gly, LPL:c.346C>T:p.Arg116Trp, and LPL:c.347G>C:p.Arg116Pro, have been observed impacting this codon, which gives additional indirect evidence for a deleterious impact of altering this codon.	Yes [8,12,18,24]	1210341
**Missense**	Exon 3	c.347G>C	p.Arg116Pro	Likely Pathogenic	Functional data are unavailable.	Yes [68,69]	3382796

					Variant has been observed in compound heterozygosity with the likely pathogenic missense variant LPL:c.472T>G:p.Tyr158Asp to cause LPL deficiency in a neonate [68,69].<br><br>Three other likely pathogenic missense variants, LPL:c.346C>G:p.Arg116Gly, LPL:c.346C>T:p.Arg116Trp, and LPL:c.347G>A:p.Arg116Gln, have been observed impacting this codon, which gives indirect evidence for a deleterious impact of altering this codon.		
**Small Insertion**	Exon 3	c.348_349ins17	p.Ala117Serfs*61	Likely Pathogenic	Altered reading frame leads to premature truncation eliminating ~62.7% of peptide sequence	Yes [8]	-
**Nonsense**	Exon 3	c.352C>T	p.Gln118Term	Likely Pathogenic	Substitution produces a premature stop codon leading to elimination of ~75.4% of the peptide sequence.	Yes [70]	-
**Small Deletion**	Exon 3	c.355del	p.Glu119Serfs*53	Pathogenic	Altered reading frame leads to premature truncation eliminating ~64% of the peptide sequence.	No (reported in ClinVar)	1455355
**Small Insertion**	Exon 3	c.373dupG	p.Ala125Glyfs*23	Likely Pathogenic	Altered reading frame leads to premature truncation eliminating ~69.1% of peptide sequence.	Yes [45]	-
**Missense**	Exon 3	c.382A>G	p.Thr128Ala	Likely Pathogenic	*In vitro* study in COS-1 cells found this variant to be associated with decreased LPL mass and activity [55].<br><br>Variant has been observed in compound heterozygosity with the pathogenic missense variant LPL:c.829G>A:p.Asp277Asn in a FCS patient [55]. *In vivo* measurements of post-heparin plasma LPL confirmed the *in vitro* findings, with the patient having extremely low LPL mass and activity [55].	Yes [55]	-
**Small Indel**	Exon 3	c.384delCinsTGGGCT	p.Lys129Glyfs*45	Likely Pathogenic	Altered reading frame leads to premature truncation eliminating ~63.6% of peptide sequence.	Yes [52,71]	-
**Small Deletion**	Exon 3	c.386_389del	p.Lys129Argfs*42	Pathogenic	Altered reading frame leads to premature truncation eliminating ~64.2% of the peptide sequence.	No (reported in ClinVar)	3066297
**Small Deletion**	Exon 3	c.386_390delAACTG	p.Lys129Serfs*17	Likely Pathogenic	Altered reading frame leads to premature truncation eliminating ~69.5% of the peptide sequence.	Yes [5]	-
**Missense**	Exon 3	c.394G>A	p.Gly132Arg	Pathogenic	*In vitro* study in COS-1 cells found that this variant is normally expressed and synthesized but the synthesized protein is catalytically inactive LPL, with only 1% of the LPL protein synthesized being released from cells upon heparin administration versus the ~53% of LPL released from COS-1 cells expressing wild-type LPL upon heparin administration. [72].	Yes [72]	-

					Variant was observed in compound heterozygosity with the likely pathogenic missense variant LPL: c.693C>G:p.Asp231Glu to produce FCS in a Japanese male infant [72]. *In vivo* analysis of the patient's post-heparin plasma confirmed the *in vitro* results, failing to detect LPL activity and LPL mass was less than 2% of controls [72].<br><br>Another likely pathogenic missense variant, LPL:c.395G>A:p.Gly132Glu, has been observed impacting this codon, which provides additional indirect evidence of a deleterious impact of altering this codon.		
**Missense**	Exon 3	c.395G>A	p.Gly132Glu	Likely Pathogenic	Functional data are unavailable.<br><br>Variant was identified by us from a patient sequenced on LipidSeq panel [12]. Unfortunately, clinical data are unavailable to us regarding this patient.<br><br>Another pathogenic missense variant, LPL:c.394G>A:p,Gly132Arg, has been observed impacting this codon, providing additional indirect evidence for a deleterious impact of altering this codon.	Yes [12]	-
**Nonsense**	Exon 3	c.397C>T	p.Gln133Term	Pathogenic	Substitution produces a premature stop codon leading to elimination of ~72.2% of peptide sequence.	Yes [73]	1524
**Missense**	Exon 3	c.406G>C	p.Ala136Pro	Likely Pathogenic	Functional data are unavailable.<br><br>Variant has been observed in compound heterozygosity with the likely pathogenic missense variant LPL:c.829G>C:p.Asp277His in an FCS patient [74]. *In silico* analysis predicted that this variant causes the protein to fold improperly [74].	Yes [74]	-
**Nonsense**	Exon 3	c.422G>A	p.Trp141Term	Likely Pathogenic	Substitution produces a premature stop codon leading to elimination of ~70.5% of the peptide sequence.	No (reported in our clinic)	-
**Missense**	Exon 3	c.429G>T	p.Glu143Asp	Likely Pathogenic	Functional data are unavailable.<br><br>Variant is reported in compound heterozygosity with the likely pathogenic missense variant LPL:c.905G>C:p.Cys302Ser in a severe HTG patient [18].<br><br>Interestingly, the altered nucleotide is the final nucleotide of exon 3 and thus within the canonical donor splice site. Consequently, while 6 of 7 algorithms predictions utilized by the original authors find a benign or tolerated effect for this variant, the authors also found that *in silico* analyses	Yes [18]	-

					predicted a substantial decrease in the functionality of the donor splice site and the possible activation of an alternative donor site in intron 3, indicating that this variant is predicted to result in abnormal splicing [18].		
**Splicing**	Intron 3	c.429+1G>T	N/A	Likely Pathogenic	Alters the donor splice site of intron 3, which is predicted to result in defective pre-mRNA splicing.<br><br>Reported in compound heterozygosity with the pathogenic missense variant LPL:c.836T>G:p.Leu279Arg in a patient diagnosed with FCS [75]. *In vivo* analysis of LPL protein expression in the proband and their family via flow cytometry revealed that they all had extremely deficient LPL protein expression [75].	Yes [75]	-
**Gross Deletion**	N/A	NC_000008.10:g.(?_19810811)_(19811874_?)del	N/A	Likely Pathogenic	Eliminates exons 4-5.	No (reported in ClinVar)	1076886
**Small Deletion**	Exon 4	c.438delT	p.Phe146Leufs*26	Likely Pathogenic	Altered reading frame leads to premature truncation eliminating ~64% of the peptide sequence.	Yes [44]	-
**Small Deletion**	Exon 4	c.440_443delACTA	p.Asn147Thrfs*24	Pathogenic	Altered reading frame leads to premature truncation eliminating ~64.2% of the peptide sequence.	Yes [52,76]	2735125
**Missense**	Exon 4	c.461A>G	p.His154Arg	Likely Pathogenic	Functional data are unavailable.<br><br>Variant has been observed in compound heterozygosity with the likely pathogenic missense variant LPL:c.862G>A:p.Ala288Thr to cause LPL deficiency in an infant [77].	Yes [77]	-
**Missense**	Exon 4	c.464T>C	p.Leu155Pro	Likely Pathogenic	Functional data are unavailable.<br><br>Variant was observed in compound heterozygosity with the pathogenic missense variant LPL:c.644G>A:p.Gly215Glu to cause FCS [78].	Yes [78]	1878280
**Missense**	Exon 4	c.472T>G	p.Tyr158Asp	Likely Pathogenic	Functional data are unavailable.<br><br>Variant has been observed in compound heterozygosity with the likely pathogenic missense variant LPL:c.347G>C:p.Arg116Pro to cause LPL deficiency [68,69].	Yes [68,69]	-
**Nonsense**	Exon 4	c.474C>G	p.Tyr158Term	Pathogenic	Substitution produces a premature stop codon leading to elimination of ~66.9% of the peptide sequence.	No (reported in ClinVar)	1069128
**Missense**	Exon 4	c.476G>C	p.Ser159Thr	Likely Pathogenic	Functional data are unavailable.<br><br>Variant has been reported in two siblings diagnosed with FCS and found to be homozygous both for this variant and	Yes [79]	-

					for the common *LPL* polymorphism LPL:c.1421C>G:p.Ser474Term [79].<br><br>While *in vivo* analysis of plasma LPL mass revealed levels within the reference range for both pre- and post-heparin administration, the characteristic increase in plasma LPL post-heparin administration was absent in both siblings, indicating a defect in catalytic activity, which arises from the fact that this variant abolishes/alters a catalytic triad residue necessary for catalytic functionality [79].		
**Missense**	Exon 4	c.478C>T	p.Leu160Phe	Likely Pathogenic	Functional data are unavailable.<br><br>Variant was observed in homozygous neonate as the cause of their LPL deficiency [80].	Yes [80]	362410
**Missense**	Exon 4	c.482G>A	p.Gly161Glu	Likely Pathogenic	Functional data are unavailable. Variant has been observed in a homozygous individual with severe HTG and greatly reduced post-heparin LPL activity, noted to be characteristic of LPL deficiency [81]. This patient was also heterozygous for LPL:c.953A>G:p.Asn318Ser [81].	Yes [81]	-
**Small Deletion**	Exon 4	c.483delA	p.Ala162Profs*10	Likely Pathogenic	Altered reading frame leads to premature truncation eliminating ~64% of peptide sequence.	Yes [82]	-
**Missense**	Exon 4	c.488A>G	p.His163Arg	Likely Pathogenic	Functional data are unavailable.<br><br>Variant was reported in a FCS patient found to be a compound heterozygote for this variant and the pathogenic missense variant LPL:c.337T>C:p.Trp113Arg [29]. *In vivo* analysis of plasma LPL mass and activity revealed detectable levels of pre- and post-heparin LPL mass, but the post-heparin increase in plasma LPL mass was minimal, and LPL activity was nearly completely abolished, which together strongly suggest near complete loss of catalytic activity of LPL but at least one of the alleles produces LPL that is secreted [29].	Yes [12,29,52]	-
**Missense**	Exon 4	c.496G>A	p.Gly166Ser	Pathogenic	*In vitro* study in COS cells found that, compared to wild-type, this variant is normally secreted, with only slightly decreased protein level found both in cell medium and homogenate, but LPL activity was totally abolished [83].<br><br>The variant has been reported in multiple homozygous patients with FCS [62,83]. Interestingly though, examination of LPL mass in a patient revealed near zero detectable pre- and post-heparin LPL mass, contradicting part of the *in vitro* findings [83]. Two potential explanations for this discrepancy were offered by these authors: 1) poor	Yes [12,52,62,83]	-

					secretion of a non-functional enzyme due to impaired protein folding or, more likely, 2) due to protein instability with enhanced catabolism *in vivo* [83].		
**Missense**	Exon 4	c.506G>A	p.Gly169Glu	Pathogenic	Initial *in vitro* study in COS-7 cells found that this variant is synthesized at normal levels but produced protein has near completely abolished catalytic activity and has greatly impaired secretion [84]. Another *in vitro* study in COS-1 cells also found that this variant had defective secretion and further investigations by these authors determined that this was due to the variant peptide being missorted to lysosomes for intracellular degradation [85].<br><br>Variant has been observed in two siblings to cause FCS, with who were both observed to have greatly decreased LPL activity and the proband had greatly reduced but measurable LPL mass in post-heparin plasma, similar to the observed phenotype in *in vitro* investigations [84].	Yes [84,85]	1532
**Small Deletion**	Exon 4	c.538del	p.Thr180Leufs*27	Pathogenic	Altered reading frame leads to premature truncation eliminating ~56.6% of the peptide sequence.	No (reported in ClinVar)	2849043
**Missense**	Exon 4	c.541G>C	p.Gly181Arg	Likely Pathogenic	Functional data are unavailable.<br><br>This variant impacts the last nucleotide of exon 4 which forms part of the canonical donor splice site sequence. MES predicts alternative splicing consequence for this variant.<br><br>Variant was observed in compound heterozygosity with the pathogenic missense variant LPL:c.644G>A:p.Gly215Glu in two FCS patients who were siblings [86].<br><br>Three other pathogenic/likely pathogenic missense variants, LPL:c.541G>A:p.Gly181Ser, LPL:c.542G>A:p.Gly181Asp, and LPL:c.542G>T:p.Gly181Val, have been observed impacting this codon, which provides additional indirect evidence of a deleterious impact of altering this codon.	Yes [86]	1072728
**Missense**	Exon 4	c.541G>A	p.Gly181Ser	Pathogenic	*In vitro* study in COS-B cells found that this variant produces normally secreted but catalytically inactive LPL [87].<br><br>As this variant impacts the last nucleotide of exon 4 which forms part of the canonical donor splice site sequence, the splicing impact of this variant was also assessed and it was found that intron 4 was spliced normally [87].	Yes [52,87]	1456311

					Variant was found in a homozygous state in multiple related individuals diagnosed with FCS [87]. Plasma LPL activity was absent in all homozygous probands [87].<br><br>Three other pathogenic/likely pathogenic missense variants, LPL:c.541G>C:p.Gly181Arg, LPL:c.542G>A:p.Gly181Asp, and LPL:c.542G>T:p.Gly181Val, have been observed impacting this codon, which provides additional indirect evidence of a deleterious impact of altering this codon.		
**Splicing**	Intron 4	c.541+1G>A	N/A	Pathogenic	Abolishes donor splice site for intron 4, likely leading to defective pre-mRNA splicing.	No (reported in ClinVar)	1324675
**Splicing**	Intron 4	c.542-1G>A	N/A	Likely Pathogenic	Abolishes acceptor splice site of intron 4, likely leading to defective pre-mRNA splicing.	Yes [44]	-
**Missense**	Exon 5	c.542G>A	p.Gly181Asp	Likely Pathogenic	Functional data are unavailable.<br><br>Although this variant impacts the first nucleotide of exon 5 which forms part of the canonical acceptor splice site sequence, alternative splicing is not predicted as a consequence of this variant.<br><br>Variant has been reported as a pathogenic variant associated with hypertriglyceridemia in one study but phenotypic and allelic data are not included [17].<br><br>Three other pathogenic/likely pathogenic missense variants, LPL:c.541G>C:p.Gly181Arg, LPL:c.541G>A:p.Gly181Ser, and LPL:c.542G>T:p.Gly181Val, have been observed impacting this codon, which provides additional indirect evidence of a deleterious impact of altering this codon.	Yes [17]	-
**Missense**	Exon 5	c.542G>T	p.Gly181Val	Likely Pathogenic	*In vitro* study in COS-1 cells found that this variant produces catalytically inactive LPL that is not secreted from cells even upon heparin administration [88].<br><br>As this variant impacts the first nucleotide of exon 5, these authors also assessed if this variant impacts splicing by examining LPL mRNA extracted from monocytes of the proband's father, who was a carrier for this variant, and they found that intron 4 was spliced normally [88].<br><br>Variant was observed in compound heterozygosity alongside the likely pathogenic splicing variant	Yes [88,89]	-

					LPL:c.1322+2T>C to produce LPL deficiency in an infant [88].<br><br>Three other pathogenic/likely pathogenic missense variants, LPL:c.541G>C:p.Gly181Arg, LPL:c.541G>A:p.Gly181Ser, and LPL:c.542G>A:p.Gly181Asp, have been observed impacting this codon, which provides additional indirect evidence of a deleterious impact of altering this codon.		
**Missense**	Exon 5	c.547G>A	p.Asp183Asn	Pathogenic	*In vitro* study in COS-7 cells found that this variant is secreted normally but is essentially catalytically inactive [90].<br><br>Variant was observed in compound heterozygosity with the pathogenic missense variant LPL:c.727T>A:p.Cys243Ser in a patient diagnosed with LPL deficiency [90]. Patient had minimal post-heparin LPL mass and essentially zero activity [90]. Deleterious impact of this variant likely arises from the fact that this variant abolishes/alters a catalytic triad residue [90].<br><br>Two other pathogenic/likely pathogenic missense variants, LPL:c.547G>C:p.Asp183His and LPL:c.548A>G:p.Asp183Gly, have been observed impacting this codon, which provides additional indirect evidence of a deleterious impact of altering this codon.	Yes [90]	1066635
**Missense**	Exon 5	c.547G>C	p.Asp183His	Likely Pathogenic	Functional data are unavailable, but the molecular defect likely arises from the fact that this variant abolishes/alters a catalytic triad residue.<br><br>Variant was observed in a homozygous individual with LPL deficiency but phenotypic details are not available [54].<br><br>Two other pathogenic/likely pathogenic missense variants, LPL:c.547G>A:p.Asp183Asn and LPL:c.548A>G:p.Asp183Gly, have been observed impacting this codon, which provides additional indirect evidence of a deleterious impact of altering this codon.	Yes [12,54]	-
**Missense**	Exon 5	c.548A>G	p.Asp183Gly	Pathogenic	Two separate *in vitro* studies of this variant found that it resulted in reduced LPL mass and completely abolished LPL activity, most likely due to the fact that this variant abolishes/alters a catalytic triad residue [90,91].<br><br>Both of these studies also report homozygotes for these variants diagnosed with LPL deficiency [90,91]. Patients	Yes [52,90,91]	1533

					reported with this variant in one study had reduced post-heparin LPL mass and activity [90].<br><br>Two other pathogenic/likely pathogenic missense variants, LPL:c.547G>A:p.Asp183Asn and LPL:c.547G>C:p.Asp183His, have been observed impacting this codon, which provides additional indirect evidence of a deleterious impact of altering this codon.		
**Missense**	Exon 5	c.551C>G	p.Pro184Arg	Pathogenic	*In vitro* study in COS-1 cells found that this variant produces a catalytically inactive enzyme that is secreted at a slightly lower level than wild-type [92].<br><br>Variant was found in a homozygous proband with LPL deficiency, who was observed to have almost completely absent LPL activity and heparin administration only slightly increased plasma LPL mass [92].	Yes [52,92]	-
**Missense**	Exon 5	c.553G>A	p.Ala185Thr	Pathogenic	*In vitro* study found that this variant produced enzyme with extremely low but detectable LPL activity [66].<br><br>Multiple homozygous FCS patients have been found for this variant including in our clinic [12,66]. Analysis of plasma LPL mass and activity in one individual found increased LPL mass but low post-heparin activity [66].	Yes [12,66]	-
**Missense**	Exon 5	c.557G>A	p.Gly186Glu	Likely Pathogenic	Functional data are unavailable.<br><br>We have previously reported this variant in an FCS patient who was homozygous for this variant and was found to have undetectable LPL activity in post-heparin plasma [41]. It has also been reported in another homozygous individual with FCS [64] and in an infant with hyperchylomicronemia found to be compound heterozygous for this variant and the pathogenic missense variant LPL:c.662T>C:p.Ile221Thr [93].	Yes [41,64,93]	2735126
**Small Deletion**	Exon 5	c.566_567del	p.Phe189Term	Pathogenic	Deletion produces a premature stop codon leading to elimination of ~60.4% of the peptide sequence.	No (reported in ClinVar)	1401888
**Missense**	Exon 5	c.569A>G	p.Glu190Gly	Likely Pathogenic	Functional data are unavailable.<br><br>Variant has been observed in an FCS patient found to be compound heterozygous for this variant and the pathogenic missense variant LPL:c.644G>A:p.Gly215Glu [94]. This patient was observed to have completely absent post-heparin LPL activity [94].	Yes [94]	2735128

**Small Deletion**	Exon 5	c.572_588del	p.Tyr191Serfs*5	Pathogenic	Altered reading frame leads to premature truncation eliminating ~58.9% of the peptide sequence.	No (reported in ClinVar)	1427499
**Nonsense**	Exon 5	c.573T>G	p.Tyr191Term	Pathogenic	Substitution produces a premature stop codon leading to elimination of ~60% of the peptide sequence.	No (reported in ClinVar)	2814855
**Missense**	Exon 5	c.589C>T	p.Arg197Cys	Likely Pathogenic	Functional data are unavailable.<br><br>Three other pathogenic/likely pathogenic missense variants, LPL:c.590G>A:p.Arg197His, LPL:c.590G>T:p.Arg197Leu, and LPL:c.590G>C:p.Arg197Pro, have been reported affecting this codon, which provides additional indirect evidence for a deleterious impact of altering this codon.	No (reported in ClinVar)	1471786
**Missense**	Exon 5	c.590G>A	p.Arg197His	Pathogenic	*In vitro* study in HEK293T cells found that this variant significantly reduced the amount of LPL found in cell lysate (intracellular LPL mass) and cell medium (secreted LPL mass) compared to wild-type controls [95].<br><br>This variant has been reported in multiple studies in numerous individuals with severe HTG [18,61,81,95,96]. It has also been reported in two other studies without associated clinical nor allelic data [58,97].<br><br>At least two other pathogenic/likely pathogenic missense variants, LPL:c.590G>T:p.Arg197Leu, and LPL:c.590G>C:p.Arg197Pro, have been reported affecting this codon, which provides additional indirect evidence for a deleterious impact of altering this codon.	Yes [12,18,58,61,81,95–97]	521082
**Missense**	Exon 5	c.590G>T	p.Arg197Leu	Likely Pathogenic	Functional data are unavailable.<br><br>Variant was first reported in a patient diagnosed with FCS with non-detectable LPL mass nor activity [98]. This patient was a compound heterozygote for this variant and 3 other variants, 2 of which were synonymous variants not expected to have any effect [98]. The final variant detected was LPL:c:1421C>G:p.Ser474Term [98], a common gain-of-function variant in LPL [99]. This variant has also been reported in multiple other studies, including 2 homozygous individuals with severe HTG [18].<br><br>At least two other pathogenic/likely pathogenic missense variants, LPL:c.590G>A:p.Arg197His and LPL:c.590G>C:p.Arg197Pro, have been reported affecting	Yes [18,43,97, 98]	1452072

					this codon, which provides additional indirect evidence for a deleterious impact of altering this codon.		
**Missense**	Exon 5	c.590G>C	p.Arg197Pro	Likely Pathogenic	Functional data are unavailable.<br><br>We have observed this variant in a patient sequenced on LipidSeq from our clinic who was found to be homozygous for this variant and presented with severe HTG.<br><br>At least two other pathogenic/likely pathogenic missense variants, LPL:c.590G>A:p.Arg197His and LPL:c.590G>T:p.Arg197Leu, have been reported affecting this codon, which provides additional indirect evidence for a deleterious impact of altering this codon.	No (reported in our clinic)	-
**Missense**	Exon 5	c.596C>G	p.Ser199Cys	Pathogenic	*In vitro* study in COS-1 cells found that this variant leads to secreted LPL mass at roughly ~20% of wild-type level and LPL activity at ~5% of wild-type level [100]. Interestingly, intracellular variant LPL mass was greater than wild-type levels, suggesting that the major defect of this variant is impaired secretion and/or enhanced degradation of LPL specifically within the cell medium [100].<br><br>This variant was observed in a homozygous patient who developed LPL deficiency during her first pregnancy, leading to acute pancreatitis but also, seemingly, to the still birth of her first pregnancy [100]. She continued to have mild-to-moderate HTG afterwards, which progressed to severe HTG when she became pregnant again [100].	Yes [100]	1544
**Small Deletion**	Exon 5	c.596delC	p.Ser199Phefs*8	Pathogenic	Altered reading frame leads to premature truncation eliminating ~56.6% of the peptide sequence.<br><br>A novel and unique mechanism of disease has been reported in association with this variant [101]. Specifically, this variant was reported in a heterozygote with no other TG raising variants that was suffering from severe unmanageable fasting chylomicronemia with recurrent acute pancreatitis [101]. Given the lack of secondary factors contributing to dyslipidemia in this patient, the severity of this patient's condition could not be explained by heterozygous LPL deficiency from this variant alone [101]. It was discovered by Western blotting, that despite no personal history of autoimmune disease, with only family history being vitiligo in patient's father, this patient was expressing an antihuman LPL IgG and immunosuppressive	Yes [101]	-

					therapy was confirmed to reduce HTG severity in this patient [101].		
**Small Deletion**	Exon 5	c.599delC	p.Pro200Leufs*7	Likely Pathogenic	Altered reading frame leads to premature truncation eliminating ~56.6% of peptide sequence.	Yes [102]	-
**Missense**	Exon 5	c.602A>T	p.Asp201Val	Pathogenic	*In vitro* studies in CHO cells found that this variant is normally synthesized but is not secreted and has greatly reduced LPL activity, consistent with the authors' prediction that the substitution of Asp for Val at this position renders this residue unable to participate in the coordination of a Ca^{2+} ion into the structure of mature LPL, interfering with proper LPL folding [103].<br><br>Variant was discovered in two homozygous FCS probands descended from common ancestors in the Lebanese population [104]. Multiple heterozygous relatives of these probands presented with mild-to-moderate HTG as well, alongside one normolipidemic heterozygote [104].	Yes [103,104]	-
**Missense**	Exon 5	c.604G>A	p.Asp202Asn	Likely Pathogenic	Functional data are unavailable.<br><br>Variant was observed in compound heterozygosity with the likely pathogenic missense variant LPL:c.698A>G:p.Tyr233Cys to produce LPL deficiency [79]. Analysis of pre- and post-heparin LPL mass in this patient showed that both were greatly decreased compared to the wild-type reference range for these values [79]. In fact, in this patient pre- and post-heparin LPL mass were almost equal with no increase observed upon heparin administration, corresponding to LPL deficiency [79]. While direct functional evidence are unavailable, a later structural study of the LPL-GPIHBP1 complex found that Asp202 is involved in coordinating a Ca^{2+} ion into the mature LPL structure and that this residue is more sterically constrained than the adjacent Asp201 residue that is also involved in coordinating the Ca^{2+} ion [103]. *In vitro* study in CHO cells for a different variant altering this residue (LPL:p.Asp202Glu) was investigated and it was found that LPL:p.Asp202Glu variant peptide was not secreted and LPL activity was virtually completely abolished [103]. This result suggests that this variant likely produces a similar molecular defect.	Yes [79]	-
**Missense**	Exon 5	c.607G>A	p.Ala203Thr	Pathogenic	Two separate studies have investigated the molecular defect produced by this variant. The earliest *in vitro* study in COS-7 cells reported that LPL activity was essentially completely abolished despite increased pre- and post-	Yes [105,106]	1519

					heparin LPL mass compared to wild-type controls [105]. A second *in vitro* study in COS-1 cells also found that catalytic activity was abolished, and additionally observed that while intracellular variant LPL mass was similar to wild-type, very little secreted variant protein was observed [106]. Both of these *in vitro* studies also demonstrated that this variant reduces the affinity of the protein for heparin [105,106].<br><br>Variant has bene observed in homozygous state in an LPL-deficient patient [105].		
**Missense**	Exon 5	c.617T>C	p.Val206Ala	Likely Pathogenic	Functional data are unavailable.<br><br>The earliest report of this variant comes from a heterozygous patient with severe HTG who was found to have normal post-heparin LPL activity [81]. However, analysis of 3 related FCS patients (3 siblings) found to be homozygous for this variant found that post-heparin LPL activity and mass were both reduced greatly compared to wild-type [107].	Yes [12,81,107]	-
**Missense**	Exon 5	c.621C>G	p.Asp207Glu	Pathogenic	*In vitro* study in COS-7 cells demonstrated that this variant was normally synthesized and secreted but had completely abolished catalytic activity [108].<br><br>Variant was reported in two homozygous siblings with FCS and in heterozygous state in their parents who were mildly hypertriglyceridemic [108]. Both homozygous patients demonstrated greatly reduced post-heparin plasma LPL activity and mass [108].	Yes [108]	1547
**Missense**	Exon 5	c.622G>A	p.Val208Ile	Likely Pathogenic	*In vitro* experiment determined that this variant reduces specific activity of LPL and mildly decreases secretion and total (sum of cell medium and homogenate) LPL mass produced compared to wild type [56].<br><br>Variant was initially reported in a heterozygous proband with mild-to-moderate HTG, who was found to have normal LPL activity but decreased LPL mass *in vivo* [56].	Yes [12,56,59]	2412842
**Small Deletion**	Exon 5	c.624delC	p.Leu209Tyrfs*43	Likely Pathogenic	Altered reading frame leads to premature truncation eliminating ~47.2% of peptide sequence.	Yes [109]	-
**Nonsense**	Exon 5	c.626T>G	p.Leu209Term	Likely Pathogenic	Substitution produces a premature stop codon leading to elimination of ~56.2% of the peptide sequence.	No (reported in LOVD3)	-

**Missense**	Exon 5	c.628C>G	p.His210Asp	Likely Pathogenic	*In vitro* study in COS cells demonstrated that variant produces catalytically inactive protein that seems to be normally synthesized and secreted [26].<br><br>Variant has been reported in a Austrian FCS proband found to be compound heterozygous for this variant and the likely pathogenic small deletion variant LPL:c.89-4_89-2delCCA and in multiple relatives of this proband with HTG [26]. Compound heterozygous proband was found to have post-heparin plasma LPL activity close to zero while heterozygous carriers had reduced post-heparin plasma LPL activity ranging from 49 to 79% of wild-type controls [26].<br><br>Three other pathogenic/likely pathogenic missense variants, LPL:c.629A>G:p.His210Arg, LPL:c.629A>T:p.His210Leu, and LPL:c.630C>G:p.His210Gln, have been reported affecting this codon, which provides additional indirect evidence for a deleterious impact of altering this codon.	Yes [26]	-
**Missense**	Exon 5	c.629A>G	p.His210Arg	Likely Pathogenic	Functional data are unavailable.<br><br>Variant has been reported in a homozygote who was confirmed to be deficient in post-heparin LPL activity but had normal post-heparin LPL mass [62].<br><br>Three other pathogenic/likely pathogenic missense variants, LPL:c.628C>G:p.His210Asp, LPL:c.629A>T:p.His210Leu, and LPL:c.630C>G:p.His210Gln, have been reported affecting this codon, which provides additional indirect evidence for a deleterious impact of altering this codon.	Yes [12,62]	-
**Missense**	Exon 5	c.629A>T	p.His210Leu	Likely Pathogenic	*In vitro* study in HEK293T cells found that variant peptide had significantly greater expression than wild-type in cell lysate but had significantly reduced expression in cell medium, indicating that variant reduced secretion but did not impact peptide synthesis [110]. It was also observed that LPL activity was completely abolished in both cell lysate or medium after heparin treatment, indicating that this variant eliminates catalytic activity [110].<br><br>Variant was reported in a heterozygous patient with severe HTG and acute pancreatitis during pregnancy [110]. This patient was found to have post-heparin plasma LPL mass at approximately half the mean value for 10 normal controls and post-heparin LPL activity around 60% of the mean value in 10 normal controls [110].	Yes [110]	-

					Three other pathogenic/likely pathogenic missense variants, LPL:c.628C>G:p.His210Asp, LPL:c.629A>G:p.His210Arg, and LPL:c.630C>G:p.His210Gln, have been reported affecting this codon, which provides additional indirect evidence for a deleterious impact of altering this codon.		
**Missense**	Exon 5	c.630C>G	p.His210Gln	Likely Pathogenic	*In vitro* study determined that this variant produces a normally synthesized protein that has near completely abolished catalytic activity (~10% of wild-type) and undergoes impaired secretion (~25% LPL mass secreted compared to wild-type) [111].<br><br>Variant was observed in heterozygous state in a proband and their daughters, with the proband demonstrating variable and occasionally severe HTG, with a milder but still hypertriglyceridemic phenotype observed in the daughters [111].<br><br>Three other pathogenic/likely pathogenic missense variants, LPL:c.628C>G:p.His210Asp, LPL:c.629A>G:p.His210Arg, and LPL:c.629A>T:p.His210Leu, have been reported affecting this codon, which provides additional indirect evidence for a deleterious impact of altering this codon.	Yes [111]	-
**Missense**	Exon 5	c.634T>C	p.Phe212Leu	Likely Pathogenic	Functional data are unavailable.<br><br>Variant has been reported in an FCS patient found to be compound heterozygous for this variant and the pathogenic missense variant LPL:c.242G>A:p.Gly81Asp [24].	[12,24]	1425535
**Nonsense**	Exon 5	c.640A>T	p.Arg214Term	Pathogenic	Substitution produces a premature stop codon leading to elimination of ~55.2% of the peptide sequence.	No (reported in ClinVar)	948095
**Missense**	Exon 5	c.642A>C	p.Arg214Ser	Likely Pathogenic	Functional data are unavailable.<br><br>Variant is reported in an FCS proband that was found to be a compound heterozygote for this variant and a gross deletion (unknown breakpoints) of exons 3-7 of *LPL* [44].	Yes [44]	-
**Missense**	Exon 5	c.643G>A	p.Gly215Arg	Pathogenic	*In vitro* study in COS-1 cells determined that this variant produces protein that has mildly reduced secretion and nearly completely abolished catalytic activity (less than 1%) compared to wild-type [55].<br><br>Variant was reported in a homozygous proband and their brother, both diagnosed with FCS [55].	Yes [55]	-

					Another pathogenic missense variant, LPL:c.644G>A:p.Gly215Glu, has been reported affecting this codon, which provides additional indirect evidence for a deleterious impact of altering this codon.		
**Missense**	Exon 5	c.644G>A	p.Gly215Glu	Pathogenic	Multiple *in vitro* investigations in COS cells and 1 in HEK293T cells have shown that this variant produces a catalytically inactive enzyme [65,106,112,113]. It has also been determined that this variant impairs production of the variant peptide as well as it's secretion, but neither is completely abolished [65,106,112,113]. Interestingly, it has been demonstrated that the variant peptide produced has some reduced level of catalytic activity when rescued in its nascent state from cells, suggesting that the full loss of catalytic activity observed in other studies likely occurs due to decreased stability compared to wild-type of the variant peptide leading to rapid inactivation intracellularly and/or during secretion [106]. It has also been demonstrated that it is quite likely that glycine is the only amino acid at this residue that can adopt a confirmation that fulfills the constraints for proper folding of the protein [106].<br><br>This variant has been reported in many studies as a frequent cause of both FCS and MCS [12,13,17,20,21,24,48,50,52,59,62,64,65,106,112–126].<br><br>Another pathogenic missense variant, LPL:c.643G>A:p.Gly215Arg, has been reported affecting this codon, which provides additional indirect evidence for a deleterious impact of altering this codon.	Yes [12,13,17, 20,21,24, 48,50,52, 59,62,64, 65,106, 112–126]	1522
**Small Deletion**	Exon 5	c.651delT	p.Gly218Valfs*34	Likely Pathogenic	Altered reading frame leads to premature truncation eliminating ~47.2% of peptide sequence.	Yes [18,19]	-
**Nonsense**	Exon 5	c.655C>T	p.Arg219Term	Likely Pathogenic	Substitution produces a premature stop codon leading to elimination of ~54.1% of peptide sequence.	Yes [127]	-
**Missense**	Exon 5	c.658A>C	p.Ser220Arg	Pathogenic	*In vitro* study in COS-B cells determined that this variant has extremely low (2% of wild-type) but still detectable catalytic activity [66].<br><br>Variant was observed in an FCS patient who was found to be compound heterozygous for this variant and a 2 kb insertion variant in *LPL* [66].<br><br>Another likely pathogenic missense variant, LPL:c.659G>A:p.Ser220Asn, has been reported affecting	Yes [12,66]	-

					this codon, which provides additional indirect evidence for a deleterious impact of altering this codon.		
**Missense**	Exon 5	c.659G>A	p.Ser220Asn	Likely Pathogenic	Functional data are unavailable.<br><br>Variant has been reported in homozygous state in an FCS patient with no other variants associated with dyslipidemia [64].<br><br>Another pathogenic missense variant, LPL:c.658A>C:p.Ser220Arg, has been reported affecting this codon, which provides additional indirect evidence for a deleterious impact of altering this codon.	Yes [64]	1490467
**Missense**	Exon 5	c.662T>C	p.Ile221Thr	Pathogenic	Multiple *in vitro* studies have established that this variant produces catalytically inactive protein [128–130].<br><br>Variant has been reported in multiple studies as the cause of FCS [7,12,50,52,128–132].	Yes [7,12,50,52, 128–132]	1529
**Missense**	Exon 5	c.665G>A	p.Gly222Glu	Likely Pathogenic	*In vitro* study in COS-1 cells determined that this variant leads to reduced secreted LPL mass and completely abolished catalytic activity [106].<br><br>Variant was reported in a homozygote with FCS [106].	Yes [106]	1546
**Missense**	Exon 5	c.679G>T	p.Val227Phe	Likely Pathogenic	*In vitro* study in HEK293T cells found that variant LPL was synthesized at a rate 35-50% lower than wild-type, that secretion of variant peptide was near completely abolished, and catalytic activity was totally abolished [65].<br><br>Multiple FCS patients have been reported to be homozygous for this variant [65,133].<br><br>Two other likely pathogenic missense variants, LPL:c.680T>C:p.Val227Ala and LPL:c.680T>G:p.Val227Gly, has been reported affecting this codon, which provides additional indirect evidence for a deleterious impact of altering this codon.	Yes [65,133]	-
**Missense**	Exon 5	c.680T>C	p.Val227Ala	Likely Pathogenic	Functional data are unavailable.<br><br>Variant has been reported in a heterozygote presenting with severe HTG [43]. This variant has also been reported in a Type V dyslipidemia patient found to be heterozygous for this variant, but exact clinical details are unknown [102], in a Japanese patient for whom the original report is unavailable [134], and in an Indian MCS patient [43].	Yes [43,102, 134]	2441229

					Two other likely pathogenic missense variants, LPL:c.679G>T:p.Val227Phe and LPL:c.680T>G:p.Val227Gly has been reported affecting this codon, which provides additional indirect evidence for a deleterious impact of altering this codon.		
**Missense**	Exon 5	c.680T>G	p.Val227Gly	Likely Pathogenic	Functional data are unavailable.<br><br>Variant has been reported in a compound heterozygote for this variant and the pathogenic missense variant LPL:c.755T>C:p.Ile252Thr found to have severe HTG [102] and in a homozygote with FCS [135]. The compound heterozygote was observed to have near completely abolished LPL activity [102].<br><br>Two other likely pathogenic missense variants, LPL:c.679G>T:p.Val227Phe and LPL:c.680T>C:p.Val227Ala have been reported affecting this codon, which provides indirect evidence for a deleterious impact of altering this codon.	Yes [102,135]	1405688
**Missense**	Exon 5	c.686A>G	p.His229Arg	Likely Pathogenic	Functional data are unavailable.<br><br>Variant has been reported in an FCS patient found to be compound heterozygous for this variant and the pathogenic missense variant LPL:c.829G>A:p.Asp277Asn [18].<br><br>Another likely pathogenic missense variant, LPL:c.686A>T:p.His229Leu, has been observed affecting this codon, which provides additional indirect evidence for a deleterious impact of altering this codon.	Yes [18]	1303255
**Missense**	Exon 5	c.686A>T	p.His229Leu	Likely Pathogenic	Functional data are unavailable.<br><br>Variant was observed in 2 heterozygotes with severe HTG [136]. Both patients were also heterozygous for a benign *APOA5* variant [136]. Analysis of patient plasma revealed that they had LPL mass in plasma similar to wild-type controls [136].<br><br>Another likely pathogenic missense variant, LPL:c.686A>G:p.His229Arg, has been observed affecting this codon, which provides additional indirect evidence for a deleterious impact of altering this codon.	Yes [136]	-
**Small Deletion**	Exon 5	c.688del	p.Val230Leufs*22	Pathogenic	Altered reading frame leads to premature truncation eliminating ~47.2% of the peptide sequence.	No (reported in ClinVar)	1366216

**Missense**	Exon 5	c.691G>C	p.Asp231His	Likely Pathogenic	Functional data are unavailable.<br><br>We have observed this variant in a HTG patient from our clinic who was sequenced on LipidSeq and was found to be a homozygote for this variant.<br><br>Another likely pathogenic missense variant, LPL:c.693C>G:p.Asp231Glu, has been observed affecting this codon, which provides additional indirect evidence for a deleterious impact of altering this codon.	No (reported in our clinic)	-
**Missense**	Exon 5	c.693C>G	p.Asp231Glu	Likely Pathogenic	*In vitro* studies have determined that this variant produces a catalytically inactive protein that has near totally abolished secretion and mildly reduced intracellular LPL mass [42,72].<br><br>Both a homozygote [42] and a compound heterozygote, for this variant and the pathogenic missense variant LPL:c.394G>A:p.Gly132Arg, with FCS have been observed [42,72].	Yes [42,72]	1528
**Missense**	Exon 5	c.695T>G	p.Ile232Ser	Likely Pathogenic	Functional data are unavailable.<br><br>Variant has been identified in an FCS patient found to be homozygous for this variant [29]. Analysis of this patient's plasma revealed complete absence of LPL activity and complete absence of both pre- and post-heparin LPL mass [29].	Yes [29]	-
**Missense**	Exon 5	c.697T>G	p.Tyr233Asp	Likely Pathogenic	Functional data are unavailable.<br><br>Variant has been reported in a heterozygote with mild-to-moderate HTG [5].<br><br>Another likely pathogenic missense variant, LPL:c.698A>G:p.Tyr233Cys, has been observed affecting this codon, which provides additional indirect evidence for a deleterious impact of altering this codon.	Yes [5]	-
**Missense**	Exon 5	c.698A>G	p.Tyr233Cys	Likely Pathogenic	Functional data are unavailable.<br><br>Variant has been reported in an FCS patient found to be a compound heterozygote for this variant and the likely pathogenic missense variant LPL:c.604G>A:p.Asp202Asn [79]. Analysis of pre- and post-heparin LPL mass in this patient showed that both were greatly decreased compared to the wild-type reference range for these values [79]. Additionally, in this patient, pre- and post-heparin LPL	Yes [79]	-

					mass were almost equal with no increase observed upon heparin administration, corresponding to LPL deficiency [79].<br><br>Another likely pathogenic missense variant, LPL:c.697T>G:p.Tyr233Asp, has been observed affecting this codon, which provides additional indirect evidence for a deleterious impact of altering this codon		
**Missense**	Exon 5	c.701C>T	p.Pro234Leu	Pathogenic	*In vitro* studies found that this variant results in completely abolished protein that has impaired secretion but normal synthesis [137].<br><br>This variant has been reported in a large number of studies as underlying FCS and/or MCS [62,124,137–141].	Yes [12,24,62, 124,137–142]	1527
**Small Deletion**	Exon 5	c.708delA	p.Gly237Valfs*15	Likely Pathogenic	Altered reading frame leads to premature truncation eliminating ~47.2% of peptide sequence.	Yes [12,122, 143]	-
**Missense**	Exon 5	c.710G>A	p.Gly237Asp	Likely Pathogenic	Functional data are unavailable.<br><br>Variant has been reported in 2 homozygotes with severe HTG and 4 of their heterozygous relatives of which only 3 individuals were found to have very mildly elevated TGs [18].	Yes [18]	2735129
**Missense**	Exon 5	c.720G>T	p.Gln240His	Likely Pathogenic	Functional data are unavailable.<br><br>Variant has been reported in compound heterozygosity with the pathogenic missense variant LPL:c.644G>A:p.Gly215Glu to cause FCS [144].	Yes [144]	-
**Missense**	Exon 5	c.721C>T	p.Pro241Ser	Pathogenic	Functional data are unavailable.<br><br>This variant has been reported in a homozygote [21] and multiple compound heterozygotes with LPL deficiency [19–23].	Yes [19–23]	1236167
**Missense**	Exon 5	c.727T>A	p.Cys243Ser	Likely Pathogenic	*In vitro* study found that variant reduced secretion and completely abolished catalytic activity [90].<br>Variant has been reported in a compound heterozygote for this variant and the pathogenic missense variant LPL:c:547G>A:p.Asp183Asn with LPL deficiency [90].	Yes [52,90]	-
**Small Deletion**	Exon 5	c.742delG	p.Ala248Leufs*4	Pathogenic	Altered reading frame leads to premature truncation eliminating ~47.2% of peptide sequence.	Yes [135,145]	1538
**Missense**	Exon 5	c.755T>A	p.Ile252Asn	Likely Pathogenic	Functional data are unavailable.<br><br>Variant was reported in a heterozygote with severe HTG [146].	Yes [146]	-

**Missense**	Exon 5	c.755T>C	p.Ile252Thr	Pathogenic	*In vitro* study in COS-1 cells found that this variant reduced catalytic activity to ~10% of wild-type control level and LPL mass to ~80% the wild-type control level [147].<br><br>Multiple compound heterozygotes for this variant and the pathogenic missense variant LPL:c.809G>A:p.Arg270His presenting with LPL deficiency have been reported, including a patient from our clinic [12,52,147]. Another compound heterozygote for this variant and the likely pathogenic missense variant LPL:c.680T>G:p.Val227Gly has also been reported [102].<br><br>Another pathogenic missense variant, LPL:c.756T>G:p.Ile252Met, has been observed affecting this codon, which provides additional indirect evidence for a deleterious impact of altering this codon.	Yes [12,17,43,52,58,102,147]	1554
**Missense**	Exon 5	c.756T>G	p.Ile252Met	Pathogenic	*In vitro* study in HEK293T cells found that this variant reduces LPL activity by 40% compared to wild-type control, with no significant impact on protein synthesis nor secretion [148].<br><br>Variant was reported in a heterozygote with MCS [148].<br><br>Another pathogenic missense variant, LPL:c.755T>C:p.Ile252Thr, has been observed affecting this codon, which provides additional indirect evidence for a deleterious impact of altering this codon.	Yes [148]	3119788
**Small Deletion**	Exon 5	c.763_766del	p.Arg255Aspfs*8	Pathogenic	Altered reading frame leads to premature truncation eliminating ~44.8% of the peptide sequence.	No (reported in ClinVar)	1452004
**Small Deletion**	Exon 5	c.765_766delAG	p.Gly256Thrfs*26	Pathogenic	Altered reading frame leads to premature truncation eliminating ~40.8% of peptide sequence.	Yes [149]	995957
**Small Insertion**	Exon 5	c.767_768insTAAATATT	p.Leu257Lysfs*10	Likely Pathogenic	Altered reading frame leads to premature truncation eliminating ~44% of peptide sequence.	Yes [150]	-
**Missense**	Exon 5	c.775G>A	p.Asp259Asn	Likely Pathogenic	Functional data are unavailable.<br><br>This variant impacts the last nucleotide of exon 5, which forms part of the canonical donor splice site sequence. MES and SpliceAI predict alternative splicing consequence for this variant.<br><br>We have observed two heterozygous patients sequenced on LipidSeq for this variant in our clinic [12,13]. One presented with mild-to-moderate HTG and the other with	Yes [12,13]	2415139

					severe HTG. It should be noted that this variant impacts the final nucleotide of exon 5, which is within the canonical donor splice site motif. Both MES and SpliceAI strong predict that this variant results in alternative splicing.		
**Splicing**	Intron 5	c.775+1G>A	N/A	Likely Pathogenic	Disrupts donor splice site for intron 5, likely leading to defective pre-mRNA splicing.	No (reported in LOVD3)	-
**Nonsense**	Exon 6	c.784C>T	p.Gln262Term	Pathogenic	Substitution produces a premature stop codon leading to elimination of ~45.1% of the peptide sequence.	Yes [12,151]	523886
**Missense**	Exon 6	c.797G>A	p.Cys266Tyr	Likely Pathogenic	Functional data are unavailable.<br><br>Variant is only reported as a clinical testing result in a patient with FCS, but clinical data nor exact zygosity are included.	No (reported in ClinVar)	522719
**Nonsense**	Exon 6	c.798C>A	p.Cys266Term	Likely Pathogenic	Substitution produces a premature stop codon leading to elimination of ~44.2% of the peptide sequence.	Yes [152]	-
**Missense**	Exon 6	c.798C>G	p.Cys266Trp	Likely Pathogenic	Functional data are unavailable.<br><br>Variant was found in homozygous state in an FCS patient [153]. Analysis of post-heparin plasma of this patient could not detect LPL mass nor activity [153].	Yes [153]	1557
**Missense**	Exon 6	c.802C>T	p.His268Tyr	Likely Pathogenic	Functional data are unavailable, however the molecular defect associated with this variant likely arises from the fact that this variant alters a catalytic triad residue.<br><br>Two FCS cases for this variant have been reported: the first was a compound heterozygote for this variant and the pathogenic missense variant LPL:c.808C>G:p.Arg270Gly and the second was a homozygote for this variant [75,154]. Flow cytometry was used to analyze the LPL protein expression levels in the homozygous patient and their family, revealing that the homozygote had deficient LPL expression [75].<br><br>Another likely pathogenic missense variant, LPL:c.804C>A:p.His268Gln has been observed affecting this codon, providing additional indirect evidence for a deleterious impact of altering this codon.	Yes [75,154]	3251541
**Missense**	Exon 6	c.804C>A	p.His268Gln	Likely Pathogenic	Functional data are unavailable, however the molecular defect associated with this variant likely arises from the fact that this variant alters a catalytic triad residue.<br><br>Variant has been reported in a FCS patient found to be a compound heterozygote for this variant and the likely	Yes [12,21]	3251983

					pathogenic frameshift variant LPL:c.1373delC:p.Ala458Aspfs*6 [21]. We have also observed an HTG patient with this variant who was sequenced on LipidSeq [12] and was found to be a compound heterozygote for this variant and the pathogenic missense variant LPL:c.662T>C:p.Ile221Thr.<br><br>Another likely pathogenic missense variant, LPL:c.802C>T:p.His268Tyr, has been observed affecting this codon, providing additional indirect evidence for a deleterious impact of altering this codon.		
**Missense**	Exon 6	c.805G>A	p.Glu269Lys	Pathogenic	*In vitro* study in COS-7 cells found that this variant is synthesized and secreted at levels similar to wild-type, but the protein is catalytically inactive [6].<br><br>The variant has been reported in numerous compound heterozygous patients presenting with FCS or in heterozygous state in patients presenting with HTG [6,12,17,19,58,59,61,155,156].	Yes [6,12,19,58, 59,61,155, 156]	1066636
**Missense**	Exon 6	c.808C>T	p.Arg270Cys	Pathogenic	*In vitro* study in COS cells found that this variant reduced secreted LPL mass to 13% of wild-type level and completely abolished catalytic activity [157].<br><br>Numerous studies have reported FCS patients found to be homozygous or compound heterozygous (with other pathogenic LPL variants) for this variant [12,52,55,131,135,157].<br><br>Three other pathogenic/likely pathogenic missense variants, LPL:c.808C>G:p.Arg270Gly, LPL:c.809G>A:p.Arg270His, and LPL:c.809G>T:p.Arg270Leu, have been observed affecting this codon, providing additional indirect evidence for a deleterious impact of altering this codon.	Yes [12,52,55, 131,135, 157]	1548
**Missense**	Exon 6	c.808C>G	p.Arg270Gly	Pathogenic	*In vitro* study in *cld* cells found that this variant reduces LPL activity by ~50% in both cell lysate and media, as well has decreasing LPL secretion by ~50% [158].<br><br>Both homozygosity and compound heterozygosity (with other pathogenic *LPL* variants) for this variant have been reported underlying FCS in numerous studies, and heterozygotes with HTG have also been reported [19,154,158–161].	Yes [19,154,158 –161]	1500351

					Three other pathogenic/likely pathogenic missense variants, LPL:c.808C>T:p.Arg270Cys, LPL:c.809G>A:p.Arg270His, and LPL:c.809G>T:p.Arg270Leu, have been observed affecting this codon, providing additional indirect evidence for a deleterious impact of altering this variant.		
**Missense**	Exon 6	c.809G>A	p.Arg270His	Pathogenic	*In vitro* study in COS cells found that this variant did not affect secretion but did abolish catalytic activity and reduced LPL synthesis by ~50% compared to wild-type [113]. Another similar *in vitro* study found similar results, with the major different being that this earlier study also observed impaired secretion [42].<br><br>Multiple studies report homozygous and compound heterozygous (for other pathogenic *LPL* variants) FCS patients carrying this variant, as well as some heterozygous carriers [12,13,42,52,113,128,162,163].<br><br>Three other pathogenic/likely pathogenic missense variants, LPL:c.808C>T:p.Arg270Cys, LPL:c.808C>G:p.Arg270Gly, and LPL:c.809G>T:p.Arg270Leu, have been observed affecting this codon, providing additional indirect evidence for a deleterious impact of altering this variant.	Yes [12,13,42, 52,113,128, 162,163]	1530
**Missense**	Exon 6	c.809G>T	p.Arg270Leu	Likely Pathogenic	Functional data are unavailable.<br><br>The original report of this variant is not available online but is cited in a review article that briefly discusses this variant but no clinical nor functional data are provided [143].<br><br>Three other pathogenic/likely pathogenic missense variants, LPL:c.808C>T:p.Arg270Cys, LPL:c.808C>G:p.Arg270Gly, and LPL:c.809G>A:p.Arg270His, have been observed affecting this codon, providing additional indirect evidence for a deleterious impact of altering this variant.	Yes [143]	-
**Missense**	Exon 6	c.811T>A	p.Ser271Thr	Pathogenic	*In vitro* study in COS1 cells found that this variant completely abolished LPL catalytic activity [47]. A follow up study by the same group additionally found via *in vitro* experiments that this variant also greatly impairs secretion but has no effect on protein synthesis [106].<br><br>This variant has been reported in compound heterozygosity with the likely pathogenic splicing variant LPL:c.250-	Yes [47,52,106]	1525

					1G>A in an FCS patient [47] and has also been identified in a heterozygote in a severe HTG cohort [52].		
**Small Deletion**	Exon 6	c.811del	p.Ser271Profs*10	Pathogenic	Altered reading frame leads to premature truncation eliminating ~41.1% of the peptide sequence	No (reported in ClinVar)	1370928
**Missense**	Exon 6	c.818A>G	p.His273Arg	Pathogenic	*In vitro* study in COS-1 cells found that this variant completely abolishes LPL catalytic activity in both cell media and in cell lysate, reducing it to ~2% of the wild-type level [45].<br><br>This variant was reported in two compound heterozygotes with LPL deficiency: one patient carried the likely pathogenic small insertion variant LPL:c.373dupG:p.Ala125Glyfs*22 on one allele and this variant on the other, and the other patient carried the pathogenic missense variant LPL:c.701C>T:p.Pro234Leu on one allele and both this variant and the common LPL:c.953A>G:p.Asn318Ser on the other allele [45].	Yes [45]	2136643
**Small Deletion**	Exon 6	c.821del	p.Leu274Profs*7	Pathogenic	Altered reading frame leads to premature truncation eliminating ~41.1% of the peptide sequence.	No (reported in ClinVar)	2768165
**Missense**	Exon 6	c.829G>A	p.Asp277Asn	Pathogenic	*In vitro* study in COS cells found that this variant had minimal catalytic activity at ~5% of wild-type level, non-impaired secretion but reduced protein synthesis compared to wild-type [113]. Another *in vitro* study did not observe any defect related to LPL mass but did observe completely abolished catalytic activity [164].<br><br>Numerous studies have observed FCS patients found to be homozygous or compound heterozygous (with other pathogenic LPL variants) for this variant [12,52,62,113,164].<br><br>Another likely pathogenic missense variant, LPL:c.829G>C:p.Asp277His, has been observed affecting this codon, providing additional indirect evidence for a deleterious impact of altering this codon.	Yes [12,52,62, 113,142, 164]	1539
**Missense**	Exon 6	c.829G>C	p.Asp277His	Likely Pathogenic	Functional data are unavailable.<br><br>Variant was reported in a FCS patient found to be compound heterozygous for this variant and for the likely pathogenic missense variant LPL:c.406G>C:p.Ala136Pro [74]. *In silico* analysis predicted that this variant causes the protein to fold improperly [74].	Yes [74]	-

					Another pathogenic missense variant, LPL:c.829G>A:p.Asp277Asn, has been observed affecting this codon, providing additional indirect evidence for a deleterious impact of altering this codon.		
**Missense**	Exon 6	c.833C>G	p.Ser278Cys	Pathogenic	*In vitro* study found that this variant results in abolished catalytic activity [165].<br><br>Variant has been reported in an FCS patient found to be compound heterozygous for this variant and the pathogenic missense variant LPL:c.829G>A:p.Asp277Asn [165].<br><br>Another likely pathogenic missense variant, LPL:c.833C>T:p.Ser278Phe, has been observed affecting this codon, providing additional indirect evidence for a deleterious impact of altering this codon.	Yes [52,165]	-
**Missense**	Exon 6	c.833C>T	p.Ser278Phe	Likely Pathogenic	Functional and clinical data are unavailable.<br><br>Variant has been reported as being identified in Japanese FCS patients [2].<br><br>Another pathogenic missense variant, LPL:c.833C>T:p.Ser278Phe, has been observed affecting this codon, providing additional indirect evidence for a deleterious impact of altering this codon.	Yes [2]	-
**Missense**	Exon 6	c.835C>G	p.Leu279Val	Pathogenic	*In vitro* study in COS-7 cells found that this variant results in significantly reduced mRNA levels and LPL activity compared to wild-type [30].<br><br>Variant has been reported in numerous compound heterozygous and homozygous patients with FCS and other forms of severe HTG [8,11,12,17,30,40,52,57,61,159,166,167].<br><br>Another pathogenic missense variant, LPL:c.836T>G:p.Leu279Arg, has been observed affecting this codon, providing additional indirect evidence for a deleterious impact of altering this codon.	Yes [8,11,12,30, 40,52,57, 61,159,166, 167]	855588
**Small Deletion**	Exon 6	c.835_836delCT	p.Leu279Valfs*3	Likely Pathogenic	Altered reading frame leads to premature truncation eliminating ~40.8% of peptide sequence	Yes [12,18,19, 52,66]	-
**Missense**	Exon 6	c.836T>G	p.Leu279Arg	Pathogenic	*In vitro* study in COS-1 cells found that this variant reduced LPL mass to 32% of wild-type and completely abolished catalytic activity [168].	Yes [8,12,15,52, 58,69,75,	851236

					Numerous studies have reported this variant in homozygous and compound heterozygous individuals with FCS and other forms of severe HTG [8,12,15,17,52,58,69,75,163,166,168–170]. *In vivo* analysis of LPL protein expression in a compound heterozygous proband and their family, including their father who was heterozygous for this variant, via flow cytometry revealed that they all had extremely deficient LPL protein expression [75].<br><br>Another pathogenic missense variant, LPL:c.835C>G:p.Leu279Val, has been observed affecting this codon, providing additional indirect evidence for a deleterious impact of altering this codon.	163,166, 168–170]	
**Small Deletion**	Exon 6	c.840delG	p.Asn281Metfs*23	Likely Pathogenic	Altered reading frame leads to premature truncation eliminating ~36.2% of peptide sequence	Yes [1]	-
**Missense**	Exon 6	c.856A>G	p.Ser286Gly	Likely Pathogenic	Functional data are unavailable.<br><br>Variant has been reported in compound heterozygosity with the pathogenic missense variant LPL:c.644G>A:p.Gly215Glu [171] and in homozygosity [136] in FCS patients. It was found that plasma LPL mass in a homozygote for this variant is similar to wild-type control, suggesting that the deleterious effect of this variant is likely due to a functional defect [136].<br><br>Two other pathogenic/likely pathogenic missense variants, LPL:c.856A>C:p.Ser286Arg and LPL:c.858T>C:p.Ser286Arg, have been observed affecting this codon, providing additional indirect evidence for a deleterious impact of altering this codon.	Yes [136,171]	2735131
**Missense**	Exon 6	c.856A>C	p.Ser286Arg	Likely Pathogenic	Functional nor clinical data are available.<br><br>However, this variant produces the same amino acid change as an established pathogenic variant, LPL:c.858T>A:p.Ser286Arg, which gives evidence for a deleterious effect of this variant as well.<br><br>Two other pathogenic/likely pathogenic missense variants, LPL:c.856A>G:p.Ser286Gly and LPL:c.858T>C:p.Ser286Arg, have been observed affecting this codon, providing additional indirect evidence for a deleterious impact of altering this codon.	No (reported in LOVD3)	-

**Missense**	Exon 6	c.858T>A	p.Ser286Arg	Pathogenic	*In vitro* study in COS-B cells found that this variant near completely abolishes catalytic activity to less than 2% of wild-type and mildly decreases secreted LPL mass to 73% of wild-type [172].<br><br>Multiple FCS patients have been found to be homozygous for this variant [172,173].<br><br>At least one other likely pathogenic missense variants, LPL:c.856A>G:p.Ser286Gly, has been observed affecting this codon, providing additional indirect evidence for a deleterious impact of altering this codon.	Yes [172,173]	-
**Small Deletion**	Exon 6	c.858_861del	p.Ser286Argfs*17	Likely Pathogenic	Altered reading frame leads to premature truncation eliminating ~36.4% of the peptide sequence.	No (reported in LOVD3)	-
**Missense**	Exon 6	c.862G>A	p.Ala288Thr	Pathogenic	An initial *in vitro* study in COS-1 cells found that this variant mildly reduced secreted LPL mass to 67% of wild-type and activity of the secreted protein to 36% of wild-type [168]. However, a more recent *in vitro* study in both HEK293T cells and in COS7 cells consistently observed a milder effect, with LPL secretion reduced to ~80% of wild-type and a concomitant reduction in LPL activity to ~80% of wild-type [174].<br><br>This variant has been reported in compound heterozygosity with the pathogenic missense variant LPL:c.836T>G:p.Leu279Arg in a patient with pregnancy-induced chylomicronemia [168] and in compound heterozygosity with the likely pathogenic missense variant LPL:c.461A>G:p.His154Arg to produce FCS [77].<br><br>Another likely pathogenic missense variant, LPL:c.862G>C:p.Ala288Pro, has been observed affecting this codon, providing additional indirect evidence for a deleterious impact of altering this codon.	Yes [8,77,131, 168,174]	2735132
**Missense**	Exon 6	c.862G>C	p.Ala288Pro	Likely Pathogenic	Functional nor clinical data are available.<br><br>One submission in ClinVar reports this variant in FCHL but no further details are given.<br><br>Another pathogenic missense variant, LPL:c.862G>A:p.Ala288Thr, has been observed affecting this codon, providing indirect evidence for a deleterious impact of altering this codon.	No (reported in ClinVar)	1685931

**Missense**	Exon 6	c.865T>C	p.Tyr289His	Likely Pathogenic	*In vitro* study in HEK293 cells found that this variant does not impact LPL synthesis nor secretion but does completely abolish catalytic activity [175].<br><br>Variant has been observed in homozygous state in an FCS patient who was also homozygous for the common polymorphism LPL:c.106G>A:p.Asp36Asn [175]. Interestingly, the patient presented with no detectable LPL mass in both pre- and post-heparin plasma, in contrast to the normal LPL mass observed in the *in vitro* investigation, which may indicate that *in vivo* this variant undergoes enhanced degradation [175].	Yes [175]	-
**Nonsense**	Exon 6	c.867C>A	p.Tyr289Term	Pathogenic	Substitution produces a premature stop codon leading to elimination of ~39.4% of the peptide sequence.	Yes [176]	1803250
**Missense**	Exon 6	c.872G>A	p.Cys291Tyr	Likely Pathogenic	Functional data are unavailable.<br><br>Variant was reported in a homozygous individual diagnosed with FCS [176].	Yes [176]	-
**Nonsense**	Exon 6	c.873C>A	p.Cys291Term	Likely Pathogenic	Substitution produces a premature stop codon leading to elimination of ~38.9% of the peptide sequence.	Yes [2]	-
**Missense**	Exon 6	c.889T>C	p.Phe297Leu	Likely Pathogenic	Functional data are unavailable.<br><br>We have previously observed this variant in a patient sequenced on LipidSeq [12], who was found to be heterozygous for this variant with severe HTG.<br><br>This variant induces the same amino acid change as an established pathogenic variant, LPL:c.891T>G:p.Phe297Leu, which was found to result in catalytically inactive enzyme [177], which provide additional evidence for a deleterious impact of this variant.	Yes [12]	-
**Missense**	Exon 6	c.891T>G	p.Phe297Leu	Pathogenic	*In vitro* study in COS-1 cells found that this variant produces catalytically inactive enzyme that is normally synthesized [177]. Additionally, only 3% of the synthesized variant LPL was released from cells upon heparin treatment, with the remainder found in the cells which indicates a secretion defect [177].<br><br>Variant has been reported in an FCS patient found to be homozygous for this variant [177].<br><br>Another likely pathogenic missense variant, LPL:c.889T>C:p.Phe297Leu, has been observed affecting	Yes [177]	-

					this codon, providing additional indirect evidence for a deleterious impact of altering this codon.		
**Small Insertion**	Exon 6	c.891dup	p.Glu298Term	Likely Pathogenic	Altered reading frame leads to premature truncation eliminating ~37.5% of the peptide sequence.	No (reported in LOVD3)	-
**Gross Insertion**	Exon 6/Intron 6	c.898_1018+1939 dup	N/A	Pathogenic	Partial exon 6 and intron 6 duplication.	Yes [178,179]	1765322
**Small Insertion**	Exon 6	c.899_917dup	p.Lys307Alafs*6	Likely Pathogenic	Altered reading frame leads to premature truncation eliminating ~34.3% of the peptide sequence.	No (reported in LOVD3)	-
**Gross Insertion**	Exon 6	c.899_921dup	p.Asn308Glyfs*4	Pathogenic	Altered reading frame leads to premature truncation eliminating ~34.5% of the peptide sequence.	Yes [158,159]	2484764
**Small Deletion**	Exon 6	c.901delC	p.Leu301Serfs*3	Likely Pathogenic	Altered reading frame leads to premature truncation eliminating ~36.2% of peptide sequence	Yes [81]	-
**Missense**	Exon 6	c.904T>C	p.Cys302Arg	Likely Pathogenic	Functional data are unavailable.<br><br>Variant has been found in an FCS patient found to be homozygous for this variant [180].<br><br>Two other likely pathogenic missense variants, LPL:c.905G>C:p.Cys302Ser and LPL:c.905G>T:p.Cys302Phe, have been observed affecting this codon, providing additional indirect evidence for a deleterious impact of altering this codon.	Yes [180]	424622
**Missense**	Exon 6	c.905G>C	p.Cys302Ser	Likely Pathogenic	Functional data are unavailable.<br><br>Variant has been reported in an FCS patient found to be a compound heterozygote for this variant and for the likely pathogenic missense variant LPL:c.429G>T:p.Gly143Asp [18].<br><br>Two other likely pathogenic missense variants, LPL:c.904T>C:p.Cys302Arg and LPL:c.905G>T:p.Cys302Phe, have been observed affecting this codon, providing additional indirect evidence for a deleterious impact of altering this codon.	Yes [18]	-
**Missense**	Exon 6	c.905G>T	p.Cys302Phe	Likely Pathogenic	Functional data are unavailable.<br><br>Variant has been observed in our clinic [12,13,181] in 3 heterozygotes, 2 of which had HTG.<br><br>Two other likely pathogenic missense variants, LPL:c.904T>C:p.Cys302Arg and	Yes [12,13,181]	-

					LPL:c.905G>C:p.Cys302Ser, have been observed affecting this codon, providing indirect evidence for a deleterious impact of altering this codon.		
**Missense**	Exon 6	c.909G>C	p.Leu303Phe	Likely Pathogenic	*In vitro* study in COS-7 cells found that this variant lead to a 60% reduction in protein synthesis, with completely abolished secretion and activity [182].<br><br>Variant was observed in homozygosity in an FCS patient who was observed to have greatly reduced LPL activity and mass in their post-heparin plasma [182].	Yes [182]	-
**Missense**	Exon 6	c.913T>C	p.Cys305Arg	Likely Pathogenic	Exact functional data are unavailable: To the best of our knowledge, this variant was first reported on a poster at the 11th International Symposium on Atherosclerosis in Paris held between October 5-9, 1997 but only an abstract is available online [183]. According to the abstract, *in vitro* study in COS-1 cells found abnormal LPL expression but further details are unavailable [183].<br><br>The variant has also been reported in an LPL deficient compound heterozygote for this variant and the likely pathogenic missense variant LPL:c.214A>G:p.Ser72Gly [184].<br><br>Two other pathogenic/likely pathogenic missense variants, LPL:c.914G>A:p.Cys305Tyr and LPL:c.914G>C:p.Cys305Ser, have been observed affecting this codon, providing additional indirect evidence for a deleterious impact of altering this codon.	Yes [134,184]	3366687
**Missense**	Exon 6	c.914G>A	p.Cys305Tyr	Likely Pathogenic	Functional data are unavailable.<br><br>A heterozygote for this variant displaying mild-to-moderate HTG has been reported [5].<br><br>Two other pathogenic/likely pathogenic missense variants, LPL:c.913T>C:p.Cys305Arg and LPL:c.914G>C:p.Cys305Ser, have been observed affecting this codon, providing additional indirect evidence for a deleterious impact of altering this codon.	Yes [5]	-
**Missense**	Exon 6	c.914G>C	p.Cys305Ser	Pathogenic	Functional data are unavailable.<br><br>Two other pathogenic/likely pathogenic missense variants, LPL:c.913T>C:p.Cys305Arg and LPL:c.914G>A:p.Cys305Tyr, have been observed affecting	No (reported in ClinVar)	1765969

					this codon, providing additional indirect evidence for a deleterious impact of altering this codon.		
**Nonsense**	Exon 6	c.919A>T	p.Lys307Term	Pathogenic	Substitution produces a premature stop codon leading to elimination of ~35.6% of the peptide sequence.	No (reported in ClinVar)	1458039
**Small Insertion**	Exon 6	c.924_925insG	p.Arg309Alafs*8	Likely Pathogenic	Altered reading frame leads to premature truncation eliminating ~33.5% of the peptide sequence.	No (reported in LOVD3)	-
**Missense**	Exon 6	c.925C>T	p.Arg309Cys	Likely Pathogenic	Functional data are unavailable.<br><br>We have previously found this variant in our clinic [12] in two 2 HTG patients both found to be heterozygous for this variant.	Yes [12]	2503904
**Small Deletion**	Exon 6	c.925del	p.Arg309Alafs*22	Pathogenic	Altered reading frame leads to premature truncation eliminating ~30.5% of the peptide sequence	No (reported in ClinVar)	2429741
**Missense**	Exon 6	c.928T>C	p.Cys310Arg	Pathogenic	*In vitro* study in COS-1 cells found that this variant led to significantly lower LPL activity and mass in both cell lysates and medium, indicating that the variant has impaired synthesis and/or enhanced degradation as well as impaired catalytic activity [185]. *In vivo* study utilizing knockin mice found that mice heterozygous for this variant demonstrated plasma LPL mass and activity ~48% lower than wild-type mice [186]. Lower expression of variant LPL was also observed in the skeletal muscle of knockin heterozygous mice when compared to wild-type mice [186].<br><br>The variant has been observed in a heterozygous proband and a compound heterozygous (for this variant and the likely pathogenic missense variant LPL:c.1187A>T:p.Glu396Val) family member both of whom presented with severe HTG and recurrent pancreatitis [185].<br><br>Another pathogenic missense variant, LPL:c.929G>A:p.Cys310Tyr, has been observed affecting this codon, providing additional indirect evidence of a deleterious effect of altering this codon.	Yes [185–187]	226449
**Missense**	Exon 6	c.929G>A	p.Cys310Tyr	Pathogenic	*In vitro* study in COS-1 cells found that this variant produced enzyme with completely abolished catalytic activity that was not secreted [56].<br><br>Variant was observed in an heterozygous proband with severe HTG [56].	Yes [17,56,123]	979021

					Another pathogenic missense variant, LPL:c.928T>C:p.Cys310Arg, has been observed affecting this codon, providing additional indirect evidence of a deleterious effect of altering this codon.		
**Small Deletion**	Exon 6	c.932del	p.Asn311Thrfs*20	Pathogenic	Altered reading frame leads to premature truncation eliminating ~30.5% of the peptide sequence	No (reported in ClinVar)	1418720
**Missense**	Exon 6	c.938T>C	p.Leu313Pro	Pathogenic	*In vitro* study found that this variant produced enzyme with near completely abolished LPL activity but normal mass [55].<br><br>Variant was observed in an FCS patient found to be compound heterozygous for this variant and the likely pathogenic small indel variant LPL:c.290_293delCCGCinsGG [55].	Yes [55]	2136644
**Missense**	Exon 6	c.940G>A	p.Gly314Ser	Pathogenic	*In vitro* study in COS-7 cell found that this variant is not secreted and had significantly reduced catalytic activity in cell lysate (~50% reduction compared to wild-type) and completely undetectable activity in cell media [61].<br><br>Variant was observed in an FCS patient found to be compound heterozygous for this variant and the pathogenic missense variant LPL:c.590G>A:p.Arg197His [61].	Yes [61]	-
**Small Deletion**	Exon 6	c.942del	p.Tyr315Metfs*16	Pathogenic	Altered reading frame leads to premature truncation eliminating ~30.5% of the peptide sequence	No (reported in ClinVar)	2026484
**Nonsense**	Exon 6	c.945T>A	p.Tyr315Term	Likely Pathogenic	Substitution produces a premature stop codon leading to elimination of ~33.9% of peptide sequence.	Yes [188]	-
**Small Deletion**	Exon 6	c.953delA	p.Asn318Ilefs*13	Likely Pathogenic	Altered reading frame leads to premature truncation eliminating ~30.5% of peptide sequence.	Yes [189]	-
**Small Deletion**	Exon 6	c.973_974del	p.Ser325Glnfs*28	Likely Pathogenic	Altered reading frame leads to premature truncation eliminating ~25.9% of the peptide sequence.	No (reported in LOVD3)	-
**Missense**	Exon 6	c.975C>G	p.Ser325Arg	Likely Pathogenic	*In vitro* study in COS-1 cells found that this variant only very mildly reduces catalytic activity compared to wild-type and had mildly reduced secretion compared to wild-type [56].<br><br>Variant was initially reported in a heterozygous proband with mild-to-moderate HTG who was found to have reduced LPL activity and mass in post-heparin plasma compared to controls [56]. We have also observed this	Yes [12,56]	2502783

					variant in our clinical testing [12] where it was identified in an HTG patient.		
**Missense**	Exon 6	c.983T>C	p.Met328Thr	Likely Pathogenic	Functional data are unavailable.<br><br>Variant was first observed in a heterozygote with severe HTG [66]. Variant has also been reported in 3 related patients with severe HTG all of whom were found to be homozygous for the variant [136]. Analysis of patient plasma found that all 3 of these patients had LPL mass similar to wild-type [136].<br><br>Two other likely pathogenic missense variants, LPL:c.983T>G:p.Met328Arg and LPL:c.984G>T:p.Met328Ile, have been observed impacting this variant, providing additional indirect evidence for a deleterious effect of altering this codon.	Yes [66,136]	-
**Missense**	Exon 6	c.983T>G	p.Met328Arg	Likely Pathogenic	Functional data are unavailable.<br><br>Variant was observed in compound heterozygosity with the pathogenic missense variant LPL:c.644G>A:p.Gly215Glu in an FCS patient [190]. Analysis of pre- and post-heparin plasma revealed that this patient had near completely abolished LPL mass with no increase observed upon heparin administration [190].<br><br>Two other likely pathogenic missense variants, LPL:c.983T>C:p.Met328Thr and LPL:c.984G>T:p.Met328Ile, have been observed impacting this variant, providing additional indirect evidence for a deleterious effect of altering this codon.	Yes [190]	-
**Missense**	Exon 6	c.984G>T	p.Met328Ile	Likely Pathogenic	Functional data are unavailable.<br><br>Variant has been observed in numerous studies. It was first observed in heterozygote with severe HTG [5]. Variant was also observed in 2 homozygotes and a compound heterozygote for this variant and the pathogenic missense variant LPL:c.644G>A:p.Gly215Glu, all found to have severe HTG [18]. Another compound heterozygote for this variant and the pathogenic missense variant LPL:c.644G>A:p.Gly215Glu was identified [120]. Finally, 4 FCS patients homozygous for this variant have been observed [191].	Yes [5,18,120, 191]	1685932

					Two other likely pathogenic missense variants, LPL:c.983T>C:p.Met328Thr and LPL:c.983T>G:p.Met328Arg, have been observed impacting this variant, providing additional indirect evidence for a deleterious effect of altering this codon.		
**Missense**	Exon 6	c.986A>C	p.Tyr329Ser	Likely Pathogenic	*In vitro* study in LO2 and TPC-1 cells found that this variant does not affect protein synthesis, but does significantly reduce LPL activity [192].<br><br>Variant was observed in a heterozygous proband with severe HTG and in their relatives [192].	Yes [192]	-
**Nonsense**	Exon 6	c.987C>A	p.Tyr329Term	Pathogenic	Substitution produces a premature stop codon leading to elimination of ~30.9% of peptide sequence.	Yes [12,19,151, 193]	1556
**Missense**	Exon 6	c.989T>C	p.Leu330Pro	Likely Pathogenic	Functional data are unavailable.<br><br>Variant was first reported in an FCS patient found to be homozygous for this variant [66]. We have also observed this variant in our clinic [12] in an FCS patient found to be homozygous for this variant.	Yes [12,66]	-
**Missense**	Exon 6	c.991A>G	p.Lys331Glu	Likely Pathogenic	Functional data are unavailable.<br><br>Variant has been reported in a homozygous infant who with failure to thrive, hepatosplenomegaly, and hyperlipidemia [194].	Yes [194]	1030690
**Missense**	Exon 6	c.998G>A	p.Arg333His	Pathogenic	*In vitro* study in COS cells found that this variant produces enzyme with near completely abolished post heparin activity in both cell medium and lysate [45].<br><br>The variant was first reported in a compound heterozygote for this variant and the pathogenic missense variant LPL:c.644G>A:p.Gly215Glu presenting with FCS [45]. It has also been reported in a heterozygote presenting with severe HTG [18].	Yes [18,45]	632073
**Nonsense**	Exon 6	c.1003C>T	p.Gln335Term	Pathogenic	Substitution produces a premature stop codon leading to elimination of ~29.5% of the peptide sequence	No (reported in ClinVar)	1076739
**Small Deletion**	Exon 6	c.1008delG	p.Met336Ilefs*10	Likely Pathogenic	Altered reading frame leads to premature truncation eliminating ~27.4% of peptide sequence	Yes [109]	-
**Nonsense**	Exon 6	c.1014C>A	p.Tyr338Term	Pathogenic	Substitution produces a premature stop codon leading to elimination of ~29.1% of peptide sequence.	Yes [12]	2582665
**Nonsense**	Exon 6	c.1014C>G	p.Tyr338Term	Pathogenic	Substitution produces a premature stop codon leading to elimination of ~29.1% of the peptide sequence.	Yes [12]	2972813

**Small Insertion**	Exon 6	c.1016_1017insC	p.Lys339Asnfs*15	Likely Pathogenic	Altered reading frame leads to premature truncation eliminating ~25.7% of peptide sequence.	Yes [195]	-
**Splicing**	Intron 6	c.1018+1G>A	N/A	Likely Pathogenic	Abolishes splice donor site of intron 6, likely leading to defective pre-mRNA splicing.	Yes [196]	-
**Splicing**	Intron 6	c.1019-3C>A	N/A	Likely Pathogenic	Abolishes the acceptor splice site of intron 6, and it has been shown that this variant leads to aberrant splicing producing a major transcript with exons 6 to 9 eliminated and also at a much lower rate a minor transcript with exon 7 eliminated [197]. Further analysis showed that the major transcript eliminating exons 6 to 9 was expressed at ~3% of wild-type levels [197].<br><br>The variant was observed in two FCS patients found to be homozygous for this variant [197] and in a FCS patient found to be a compound heterozygote for this variant and the pathogenic missense variant LPL:c.644G>A:p.Gly215Glu [64].	Yes [64,197]	1457932
**Splicing**	Intron 6	c.1019-3C>G	N/A	Likely Pathogenic	Abolishes the acceptor splice site of intron 6, likely leading to defective pre-mRNA splicing.<br><br>Variant has been reported in FCS patient found to be a compound heterozygote for this variant and the pathogenic missense variant LPL:c.808C>G:p.Arg270Gly [160].	Yes [160]	-
**Splicing**	Intron 6	c.1019-2A>T	N/A	Pathogenic	Abolishes the acceptor splice site of intron 6, and was shown in *in vitro* study in COS-1 cells to lead to aberrant splicing in which exon 7 is skipped, leading to a prematurely truncated protein [18].<br><br>Variant has been observed in multiple FCS patients found to be homozygous for this variant [18,19].	Yes [12,18,19]	1693264
**Splicing**	Intron 6	c.1019-1G>A	N/A	Pathogenic	Abolishes the acceptor splice site of intron 6, likely leading to defective pre-mRNA splicing.	No (reported in ClinVar)	2992733
**Small Deletion**	Exon 7	c.1044_1050delTTTTTCT	p.His348Glnfs*43	Likely Pathogenic	Altered reading frame leads to premature truncation eliminating ~17.9% of peptide sequence.	Yes [81]	-
**Missense**	Exon 7	c.1051G>A	p.Gly351Arg	Likely Pathogenic	Functional data are unavailable.<br><br>Variant has been reported in an FCS patient found to be homozygous for this variant [20].	Yes [12,20]	985671
**Missense**	Exon 7	c.1081G>A	p.Ala361Thr	Likely Pathogenic	Functional data are unavailable.<br><br>Variant has been observed in multiple FCS patients found to be homozygous for this variant [131,135,198]. Multiple of	Yes [131,135, 198]	1543

					these patients have demonstrated reduced post-heparin plasma LPL mass compared to wild-type [135,198].		
**Small Deletion**	Exon 7	c.1127_1137del	p.Ile376Thrfs*3	Pathogenic	Altered reading frame leads to premature truncation eliminating ~20.4% of the peptide sequence	No (reported in ClinVar)	3068354
**Small Deletion**	Exon 7	c.1138_1139delCT	p.Leu380Alafs*2	Likely Pathogenic	Altered reading frame leads to premature truncation eliminating ~19.8% of peptide sequence	Yes [52,143]	-
**Splicing**	Intron 7	c.1139+1G>A	N/A	Pathogenic	Variant impacts the splice donor site of intron 7, likely abolishing its functionality, leading to aberrant splicing.<br><br>Variant has only been reported in large, population scale studies and/or large multi-center cohort studies with no accompanying specific clinical data [17,151].	Yes [151]	973587
**Splicing**	Intron 7	c.1139+7A>G	N/A	Likely Pathogenic	Functional data are unavailable.<br><br>Variant has been reported in homozygous state in a FCS patients [24]. This patient had greatly reduced LPL activity in both pre- and post-heparin plasma [24].	Yes [24]	1392632
**Gross Deletion**	N/A	NC_000008.10:g.(?_19818402)_(19824563_?)del	N/A	Pathogenic	Eliminates exon 8-10.	Yes [199]	2422620
**Small Insertion**	Exon 8	c.1158dup	p.Lys387Term	Pathogenic	Altered reading frame leads to premature truncation eliminating ~18.7% of the peptide sequence.	No (reported in ClinVar)	2795633
**Small Insertion**	Exon 8	c.1160_1161insT	p.Lys387Asnfs*26	Pathogenic	Altered reading frame leads to premature truncation eliminating ~13.3% of peptide sequence.	Yes [151]	973588
**Small Insertion**	Exon 8	c.1163_1164insA	p.Tyr389Leufs*24	Likely Pathogenic	Altered reading frame leads to premature truncation eliminating ~13.3% of peptide sequence.	Yes [195]	-
**Nonsense**	Exon 8	c.1167C>A	p.Tyr389Term	Likely Pathogenic	Substitution produces a premature stop codon leading to elimination of ~18.3% of the peptide sequence.	No (reported in LOVD3)	-
**Small Deletion**	Exon 8	c.1172_1175del	p.Ser390_Phe391insTerm	Pathogenic	Altered reading frame leads to premature truncation eliminating ~17.9% of the peptide sequence	No (reported in ClinVar)	1075531
**Missense**	Exon 8	c.1174C>G	p.Leu392Val	Likely Pathogenic	Functional data are unavailable.<br><br>Variant has been reported in two siblings with FCS who were both found to be homozygous for this variant [200].	Yes [200]	1551
**Missense**	Exon 8	c.1187A>T	p.Glu396Val	Likely Pathogenic	*In vitro* study in COS-1 cells found that this variant likely impairs enzyme secretion from cells as it was found to be undetectable in cell medium but was found in cell lysate at levels comparable to wild-type [185].	Yes [185,201]	226450

					Variant has been reported in an LPL deficient proband who was found to be compound heterozygous for this variant and the pathogenic missense variant LPL:c.928T>C:p.Cys310Arg [185].		
**Small Deletion**	Exon 8	c.1187_1188del	p.Glu396Glyfs*16	Pathogenic	Altered reading frame leads to premature truncation eliminating ~13.5% of the peptide sequence	No (reported in ClinVar)	2840731
**Missense**	Exon 8	c.1211T>G	p.Met404Arg	Likely Pathogenic	*In vitro* study in HEK293 cells found that this variant led to near completely abolished protein synthesis, 80% reduction in protein secretion compared to wild-type, completely abolished catalytic activity [149].<br><br>Variant was observed in an FCS patient found to be homozygous for this variant [149]. Analysis of pre- and post-heparin plasma of this patient found near completely lost catalytic activity and extremely low level LPL protein mass [149].	Yes [149]	-
**Nonsense**	Exon 8	c.1216A>T	p.Lys406Term	Pathogenic	Substitution produces a premature stop codon leading to elimination of ~14.7% of the peptide sequence	No (reported in ClinVar)	1030688
**Nonsense**	Exon 8	c.1226G>A	p.Trp409Term	Likely Pathogenic	Substitution produces a premature stop codon leading to elimination of ~14.1% of the peptide sequence.	Yes [168]	-
**Nonsense**	Exon 8	c.1227G>A	p.Trp409Term	Pathogenic	Substitution produces a premature stop codon leading to elimination of ~14.1% of the peptide sequence.	Yes [42,132, 202]	1537
**Small Deletion**	Exon 8	c.1227delG	p.Trp409Term	Likely Pathogenic	Altered reading frame leads to premature truncation eliminating ~14.1% of peptide sequence.	Yes [134]	-
**Nonsense**	Exon 8	c.1250G>A	p.Trp417Term	Pathogenic	Substitution produces a premature stop codon leading to elimination of ~12.4% of the peptide sequence	No (reported in ClinVar)	1677766
**Nonsense**	Exon 8	c.1259G>A	p.Trp420Term	Pathogenic	Substitution produces a premature stop codon leading to elimination of ~11.8% of the peptide sequence.	No (reported in ClinVar)	1448765
**Nonsense**	Exon 8	c.1260G>A	p.Trp420Term	Likely Pathogenic	Substitution produces a premature stop codon leading to elimination of ~11.8% of the peptide sequence.	Yes [18]	-
**Nonsense**	Exon 8	c.1262G>A	p.Trp421Term	Likely Pathogenic	Substitution produces a premature stop codon leading to elimination of ~11.6% of the peptide sequence.	Yes [163,203]	-
**Nonsense**	Exon 8	c.1263G>A	p.Trp421Term	Pathogenic	Substitution produces a premature stop codon leading to elimination of ~11.6% of the peptide sequence.	No (reported in ClinVar)	1070373
**Small Deletion**	Exon 8	c.1302delA	p.Ala435Glnfs*8	Likely Pathogenic	Altered reading frame leads to premature truncation ~6.9% of peptide sequence	Yes [170]	2133466

**Missense**	Exon 8	c.1309G>A	p.Glu437Lys	Pathogenic	*In vitro* study in COS-7 cells found that this variant is normally synthesized but has completely abolished catalytic activity [204].<br><br>Variant has been observed in an FCS patient found to be compound heterozygous for this variant and for the pathogenic missense variant LPL:c.829G>A:p.Asp277Asn [204].<br><br>Another pathogenic missense variant, LPL:c.1310A>T:p.Glu437Val, has been observed affecting this codon, providing additional indirect evidence for a deleterious effect of altering this codon.	Yes [204]	2691331
**Missense**	Exon 8	c.1310A>T	p.Glu437Val	Pathogenic	*In vitro* study in HEK293 cells found that this variant produced enzyme that was normally synthesized but only displayed activity around roughly 11-23% of the wild-type level [205]. A further *in vitro* investigation in CHO-K1 cells found that this variant renders the synthesized peptide highly susceptible to endoproteolytic cleavage at residue 324, separating the N-terminal catalytic domain from the C-terminal GPIHBP1 binding domain [206].<br><br>Variant has been observed in a FCS patient found to be homozygous for this variant [205].<br><br>Another pathogenic missense variant, LPL:c.1309G>A:p.Glu437Lys, has been observed affecting this codon, providing additional indirect evidence for a deleterious effect of altering this codon.	Yes [205,206]	-
**Small Deletion**	Exon 8	c.1314_1315delTC	p.Gln439Glufs*8	Likely Pathogenic	Altered reading frame leads to premature truncation eliminating ~6.1% of peptide sequence	Yes [207]	-
**Nonsense**	Exon 8	c.1315C>T	p.Gln439Term	Likely Pathogenic	Substitution produces a premature stop codon leading to elimination of ~7.8% of the peptide sequence	No (reported in ClinVar)	1324676
**Splicing**	Intron 8	c.1322+1G>A	N/A	Likely Pathogenic	*In vitro* exon trapping assay in COS-7 cells found that this variant abolishes the donor splice site of intron 8, leading to aberrant splicing that results in loss of 134 nucleotides in exon 8 which in turn results in the loss of 45 amino acids from the LPL peptide sequence [30]. Further *in vitro* investigations in COS-7 cells showed that this variant has significantly lower mRNA expression than wild-type level, indicating that this variant impacts gene transcription [30]. Additionally, it was shown that LPL mass in cell media and lysate was similar for this variant and wild-type, but	Yes [30]	-

					catalytic activity was significantly decreased in cell media compared to wild-type [30].<br><br>Variant was observed in 2 unrelated compound heterozygous individuals presenting with severe HTG and pancreatitis [30]. Proband 1 was identified to be a compound heterozygote for this variant and the likely pathogenic nonsense variant LPL:c.162C>A:p.Cys54Term while proband 2 was found to be a compound heterozygote for this variant and the pathogenic missense variant LPL:c.835C>G:p.Leu279Val [30].		
**Splicing**	Intron 8	c.1322+1G>C	N/A	Pathogenic	Likely abolishes the donor splice site for intron 8, likely leading to defective pre-mRNA splicing.<br><br>Another pathogenic splicing variant at this nucleotide, LPL:c.1322+1G>A, has been identified and shown to abolish this donor splice site of intron 8 [30].	No (reported in ClinVar)	1423003
**Splicing**	Intron 8	c.1322+2T>C	N/A	Likely Pathogenic	Sequencing of cDNA produced by reverse transcribing mRNA from a heterozygous carrier of this variant showed that this variant abolishes the donor splice site of intron 8, which led to the utilization of a cryptic splice site in exon 8, which resulted in the loss of 134 nucleotides from exon 8 and intron 8, which ultimately resulted in the production of a frame shift resulting in a premature stop codon [88].<br><br>Variant has been reported in an FCS patient found to be compound heterozygous for this variant and for the likely pathogenic missense variant LPL:c.542G>T:p.Gly181Val [88]. It has also been observed in several heterozygous carriers of this proband's family [89].	Yes [88,89]	-
**Splicing**	Intron 8	c.1322+2T>G	N/A	Likely Pathogenic	Likely abolishes the donor splice site for intron 8, likely leading to defective pre-mRNA splicing.<br><br>Another likely pathogenic splicing variant at this nucleotide, LPL:c.1322+2T>C, has been identified and shown to abolish this donor splice site [88].	No (reported in ClinVar)	1769926
**Splicing**	Intron 8	c.1322+15T>G	N/A	Likely Pathogenic	Functional data are unavailable.<br><br>SpliceAI strongly predicts that this variant results in a donor gain, while likely leads to aberrant splicing.<br><br>Variant has been observed in two FCS patients (siblings) found to both be homozygous for this variant [136].	Yes [136]	-

**Gross Deletion**	N/A	(2.1 kb incl. ex. 9)	N/A	Pathogenic	Deletion of the 3' end of intron 8, all of exon 9, and a large portion of intron 9.	Yes [208]	-
**Missense**	Exon 9	c.1334G>A	p.Cys445Tyr	Pathogenic	*In vitro* study in COS cells found that this variant results in 48% reduction in LPL activity in cell media with a similar reduction for LPL mass in cell media when compared to wild-type, giving a normal specific activity for this variant [209]. Further *in vitro* studies in CHO-K1 cells determined that this variant abolishes ability of LPL to bind to GPIHBP1 without impacting catalytic activity and prevents the transportation of LPL to the apical surface of endothelial cells [210].<br><br>Variant was observed in FCS patient found to be compound heterozygous for this variant and the pathogenic missense variant LPL:c.662T>C:p.Ile221Thr [209].	Yes [52,209, 210]	1553
**Missense**	Exon 9	c.1342G>A	p.Glu448Lys	Likely Pathogenic	*In vitro* study in COS cells found that this variant led to mild reduction in LPL activity and mass in cell media to ~70% and ~77%, respectively, of wild-type level [211]. Later *in vitro* study in CHO-K1 cells determined that this variant abolishes ability of LPL to bind to GPIHPBP1 without impacting catalytic activity and prevents the transportation of LPL to the apical surface of endothelial cells [210].<br><br>Variant was observed in a heterozygous patient who presented with and passed away from pregnancy-induced hypertriglyceridemic pancreatitis in the final trimester of pregnancy [211].	Yes [210,211]	1684868
**Small Deletion**	Exon 9	c.1373delC	p.Ala458Aspfs*6	Likely Pathogenic	Altered reading frame leads to premature truncation eliminating ~2.5% of peptide sequence	Yes [21]	-
**Missense**	Exon 9	c.1394G>A	p.Cys465Tyr	Likely Pathogenic	*In vitro* study in CHO-K1 cells found that this variant reduced the ability of LPL to bind to GPIHBP1 by 70-80% compared to wild-type LPL [210].<br><br>Variant has been observed in a severe HTG patient found to be homozygous for this variant [212].	Yes [210,212]	1301315
**Splicing**	Intron 9	c.1427+1G>C	N/A	Likely Pathogenic	Likely abolishes the donor splice site of intron 9, likely leading to defective pre-mRNA splicing.	No (reported in our clinic)	-

[a] N/A = Not Applicable

[b] For frameshift variants resulting in a premature stop codon, the notation ‘fs∗(number)’ indicates that the frameshift variant results in stop codon at (number) of residues downstream of the variant site.

***Supplementary Table S2. Curated List of Variants of Uncertain Significance in* LPL.**

Variant Type	Exon/ Intron	Nucleotide Change	Amino Acid Change	ACMG Classification [a,b]	Molecular Defect and Evidence Notes	Previously reported in literature?	ClinVar ID
**Regulatory**	N/A	c.-241G>C	N/A	VUS	*In vitro* study in THP-1 and C2C12 cells showed that this variant reduced *LPL* promoter activity by 70-75% [213].<br><br>Variant was initially identified in a heterozygous FCHL patient with post-heparin LPL mass and activity ~50% less than controls [213].	Yes [52,213,214]	-
**Missense**	Exon 1	c.26T>G	p.Leu9Arg	VUS*	Functional data are unavailable.<br><br>Reported in heterozygous state in one proband and one relative, both with severe HTG [131].	Yes [131]	-
**Missense**	Exon 2	c.112G>A	p.Glu38Lys	VUS	Functional data are unavailable.<br><br>Variant was initially observed in 4 patients sequenced with the LipidSeq panel [12,13]. Two of four patients identified with LipidSeq were non-HTG cases.	Yes [12,13]	755163
**Missense**	Exon 2	c.113A>G	p.Glu38Gly	VUS	Functional data are unavailable.<br><br>Variant has been observed by us [12,181] in an MCS patient.	Yes [12,181]	-
**Missense**	Exon 2	c.134C>A	p.Thr45Asn	VUS	Functional data are unavailable.<br><br>Reported initially in a patient identified in a severe HDL deficiency cohort who also had mild-to-moderate HTG [214]. Patient had mild-to-moderate HTG.	Yes [214]	908634
**Missense**	Exon 2	c.143A>T	p.Asp48Val	VUS	*In vitro* study in COS-1 cells identified that this variant yielded a similar LPL protein mass and activity to wild-type, indicating that the variant seems to have negligible impact of LPL catalytic activity [215].<br><br>Variant was initially reported in a patient with FCHL [215]. We have also observed this variant in our clinic [12] in a heterozygote diagnosed with MODY.	Yes [12,215]	3379804
**Missense**	Exon 2	c.154G>C	p.Asp52His	VUS*	Functional data are unavailable.<br><br>We have previously observed this variant in our clinic [12,14] in a patient that was diagnosed with MCS.	Yes [12,14]	-
**Missense**	Exon 2	c.173C>G	p.Pro58Arg	VUS	Functional data are unavailable.<br><br>Variant was reported in a double heterozygote for this variant and the likely pathogenic *APOA5* variant	Yes [216]	-

					APOA5:c.161+5G>C in a patient with severe HTG and acute pancreatitis [216]. However, the *APOA5* variant alone could be enough to cause the patient phenotype, as it has been previously reported in homozygosity to cause FCS [217], and heterozygosity for deleterious *APOA5* variants has been previously shown by us to be associated with severe HTG, usually in the presence of secondary factors [218].		
**Missense**	Exon 2	c.190G>A	p.Val64Met	VUS*	Functional data are unavailable.<br><br>Variant was reported in a heterozygous patient with mild-to-moderate HTG who also carried the common *LPL* GOF variant, LPL:c.1421C>G:p.Ser474Term [18].	Yes [18]	1209598
**Missense**	Exon 2	c.211C>T	p.His71Tyr	VUS	*In vitro* study in COS-1 cells found that this variant yielded LPL protein mass and activity similar to wild-type [215].<br><br>Variant was first detected in a FCHL patient with mild-to-moderate HTG with normal plasma LPL mass and activity [215].	Yes [215]	-
**Missense**	Exon 2	c.214A>G	p.Ser72Gly	VUS*	Functional data are unavailable. Variant was first reported in compound heterozygosity with the likely pathogenic *LPL* variant LPL:c.913T>C:p.Cys305Arg in an FCS patient [184]. Post-heparin plasma LPL mass was found to be ~10% of wild-type level [184]. Father of this proband also presented with severe HTG [184].	Yes [184]	-
**Missense**	Exon 2	c.215G>A	p.Ser72Asn	VUS	Functional data are unavailable.<br><br>Variant was first reported in a heterozygote with mild-to-moderate HTG and was found to be inherited from their father who possessed a similar lipid profile as the child [219].	Yes [219]	-
**Missense**	Exon 3	c.362A>C	p.Tyr121Ser	VUS*	Functional data are unavailable.<br><br>Variant was first reported in a patient with mild-to-moderate HTG [5].	Yes [5]	-
**Missense**	Exon 3	c.370T>C	p.Ser124Pro	VUS*	Functional data are unavailable.<br><br>Variant was first identified by us [12] but beyond the presence of HTG in this patient, further details are not available this time.	Yes [12]	1333456
**Missense**	Exon 3	c.373G>A	p.Ala125Thr	VUS*	Functional data are unavailable.<br><br>Original report of this variant (Jap, T. S., S. F. Jenq, Y. C. Wu, and J. Y. Chiou. 1999. Human gene mutations. Gen symbol:	Yes [2,220]	1684861

					LPL. Disease: Lipoprotein lipase deficiency. Hum. Genet. 104: 289) seems to no longer be available online. However, some details regarding this report may be gleaned from the supplemental material of Merkel, Eckel, and Goldberg's 2002 review which cites the original report [220]. Based on the list of *LPL* variants and their references provided in the supplemental material of this review, this variant was reported in at least one compound heterozygote with chylomicronemia however the other variant is not known for certain [220].		
**Missense**	Exon 3	c.423G>C	p.Trp141Cys	VUS*	Functional data are unavailable.<br><br>*In silico* analysis reported in ClinVar entry supports a deleterious effect for this variant on protein structure/function.	No (reported in ClinVar)	1212446
**Missense**	Exon 4	c.451G>A	p.Asp151Asn	VUS*	Functional data are unavailable.<br><br>Variant was reported in a proband of their family all presenting with FCHL and was found to co-segregate with FCHL in this family, with at least 6 relatives with mild-to-moderate HTG carrying the variant [221].	Yes [221]	-
**Missense**	Exon 4	c.534A>C	p.Arg178Ser	VUS*	Functional data are unavailable.<br><br>Variant was initially observed in a patient sequenced on LipidSeq [12] presenting with HTG.	Yes [12]	-
**Missense**	Exon 5	c.586A>T	p.Ser196Cys	VUS	Functional data are unavailable.<br><br>Variant was reported in a heterozygote with mild-to-moderate HTG [18].	Yes [18]	-
**Missense**	Exon 5	c.637A>G	p.Thr213Ala	VUS*	Functional data are unavailable.<br><br>Variant is reported in a review article [2] without clinical data and has an entry in ClinVar but no other reports seem to be available.	Yes [2]	2136642
**Missense**	Exon 5	c.641G>T	p.Arg214Ile	VUS*	Functional data are unavailable.<br><br>Variant is only reported as a submission in ClinVar and LOVD3, but no accompanying clinical data is available.	No (reported in ClinVar and LOVD3)	2433509
**Missense**	Exon 5	c.656G>A	p.Arg219Gln	VUS	Functional data are unavailable.<br><br>Variant was first reported in a population level study of *LPL* SNPs and their association with CAD in the Chinese population [222]. They found that this variant is significantly associated with a low HDL and high TG phenotype [222].	Yes [2,222]	-

**Missense**	Exon 5	c.661A>T	p.Ile221Phe	VUS*	Functional data are unavailable.<br><br>One pathogenic missense variant has been observed impacting this codon, LPL:c.662T>C:p.Ile221Thr, which gives indirect evidence for a deleterious impact of altering this codon.	No (reported in ClinVar)	1066136
**Missense**	Exon 5	c.662T>A	p.Ile221Asn	VUS*	Functional data are unavailable.<br><br>One pathogenic missense variant has been observed impacting this codon, LPL:c.662T>C:p.Ile221Thr, which gives indirect evidence for a deleterious impact of altering this codon.	No (reported in ClinVar)	2583060
**Missense**	Exon 5	c.667A>T	p.Ile223Phe	VUS*	Functional data are unavailable.<br><br>Variant has only been reported in a heterozygote with mild-to-moderate HTG [5].	Yes [5]	-
**Missense**	Exon 5	c.674A>G	p.Lys225Arg	VUS	Functional data are unavailable.<br><br>The original report of this variant is unknown; we only know that it was identified in a Japanese patient with LPL deficiency as was reported in one review [134]. No other details, including clinical details, zygosity, etc. are known.	Yes [134]	-
**Missense**	Exon 5	c.691G>A	p.Asp231Asn	VUS*	Functional data are unavailable.<br><br>Two likely pathogenic missense variants have been observed impacting this codon, LPL:c.691G>C:p.Asp231His and LPL:c.693C>G:p.Asp231Glu, providing indirect evidence for a deleterious impact of altering this codon.	No (reported in ClinVar)	1685930
**Missense**	Exon 5	c.717T>A	p.Phe239Leu	VUS	Functional data are unavailable.<br><br>Variant was first reported in a patient sequenced on LipidSeq by us [12] who had HTG.	Yes [12]	-
**Missense**	Exon 6	c.811T>C	p.Ser271Pro	VUS	Functional data are unavailable.<br><br>Variant was reported in compound heterozygosity with the pathogenic gross insertion variant LPL:c.898_1018+1939dup but the clinical data and diagnosis of this patient are unclear [178].	Yes [178]	-
**Missense**	Exon 6	c.818A>C	p.His273Pro	VUS	Functional data are unavailable.<br><br>One pathogenic missense variant has been observed impacting this codon, LPL:c.818A>G:p.His273Arg, which gives indirect evidence for a deleterious impact of altering this codon.	No (reported in ClinVar)	2429774

**Missense**	Exon 6	c.826A>G	p.Ile276Val	VUS	Functional data are unavailable.<br><br>Variant is reported in a review article with no accompanying clinical data as a "variant that does not necessarily underlie LPL deficiency" [2].	Yes [2]	-
**Missense**	Exon 6	c.827T>C	p.Ile276Thr	VUS*	Functional data are unavailable, thus exact molecular defection is unknown, if any. Unfortunately, the original report the found this variant seems to no longer be available online (Jap, T. S., S. F. Jenq, Y. C. Wu, and J. Y. Chiou. 1999. Human gene mutations. Gen symbol: LPL. Disease: Lipoprotein lipase deficiency. Hum. Genet. 104: 289). However, some details regarding this report may be gleaned from the supplemental material of Merkel, Eckel, and Goldberg's 2002 review [220]. Based on the list of *LPL* variants and their references provided in the supplemental material of this review, this variant was reported in at least one compound heterozygote with chylomicronemia however the other variant is not known for certain [220].	Yes [2,220]	3340534
**Missense**	Exon 6	c.877T>C	p.Ser293Pro	VUS	Functional data are unavailable.<br><br>Digital version of the original report of this variant (Wiebusch H, Funke H, Kastelein JJP, et al.: Mutations in the lipoprotein lipase gene are not restricted to patients with type I hyperlipidemia. Circulation 1992, 86:609.) is unavailable but a review by Murthy et al. includes a description of the effect of this variant as inactivating LPL [143].	Yes [143]	1684866
**Missense**	Exon 6	c.886G>A	p.Ala296Thr	VUS	Functional data are unavailable.<br><br>Variant was identified in a group of patients with well-established molecular diagnoses (presumably related to dyslipidemia) used to validate a NGS workflow but clinical data regarding the heterozygous carrier of this variant are not provided [109].	Yes [109]	1684867
**Missense**	Exon 6	c.898G>C	p.Gly300Arg	VUS*	Functional data are unavailable.<br><br>This variant was initially reported by us [12] in a severe HTG patient found to be heterozygous for this variant.	Yes [12]	2065173
**Missense**	Exon 6	c.911G>A	p.Ser304Asn	VUS	Functional data are unavailable.<br><br>No reports of this variant provide clinical data for the carrier(s) of this variant [5,223,224].	Yes [5,223,224]	-
**Missense**	Exon 6	c.952A>G	p.Asn318Asp	VUS	Functional data are unavailable.	Yes [12]	-

					Variant was initially reported by us [12] in a severe HTG patient found to be heterozygous for this variant.		
**Missense**	Exon 6	c.984G>A	p.Met328Ile	VUS	Functional data are unavailable.<br><br>Three likely pathogenic missense variants have been observed impacting this codon, LPL:c.983T>C:p.Met328Thr, LPL:c.983T>G:p.Met328Arg, and LPL:c.984G>T:p.Met328Ile, providing indirect evidence for a deleterious impact of altering this codon.	No (reported in LOVD3)	-
**Missense**	Exon 6	c.995C>T	p.Thr332Ile	VUS	Functional data are unavailable.<br><br>Variant has not been reported in literature.	No (reported in ClinVar)	1206794
**Missense**	Exon 6	c.997C>T	p.Arg333Cys	VUS*	Functional data are unavailable.<br><br>One pathogenic missense variant has been observed impacting this codon, LPL:c.998G>A:p.Arg333His, providing indirect evidence for a deleterious impact of altering this codon.	No (reported in LOVD3)	-
**Missense**	Exon 6	c.1015A>C	p.Lys339Gln	VUS	Functional data are unavailable.<br><br>Initial report of this variant identified it in a heterozygote with HTG (severity is unclear but is ≥5.65 mmol/L based on subject selection criteria) [8].	Yes [8]	-
**Missense**	Exon 6	c.1018G>T	p.Val340Phe	VUS	Functional are unavailable.<br><br>Variant impacts the final nucleotide of exon 6, which forms a portion of the canonical donor splice site for intron 6. SpliceAI strongly predicts an alternative splicing consequence for this variant.<br><br>Variant has been reported by us previously [12], in a heterozygote with HTG.	Yes [12]	-
**Missense**	Exon 7	c.1023C>A	p.Phe341Leu	VUS	Functional data are unavailable.<br><br>Variant has only been observed by us [12] in a patient with mildly elevated TG.	Yes [12]	-
**Missense**	Exon 7	c.1033G>A	p.Val345Ile	VUS	Functional data are unavailable.<br><br>Variant was initially reported by us in two patients sequenced on LipidSeq [12,181], of which only one presented with HTG.	Yes [12,181]	1771919
**Missense**	Exon 7	c.1049C>G	p.Ser350Cys	VUS	Functional data are unavailable.	Yes [225]	-

					Unfortunately, only the abstract of the original report of this variant is available to us, which identified that at least one patient with mild HTG was a carrier of this variant [225].		
**Missense**	Exon 7	c.1094C>T	p.Ser365Phe	VUS*	*In vivo* post-heparin analysis indicated that this variant did not impact LPL activity nor mass compared to wild-type and follow-up *in vitro* experiment found that this variant increased specific activity of LPL while mildly impairing secretion, with the final conclusion drawn that the increased specific activity compensated for the reduced secretion observed [56].<br><br>Variant was initially reported in a heterozygote identified in a hypertriglyceridemia cohort [56]. We also observed this variant in patient with HTG sequenced on LipidSeq [12].	Yes [12,56]	2627200
**Missense**	Exon 7	c.1108G>A	p.Val370Met	VUS	No functional data are available.<br><br>Variant is reported in a review article [2] without clinical data.	Yes [2]	2084792
**Missense**	Exon 7	c.1122G>C	p.Glu374Asp	VUS	Functional data are unavailable.<br><br>Variant was identified in a patient with Type III hyperlipidemia with mild-to-moderate HTG [5].	Yes [5]	-
**Missense**	Exon 7	c.1123A>G	p.Asn375Asp	VUS	Functional data are unavailable.<br><br>Variant was initially reported by us in patients sequenced on LipidSeq [12,13], but neither of the two patients we identified were HTG cases.	Yes [12,13]	1343454
**Missense**	Exon 8	c.1183A>G	p.Thr395Ala	VUS	Functional data are unavailable.<br><br>We previously identified this variant in a CAD patient sequenced on LipidSeq [12,13] but the patient had normal TG levels.	Yes [12,13]	-
**Small Deletion**	Exon 8	c.1267_1272 delAGTCCC	p.Ser423_Pro42 4del	VUS	Functional data are unavailable.<br><br>Variant was identified in a patient with familial LPL deficiency, however the patient was compound heterozygous for 2 other pathogenic *LPL* variants upstream of this variant on both alleles (LPL:c.701C>T:p.Pro234Leu and LPL:c.835_836delCT:p.Leu279Valfs*3) so the impact of this variant is unclear [66].	Yes [66]	-
**Missense**	Exon 8	c.1295G>C	p.Arg432Thr	VUS	Functional data are unavailable.<br><br>Variant was identified in a heterozygote with severe HTG [159].	Yes [159]	3234449

**Missense**	Exon 8	c.1302A>T	p.Lys434Asn	VUS	Functional data are unavailable.<br><br>Variant was first reported in a patient with severe HTG found to possess this variant and LPL:c.1306G>C:p.Gly436Arg on the same allele [226]. Post-heparin plasma analysis were unable to detect LPL activity in this patient but rather interestingly, the patient displayed LPL mass at a level 140% that of the mean LPL mass obtained from 12 healthy controls [226]. We have also observed a patient with this same genotype in our clinic [12].<br><br>This variant and LPL:c.1306G>C:p.Gly436Arg are frequently observed together.	Yes [12,226]	158104
**Small Indel**	Exon 8	c.1302delinsTTTTT	p.Lys434delinsAsnPhe	VUS*	Functional data are unavailable.<br><br>We have previously observed this variant in a patient sequenced on LipidSeq [12].	Yes [12]	-
**Missense**	Exon 8	c.1306G>A	p.Gly436Arg	VUS*	Functional data are unavailable.<br><br>Variant was first reported in a patient with severe HTG found to possess this variant and LPL:c.1302A>T:p.Lys434Asn on the same allele [226]. Post-heparin plasma analysis were unable to detect LPL activity in this patient but rather interestingly, the patient displayed LPL mass at a level 140% that of the mean LPL mass obtained from 12 healthy controls [226]. We have also observed a patient with this same genotype in our clinic [12].<br><br>This variant and LPL:c.1302A>T:p.Lys434Asn are frequently observed together.	Yes [12,226]	158100
**Missense**	Exon 8	c.1306G>C	p.Gly436Arg	VUS	Functional data are unavailable.<br><br>Variant has not been reported in the literature. We have observed this variant in a patient being treated at our clinic with HTG.	No (reported in ClinVar)	1685369
**Missense**	Exon 9	c.1325T>G	p.Val442Gly	VUS	Functional data are unavailable.<br><br>The only other study that reports this variant lists it as a potentially deleterious variant in relation to acute myocardial infarction [227].	Yes [227]	766071
**Missense**	Exon 9	c.1397A>C	p.His466Pro	VUS	Functional data are unavailable.<br><br>Variant has not been reported in the literature.	No (reported in ClinVar)	1196534

[a] VUS = Variants of Uncertain Significance

[b] VUS* = VUS variants for which additional case and/or co-segregation data may potentially allow for variant classification to be upgraded to likely pathogenic and/or pathogenic in the future.

Supplementary Table S3. Select Benign/Likely Benign Variants Reported in Association with Various TG Phenotypes

Variant Type	Exon/ Intron	Nucleotide Change	Amino Acid Change	ACMG Classification	Molecular Defect Notes (and others where needed)	Previously reported in literature?	ClinVar ID
**Regulatory**	N/A[a]	c.-281T>G	N/A	Likely Benign	*In vitro* study in THP-1 cells found that this variant reduced *LPL* promoter activity by 40-50% compared to wild-type [4,213].<br><br>Variant was initially reported in an FCHL patient who was compound heterozygous for this variant and the likely pathogenic promoter region variant LPL:c.-227T>C [4]. Interestingly, a large-scale meta-analysis and review later found no associations of this variant with lipid levels nor with CHD risk [114]. More recent work has actually associated this variant with a protective effect, specifically finding an association between this variant and lower third trimester plasma TG levels in pregnant women of African American descent [228].<br><br>Additionally, it should be noted that this variant has been observed in linkage disequilibrium with LPL:c.106G>A:p.Asp36Asn [213,229,230].<br><br>Variant has been reported in population databases at higher frequency than is expected for disease for this gene, including 646 homozygous individuals reported in gnomAD [231].	Yes [4,52,114,213,228]	-
**Small Insertion**	N/A	c.-172_-171dupCC	N/A	Benign	*In vitro* study in THP-1 cells showed that this variant reduces *LPL* promoter activity by 20% while a 50% reduction was observed in C2C12 cells [213].<br><br>Variant was initially reported in a heterozygous FCHL patient with reduced post-heparin plasma LPL levels [213].<br><br>However, this variant has been reported in population databases at higher frequency than expected for disease for this gene, including the reporting of 2 homozygous individuals in gnomAD [231].	Yes [52,213]	362400
**Missense**	Exon 2	c.106G>A	p.Asp36Asn	Benign	*In vitro* study in COS cells found that this variant reduces LPL activity and mass by 20 to 30% but only slightly reduced specific activity by 18% [232]. However, another *in vitro* study in COS-1 cells observed no significant differences in specific activity when compared to wild-type [233].<br><br>Several studies have found modest associations between this variant and HTG [52,97,114,230,232–235]. One study found that the association of this variant with HTG was rendered not	Yes [3,52,97,114–116,230,232–246]	1552

					significant when diabetic individuals were excluded [233] while another study found that the LPL:-281T>G/LPL:c.106G>A:p.Asp36Asn haplotype was only associated with HTG specifically in male carriers in a FCHL cohort [235].<br><br>This variant has been observed in linkage disequilibrium with LPL:c.-281T>G [213,229,230].<br><br>This variant has been reported in population databases at higher frequency than expected for disease for this gene, including the reporting of 60 homozygous individuals in gnomAD [231].		
**Missense**	Exon 2	c.182C>T	p.Ala61Val	Likely Benign	Functional data are unavailable.<br><br>Variant was first reported in homozygous state in two brothers with severe HTG, low HDL, and no detectable post-heparin LPL activity [18].<br><br>However, *in silico* analysis done by these authors supported a benign or tolerated effect for this variant [18]. Thus, it was hypothesized to be in linkage with an *LPL* variant that was not identified in their study [18]. This variant was reported again several years later in an FCS patient that was a double homozygote for this variant and for the pathogenic splicing variant LPL:c.250-1G>C [24]. It is likely that this variant is in linkage disequilibrium with the LPL:c.250-1G>C splicing variant.	Yes [18,24]	-
**Missense**	Exon 2	c.213C>G	p.His71Gln	Benign	Functional data are unavailable.<br><br>Variant has been reported to be associated with increased risk for HTG [247]. However, according to calculations reported in the ClinVar entry for this variant, the 95% confidence interval for the odds ratio of this finding in one study overlaps 1, thus the confidence in this finding is reduced.<br><br>This variant is also reported at greater frequency than would be expected for disease in normal population databases [231].	Yes [2,13,247]	495743
**Splicing**	Intron 3	c.430-6C>T	N/A	Benign	Functional data are unavailable.<br><br>Six HTG patients, including 1 severe HTG patient, were found to be heterozygous for this variant [10] but 4 of these patients also presented with secondary TG elevating factors such as non-insulin dependant diabetes mellitus, suggesting that if this variant induces HTG it is in combination with other factors [10].	Yes [10]	362408

					This variant has been reported in population databases at higher frequency than expected for disease for this gene, including the reporting of 43 homozygous individuals in gnomAD [231].		
**Missense**	Exon 6	c.953A>G	p.Asn318Ser	Benign	Multiple *In vitro* studies have found that this variant results in reduced LPL catalytic activity [248–250].<br><br>Multiple studies have reported modest associations between this variant and HTG [97,114,250,251]. In one study, it was found that hyperinsulinemia or diabetes mellitus were required to precipitate HTG in heterozygotes for this variant [250].<br><br>This variant has been reported in population databases at higher frequency than expected for disease for this gene, including the reporting of 36 homozygous individuals in gnomAD [231].	Yes [12,17,19,52,81,97,114,115,234,242,244,245,248–260]	1550
**Intronic**	Intron 6	c.1018+34A>G	N/A	Benign	Functional data are unavailable.<br><br>Variant was first reported in a type 1 diabetes mellitus patient with severe HTG and ketoacidosis [261].<br><br>This variant has been reported in population databases at higher frequency than expected for disease for this gene, including the reporting of 8 homozygous individuals in gnomAD [231].	Yes [117,261]	1165810
**Missense**	Exon 7	c.1134C>G	p.Phe378Leu	Likely Benign	*In vitro* study in HEK293 cells found no difference in LPL mass nor activity in cell media of cells expression this variant versus wild-type [127].<br><br>Variant was found in an severe HTG patient found to be heterozygous for this variant [127].	Yes [127]	-
**Missense**	Exon 7	c.1135A>G	p.Thr379Ala	Benign	Functional data are unavailable.<br><br>Variant has been reported in a FCHL study but specific clinical data regarding the carrier of this variant were not provided [247].<br><br>This variant has been reported in population databases at higher frequency than expected for disease for this gene, including the reporting of 2 homozygous individuals in gnomAD [231].	Yes [2,247]	495741
**Missense**	Exon 7	c.1136C>T	p.Thr379Ile	Benign	Functional data are unavailable.<br><br>Digital version of the original report of this variant (Wiebusch H, Funke H, Kastelein JJP, et al.: Mutations in the lipoprotein lipase gene are not restricted to patients with type I hyperlipidemia. Circulation 1992, 86:609.) is unavailable. Variant has been identified in a severe HTG patient found to be heterozygous for this variant but clinical details are not provided [52].	Yes [52,143]	495742

					This variant has been reported in homozygous state in population databases at higher frequency than expected for disease with 3 homozygous individuals found in gnomAD [231].		
**Missense**	Exon 8	c.1279G>A	p.Ala427Thr	Benign	Functional data are unavailable.<br><br>Variant has been reported in two patients diagnosed with FCS but *in silico* analysis performed by these authors found no evidence for a deleterious effect of this variant [81]. Post-heparin plasma LPL activity was normal in carriers of this variant [81]. This variant has also been reported in heterozygous state in a severe HTG patient [18].<br><br>This variant has been reported in population databases at higher frequency than expected for disease for this gene, including the reporting of 5 homozygous individuals in gnomAD [231].	Yes [18,81]	362414
**Intronic**	Intron 8	c.1322+483T>G	N/A	Benign	*In vitro* study in human smooth muscle cells and COS-1 cells found that this variant is associated with a ~20% reduction in gene transcription [262]. It was also shown that this reduction is likely due to this variant altering a transcription factor binding site in intron 8, reducing it's affinity for the transcription factor [262].<br><br>This variant has been reported in population databases at higher frequency than expected for disease for this gene, including the reporting of 1214 homozygous individuals in gnomAD [231].	Yes [114,262–264]	1165812
**Intronic**	Intron 8	c.1323-90T>G	N/A	Benign	*In vitro* study in Huh7 cells found that this variant is associated with a ~1.7 times higher expression than the wild-type allele [253]. These authors also identified that there is a high likelihood this variant impacts an important regulatory region which would explain the observed effect [253].<br><br>This variant has been reported in population databases at higher frequency than expected for disease for this gene, including the reporting of 1531 homozygous individuals in gnomAD [231].	Yes [253,265]	1165815
**Nonsense**	Exon 9	c.1421C>G	p.Ser474Term	Benign	There is a large body of evidence indicating that this variant is a gain-of-function (GOF) variant. As summarized by Rip and colleagues [99], there is evidence for multiple modes by which this variant has been found to exert of beneficial effect on plasma TG levels, with increased activity and mass in plasma observed in multiple studies they reviewed. Additionally, it has been shown that this variant may reduce inhibition of translation [266]. This variant has also been reported to be in strong linkage disequilibrium with a number of 3' UTR SNPs in *LPL* [267] and it has been suggested that perhaps the TG-lowering effect of this	Yes [52,97,109,114,138,233,236,238,243,256,264,266,269–304]	1534

					variant may be explained at least in part by this linkage as it has been shown that a haplotype containing these SNPs abolished the ability of multiple miRNAs to inhibit LPL post-transcriptionally [268].<br><br>This variant has been reported in population databases at higher frequency than expected for disease for this gene, including the reporting of 1323 homozygous individuals in gnomAD [231].		
**Regulatory**	N/A	c.*371T>C	N/A	Benign	*In vitro* study in Huh7 cells found that this variant is associated with ~1.6-fold higher than the wild-type allele [253]. These authors also identified that there is a high likelihood this variant impacts an important regulatory region which would explain the observed effect [253].<br><br>This variant has been reported in population databases at higher frequency than expected for disease for this gene, including the reporting of 40 homozygous individuals in gnomAD [231].	Yes [253,265,304]	362422
**Small Deletion**	N/A	c.*414_*418del CTCTA	N/A	Likely Benign	*In vitro* study in COS-1 cells and human aorta smooth muscle cell line found that this variant modestly reduced *LPL* gene expression and a slight reduction in translation [305].<br><br>Variant was found to delete 5 nucleotides from a putative insulin response element in the 3' UTR of LPL encoded in exon 10 [305].	Yes [305]	362423
**Regulatory**	N/A	c.*1224C>A	N/A	Benign	Variant was determined to abolish miRNA binding site in LPL, which may increase LPL expression [306].<br><br>This variant has been reported in population databases at higher frequency than expected for disease for this gene, including the reporting of 113 homozygous individuals in gnomAD [231].	Yes [306]	362440
**Regulatory**	N/A	c.*1671T>C	N/A	Benign	Variant was found to abolish miRNA-410-mediated LPL inhibition and is associated with lower plasma TG [307]. Another study has reported this variant is associated with reduced plasma TG and reduced stroke risk [308].<br><br>This variant has been reported in population databases at higher frequency than expected for disease for this gene, including the reporting of 2009 homozygous individuals in gnomAD [231].	Yes [304,307–309]	40131
**Regulatory**	N/A	c.*1742T>C	N/A	Benign	Variant leads to lower association of miR-136 with a miRNA binding motif in the 3' UTR of LPL [306]. Variant has been associated with lowered TG and higher LPL mRNA and protein expression levels [310].	Yes [304,306,310]	362448

					This variant has been reported in population databases at higher frequency than expected for disease for this gene, including the reporting of 258 homozygous individuals in gnomAD [231].		
**Small Insertion**	N/A	c.*1806_*1807insTT	N/A	Benign	Variant was shown to lead to loss of a 3' UTR miRNA binding site that is associated with a larger LPL haplotype (Hap4) that is associated with lower TG than WT due to loss of miRNA-mediated LPL inhibition [268].<br><br>This variant has been reported in population databases at higher frequency than expected for disease for this gene, including the reporting of 252 homozygous individuals in gnomAD [231]	Yes [268]	362450

[a] N/A = Not Applicable

Supplementary Table S4. *LPL* Variants Reported in HGMD Excluded from Analysis and Review to Unavailable Case Data.

Variant Type	Exon/Intron	Nucleotide Change	Amino Acid Change
**Missense**	exon 1	c.8G>A	p.Ser3Asn
**Missense**	exon 1	c.35C>T	p.Ala12Val
**Missense**	exon 1	c.37G>T	p.Val13Leu
**Missense**	exon 1	c.38T>C	p.Val13Ala
**Missense**	exon 1	c.43C>A	p.Leu15Ile
**Missense**	exon 1	c.49A>G	p.Ser17Gly
**Missense**	exon 1	c.50G>A	p.Ser17Asn
**Missense**	exon 1	c.55A>C	p.Thr19Pro
**Missense**	exon 1	c.56C>T	p.Thr19Ile
**Missense**	exon 1	c.58G>A	p.Ala20Thr
**Missense**	exon 1	c.59C>T	p.Ala20Val
**Missense**	exon 1	c.68G>A	p.Gly23Glu
**Missense**	exon 1	c.71G>C	p.Gly24Ala
**Missense**	exon 1	c.83C>T	p.Ala28Val
**Missense**	exon 1	c.85G>C	p.Asp29His
**Missense**	exon 2	c.89A>G	p.Gln30Arg
**Missense**	exon 2	c.111C>G	p.Ile37Met
**Missense**	exon 2	c.109A>G	p.Ile37Val
**Missense**	exon 2	c.149C>G	p.Ala50Gly
**Missense**	exon 2	c.157A>G	p.Thr53Ala
**Missense**	exon 2	c.172C>T	p.Pro58Ser
**Missense**	exon 2	c.175G>A	p.Gly59Arg
**Missense**	exon 2	c.182C>A	p.Ala61Glu
**Missense**	exon 2	c.203A>G	p.His68Arg
**Missense**	exon 2	c.202C>T	p.His68Tyr
**Missense**	exon 2	c.219C>G	p.Ser73Arg
**Missense**	exon 2	c.226T>C	p.Phe76Leu
**Missense**	exon 2	c.248C>T	p.Thr83Met

**Missense**	exon 2	c.249G>A	p.Thr83Thr
**Missense**	exon 3	c.254C>G	p.Thr85Arg
**Missense**	exon 3	c.261G>A	p.Met87Ile
**Missense**	exon 3	c.267G>C	p.Glu89Asp
**Missense**	exon 3	c.268A>G	p.Ser90Gly
**Nonsense**	exon 3	c.273G>A	p.Trp91Term
**Missense**	exon 3	c.290C>T	p.Ala97Val
**Missense**	exon 3	c.303G>T	p.Lys101Asn
**Missense**	exon 3	c.305G>A	p.Arg102Lys
**Missense**	exon 3	c.311C>G	p.Pro104Arg
**Missense**	exon 3	c.315C>A	p.Asp105Glu
**Missense**	exon 3	c.325A>G	p.Ile109Val
**Missense**	exon 3	c.350C>T	p.Ala117Val
**Missense**	exon 3	c.355G>A	p.Glu119Lys
**Missense**	exon 3	c.374C>T	p.Ala125Val
**Missense**	exon 3	c.386A>C	p.Lys129Thr
**Missense**	exon 3	c.388C>A	p.Leu130Met
**Missense**	exon 3	c.395G>T	p.Gly132Val
**Missense**	exon 3	c.405G>A	p.Val135Val
**Missense**	exon 3	c.410G>A	p.Arg137Gln
**Missense**	exon 3	c.410G>T	p.Arg137Leu
**Missense**	exon 3	c.409C>T	p.Arg137Trp
**Missense**	exon 4	c.435G>A	p.Glu145Glu
**Missense**	exon 4	c.436T>A	p.Phe146Ile
**Missense**	exon 4	c.439A>C	p.Asn147His
**Missense**	exon 4	c.457G>C	p.Val153Leu
**Missense**	exon 4	c.473A>G	p.Tyr158Cys
**Missense**	exon 4	c.493G>T	p.Ala165Ser
**Missense**	exon 4	c.509G>A	p.Ser170Asn
**Missense**	exon 4	c.508A>T	p.Ser170Cys
**Missense**	exon 4	c.529A>G	p.Asn177Asp

**Missense**	exon 4	c.540T>C	p.Thr180Thr
**Missense**	exon 4	c.541G>T	p.Gly181Cys
**Missense**	exon 5	c.559C>T	p.Pro187Ser
**Missense**	exon 5	c.571T>G	p.Tyr191Asp
**Missense**	exon 5	c.572A>T	p.Tyr191Phe
**Missense**	exon 5	c.581C>G	p.Ala194Gly
**Missense**	exon 5	c.584C>T	p.Pro195Leu
**Missense**	exon 5	c.598C>T	p.Pro200Ser
**Missense**	exon 5	c.603T>G	p.Asp201Glu
**Missense**	exon 5	c.610G>A	p.Asp204Asn
**Missense**	exon 5	c.631A>T	p.Thr211Ser
**Missense**	exon 5	c.653G>A	p.Gly218Asp
**Missense**	exon 5	c.676C>A	p.Pro226Thr
**Missense**	exon 5	c.683G>C	p.Gly228Ala
**Missense**	exon 5	c.685C>G	p.His229Asp
**Missense**	exon 5	c.689T>C	p.Val230Ala
**Missense**	exon 5	c.713C>A	p.Thr238Asn
**Missense**	exon 5	c.734T>G	p.Ile245Ser
**Missense**	exon 5	c.742G>A	p.Ala248Thr
**Missense**	exon 5	c.748C>T	p.Arg250Cys
**Missense**	exon 5	c.749G>A	p.Arg250His
**Missense**	exon 5	c.751G>A	p.Val251Met
**Missense**	exon 5	c.769C>T	p.Leu257Phe
**Missense**	exon 6	c.799T>C	p.Ser267Pro
**Missense**	exon 6	c.815T>C	p.Ile272Thr
**Missense**	exon 6	c.846A>C	p.Glu282Asp
**Nonsense**	exon 6	c.844G>T	p.Glu282Term
**Missense**	exon 6	c.848A>G	p.Glu283Gly
**Missense**	exon 6	c.851A>G	p.Asn284Ser
**Missense**	exon 6	c.854C>T	p.Pro285Leu
**Missense**	exon 6	c.885A>C	p.Glu295Asp

**Missense**	exon 6	c.917G>C	p.Arg306Thr
**Missense**	exon 6	c.924C>A	p.Asn308Lys
**Missense**	exon 6	c.926G>A	p.Arg309His
**Missense**	exon 6	c.946G>A	p.Glu316Lys
**Missense**	exon 6	c.958G>T	p.Val320Phe
**Missense**	exon 6	c.976A>G	p.Ser326Gly
**Missense**	exon 6	c.1008G>A	p.Met336Ile
**Missense**	exon 7	c.1019T>C	p.Val340Ala
**Missense**	exon 6	c.1018G>A	p.Val340Ile
**Missense**	exon 7	c.1032A>T	p.Gln344His
**Missense**	exon 7	c.1039A>G	p.Ile347Val
**Missense**	exon 7	c.1045T>G	p.Phe349Val
**Missense**	exon 7	c.1048T>G	p.Ser350Ala
**Missense**	exon 7	c.1049C>T	p.Ser350Phe
**Missense**	exon 7	c.1059G>C	p.Glu353Asp
**Missense**	exon 7	c.1063G>A	p.Glu355Lys
**Missense**	exon 7	c.1067C>T	p.Thr356Ile
**Missense**	exon 7	c.1069C>G	p.His357Asp
**Missense**	exon 7	c.1109T>C	p.Val370Ala
**Missense**	exon 7	c.1114G>A	p.Glu372Lys
**Missense**	exon 7	c.1126A>T	p.Ile376Phe
**Missense**	exon 7	c.1137T>A	p.Thr379Thr
**Missense**	exon 7	c.1139T>C	p.Leu380Pro
**Missense**	exon 8	c.1147G>A	p.Val383Ile
**Missense**	exon 8	c.1154C>T	p.Thr385Ile
**Missense**	exon 8	c.1156A>G	p.Asn386Asp
**Missense**	exon 8	c.1163C>G	p.Thr388Ser
**Missense**	exon 8	c.1164C>A	p.Thr388Thr
**Missense**	exon 8	c.1165T>C	p.Tyr389His
**Missense**	exon 8	c.1166A>C	p.Tyr389Ser
**Missense**	exon 8	c.1169C>G	p.Ser390Cys

**Missense**	exon 8	c.1179T>G	p.Ile393Met
**Missense**	exon 8	c.1178T>C	p.Ile393Thr
**Missense**	exon 8	c.1180T>C	p.Tyr394His
**Missense**	exon 8	c.1186G>C	p.Glu396Gln
**Missense**	exon 8	c.1189G>A	p.Val397Ile
**Missense**	exon 8	c.1196T>C	p.Ile399Thr
**Missense**	exon 8	c.1201G>A	p.Glu401Lys
**Missense**	exon 8	c.1210A>G	p.Met404Val
**Missense**	exon 8	c.1214T>G	p.Leu405Trp
**Missense**	exon 8	c.1223A>T	p.Lys408Ile
**Missense**	exon 8	c.1230G>C	p.Lys410Asn
**Missense**	exon 8	c.1232G>C	p.Ser411Thr
**Missense**	exon 8	c.1234G>A	p.Asp412Asn
**Missense**	exon 8	c.1245T>G	p.Phe415Leu
**Missense**	exon 8	c.1260G>C	p.Trp420Cys
**Missense**	exon 8	c.1273G>A	p.Gly425Ser
**Missense**	exon 8	c.1286A>G	p.Gln429Arg
**Missense**	exon 8	c.1290G>T	p.Lys430Asn
**Missense**	exon 8	c.1291A>T	p.Ile431Phe
**Missense**	exon 8	c.1307G>C	p.Gly436Ala
**Missense**	exon 8	c.1319A>G	p.Lys440Arg
**Nonsense**	exon 8	c.1318A>T	p.Lys440Term
**Missense**	exon 9	c.1348G>C	p.Val450Leu
**Missense**	exon 9	c.1355A>G	p.His452Arg
**Missense**	exon 9	c.1356T>A	p.His452Gln
**Missense**	exon 9	c.1371G>C	p.Lys457Asn
**Missense**	exon 9	c.1373C>T	p.Ala458Val
**Missense**	exon 9	c.1375C>T	p.Pro459Ser
**Missense**	exon 9	c.1379C>T	p.Ala460Val
**Missense**	exon 9	c.1382T>C	p.Val461Ala
**Missense**	exon 9	c.1385T>C	p.Phe462Ser

**Missense**	exon 9	c.1401C>A	p.Asp467Glu
**Missense**	exon 9	c.1409T>A	p.Leu470Gln
**Missense**	exon 9	c.1411A>C	p.Asn471His
**Missense**	exon 9	c.1415A>C	p.Lys472Thr
**Missense**	exon 9	c.1418A>C	p.Lys473Thr
**Missense**	exon 9	c.1420T>C	p.Ser474Pro
**Splicing**	Intron 6	c.1019-2A>G	N/A
**Splicing**	Intron 7	c.1140-2A>T	N/A
**Regulatory**	#N/A	c.-107G>A	N/A
**Regulatory**	#N/A	c.-63C>A	N/A
**Regulatory**	#N/A	c.-4C>G	N/A
**Regulatory**	#N/A	c.*29G>A	N/A
**Regulatory**	#N/A	c.*63A>G	N/A
**Regulatory**	#N/A	c.*368T>C	N/A
**Regulatory**	#N/A	c.*1660G>A	N/A
**Regulatory**	#N/A	c.*1765G>A	N/A
**Regulatory**	#N/A	c.*1785T>C	N/A
**Regulatory**	#N/A	c.*1793G>A	N/A
**Regulatory**	#N/A	c.*1807G>T	N/A
**Regulatory**	#N/A	c.*1846C>T	N/A
**Regulatory**	#N/A	c.*1869C>T	N/A
**Small Deletion**	exon 3	c.250-8_250-6delATC	N/A
**Small Deletion**	exon 1	c.47_55delAGAGTCTGA	p.Gln16_Thr19delinsPro
**Small Deletion**	exon 3	c.332_334del	p.Val111del
**Small Deletion**	exon 5	c.543_544delCC	p.Leu182Argfs*6
**Small Deletion**	exon 6	c.963delA	p.Ala322Profs*9
**Small Insertion**	#N/A	c.*1806dupT	N/A
**Small Indel**	exon 8	c.1265_1272delGCAGTCCCinsAT	p.Ser422_Pro424delinsAsn

References

1. Pugni, L.; Riva, E.; Pietrasanta, C.; Rabacchi, C.; Bertolini, S.; Pederiva, C.; Mosca, F.; Calandra, S. Severe Hypertriglyceridemia in a Newborn with Monogenic Lipoprotein Lipase Deficiency: An Unconventional Therapeutic Approach with Exchange Transfusion. In *JIMD Reports - Case and Research Reports, Volume 13*; Zschocke, J., Gibson, K.M., Brown, G., Morava, E., Peters, V., Eds.; Springer: Berlin, Heidelberg, 2014; pp. 59–64 ISBN 978-3-642-54149-0.

2. Gotoda, T.; Shirai, K.; Ohta, T.; Kobayashi, J.; Yokoyama, S.; Oikawa, S.; Bujo, H.; Ishibashi, S.; Arai, H.; Yamashita, S.; et al. Diagnosis and Management of Type I and Type V Hyperlipoproteinemia. *Journal of Atherosclerosis and Thrombosis* **2012**, *19*, 1–12, doi:10.5551/jat.10702.

3. Dron, J.S.; Wang, J.; McIntyre, A.D.; Cao, H.; Robinson, J.F.; Duell, P.B.; Manjoo, P.; Feng, J.; Movsesyan, I.; Malloy, M.J.; et al. Partial LPL Deletions: Rare Copy-Number Variants Contributing towards Severe Hypertriglyceridemia. *J Lipid Res* **2019**, *60*, 1953–1958, doi:10.1194/jlr.P119000335.

4. Yang, W.S.; Nevin, D.N.; Peng, R.; Brunzell, J.D.; Deeb, S.S. A Mutation in the Promoter of the Lipoprotein Lipase (LPL) Gene in a Patient with Familial Combined Hyperlipidemia and Low LPL Activity. *Proceedings of the National Academy of Sciences* **1995**, *92*, 4462–4466, doi:10.1073/pnas.92.10.4462.

5. Evans, D.; Arzer, J.; Aberle, J.; Beil, F.U. Rare Variants in the Lipoprotein Lipase (LPL) Gene Are Common in Hypertriglyceridemia but Rare in Type III Hyperlipidemia. *Atherosclerosis* **2011**, *214*, 386–390, doi:10.1016/j.atherosclerosis.2010.11.026.

6. Yu, X.-H.; Zhao, T.-Q.; Wang, L.; Liu, Z.-P.; Zhang, C.-M.; Chen, R.; Li, L.; Liu, G.; Hu, W.-C. A Novel Substitution at the Translation Initiator Codon (ATG-->ATC) of the Lipoprotein Lipase Gene Is Mainly Responsible for Lipoprotein Lipase Deficiency in a Patient with Severe Hypertriglyceridemia and Recurrent Pancreatitis. *Biochem Biophys Res Commun* **2006**, *341*, 82–87, doi:10.1016/j.bbrc.2005.12.165.

7. Wang, S.; Cheng, Y.; Shi, Y.; Zhao, W.; Gao, L.; Fang, L.; Jin, X.; Han, X.; Sun, Q.; Li, G.; et al. Identification and Characterization of Two Novel Compounds: Heterozygous Variants of Lipoprotein Lipase in Two Pedigrees With Type I Hyperlipoproteinemia. *Front Endocrinol (Lausanne)* **2022**, *13*, 874608, doi:10.3389/fendo.2022.874608.

8. Jin, J.-L.; Sun, D.; Cao, Y.-X.; Zhang, H.-W.; Guo, Y.-L.; Wu, N.-Q.; Zhu, C.-G.; Gao, Y.; Dong, Q.-T.; Liu, G.; et al. Intensive Genetic Analysis for Chinese Patients with Very High Triglyceride Levels: Relations of Mutations to Triglyceride Levels and Acute Pancreatitis. *EBioMedicine* **2018**, *38*, 171–177, doi:10.1016/j.ebiom.2018.11.001.

9. Zhang, G.; Hu, Y.; Yang, Q.; Pu, N.; Li, G.; Zhang, J.; Tong, Z.; Masson, E.; Cooper, D.N.; Chen, J.-M.; et al. Frameshift Coding Sequence Variants in the LPL Gene: Identification of Two Novel Events and Exploration of the Genotype–Phenotype Relationship for Variants Reported to Date. *Lipids Health Dis* **2023**, *22*, 128, doi:10.1186/s12944-023-01898-w.

10. Nakamura, T.; Suehiro, T.; Yasuoka, N.; Yamamoto, M.; Ito, H.; Yamano, T.; Hashimoto, K. A Novel Nonsense Mutation in Exon 1 and a Transition in Intron 3 of the Lipoprotein Lipase Gene. *J Atheroscler Thromb* **1996**, *3*, 17–24, doi:10.5551/jat1994.3.17.

11. Li, X.; Yang, Q.; Shi, X.; Chen, W.; Pu, N.; Li, W.; Li, J. Compound but Non-Linked Heterozygous p.W14X and p.L279 V LPL Gene Mutations in a Chinese Patient with Long-Term Severe Hypertriglyceridemia and Recurrent Acute Pancreatitis. *Lipids Health Dis* **2018**, *17*, 144, doi:10.1186/s12944-018-0789-2.

12. Dron, J.S.; Wang, J.; McIntyre, A.D.; Iacocca, M.A.; Robinson, J.F.; Ban, M.R.; Cao, H.; Hegele, R.A. Six Years' Experience with LipidSeq: Clinical and Research Learnings from a Hybrid, Targeted Sequencing Panel for Dyslipidemias. *BMC Med Genomics* **2020**, *13*, 23, doi:10.1186/s12920-020-0669-2.

13. Gill, P.K.; Dron, J.S.; Berberich, A.J.; Wang, J.; McIntyre, A.D.; Cao, H.; Hegele, R.A. Combined Hyperlipidemia Is Genetically Similar to Isolated Hypertriglyceridemia. *Journal of Clinical Lipidology* **2021**, *15*, 79–87, doi:10.1016/j.jacl.2020.11.006.

14. Wang, J.; Cao, H.; Ban, M.R.; Kennedy, B.A.; Zhu, S.; Anand, S.; Yusuf, S.; Pollex, R.L.; Hegele, R.A. Resequencing Genomic DNA of Patients With Severe Hypertriglyceridemia (MIM 144650). *Arteriosclerosis, Thrombosis, and Vascular Biology* **2007**, *27*, 2450–2455, doi:10.1161/ATVBAHA.107.150680.

15. Li, Z.; Zhang, X.; Li, X.; Yang, Y.; Xin, H.; Yang, X.; Liu, N.; Gai, Z.; Liu, Y. A Non-Integrated iPSC Line (SDQLCHi042-A) from a Boy Suffering from Familial Combined Hyperlipidemia with Compound Heterozygous Mutations of Lipoprotein Lipase Gene. *Stem Cell Research* **2021**, *53*, 102313, doi:10.1016/j.scr.2021.102313.

16. Chimienti, G.; Capurso, A.; Resta, F.; Pepe, G. A G→C Change at the Donor Splice Site of Intron 1 Causes Lipoprotein Lipase Deficiency in a Southern-Italian Family. *Biochemical and Biophysical Research Communications* **1992**, *187*, 620–627, doi:10.1016/0006-291X(92)91240-Q.

17. Deshotels, M.R.; Hadley, T.D.; Roth, M.; Agha, A.M.; Pulipati, V.P.; Nugent, A.K.; Virani, S.S.; Nambi, V.; Moriarty, P.M.; Davidson, M.H.; et al. Genetic Testing for Hypertriglyceridemia in Academic Lipid Clinics: Implications for Precision Medicine-Brief Report. *Arterioscler Thromb Vasc Biol* **2022**, *42*, 1461–1467, doi:10.1161/ATVBAHA.122.318445.

18. Rabacchi, C.; Pisciotta, L.; Cefalù, A.B.; Noto, D.; Fresa, R.; Tarugi, P.; Averna, M.; Bertolini, S.; Calandra, S. Spectrum of Mutations of the *LPL* Gene Identified in Italy in Patients with Severe Hypertriglyceridemia. *Atherosclerosis* **2015**, *241*, 79–86, doi:10.1016/j.atherosclerosis.2015.04.815.

19. D'Erasmo, L.; Di Costanzo, A.; Cassandra, F.; Minicocci, I.; Polito, L.; Montali, A.; Ceci, F.; Arca, M. Spectrum of Mutations and Long-Term Clinical Outcomes in Genetic Chylomicronemia Syndromes. *Arteriosclerosis, Thrombosis, and Vascular Biology* **2019**, *39*, 2531–2541, doi:10.1161/ATVBAHA.119.313401.

20. Chokshi, N.; Blumenschein, S.D.; Ahmad, Z.; Garg, A. Genotype-Phenotype Relationships in Patients with Type I Hyperlipoproteinemia. *Journal of Clinical Lipidology* **2014**, *8*, 287–295, doi:10.1016/j.jacl.2014.02.006.

21. Kuthiroly, S.; Yesodharan, D.; Radhakrishnan, N.; Ganapathy, A.; Mannan, A.U.; Hoffmann, M.M.; Nampoothiri, S. Lipoprotein Lipase Deficiency. *Indian J Pediatr* **2021**, *88*, 147–153, doi:10.1007/s12098-020-03305-z.

22. Gupta, N.; Moore, D.; Hooper, A.J.; Burnett, J.R.; Metz, M. Pancreatitis in a Child With Lipemia Due to Novel Lipoprotein Lipase Mutations. *Journal of Pediatric Gastroenterology and Nutrition* **2010**, *50*, 457–459, doi:10.1097/MPG.0b013e3181b64407.

23. Akesson, L.S.; Burnett, J.R.; Mehta, D.K.; Martin, A.C. Lipoprotein Lipase Deficiency Presenting with Neonatal Perianal Abscesses. *Case Reports* **2016**, *2016*, bcr2015212587, doi:10.1136/bcr-2015-212587.

24. Hegele, R.A.; Berberich, A.J.; Ban, M.R.; Wang, J.; Digenio, A.; Alexander, V.J.; D'Erasmo, L.; Arca, M.; Jones, A.; Bruckert, E.; et al. Clinical and Biochemical Features of Different Molecular Etiologies of Familial Chylomicronemia. *Journal of Clinical Lipidology* **2018**, *12*, 920-927.e4, doi:10.1016/j.jacl.2018.03.093.

25. Okubo, M.; Horinishi, A.; Saito, M.; Ebara, T.; Endo, Y.; Kaku, K.; Murase, T.; Eto, M. A Novel Complex Deletion–Insertion Mutation Mediated by *Alu* Repetitive Elements Leads to Lipoprotein Lipase Deficiency. *Molecular Genetics and Metabolism* **2007**, *92*, 229–233, doi:10.1016/j.ymgme.2007.06.018.

26. Hölzl, B.; Kraft, H.G.; Wiebusch, H.; Sandhofer, A.; Patsch, J.; Sandhofer, F.; Paulweber, B. Two Novel Mutations in the Lipoprotein Lipase Gene in a Family with Marked Hypertriglyceridemia in Heterozygous Carriers: Potential Interaction with the Polymorphic Marker D1S104 on Chromosome 1q21–Q23. *Journal of Lipid Research* **2000**, *41*, 734–741, doi:10.1016/S0022-2275(20)32382-8.

27. Hou, Y.-C.C.; Yu, H.-C.; Martin, R.; Cirulli, E.T.; Schenker-Ahmed, N.M.; Hicks, M.; Cohen, I.V.; Jönsson, T.J.; Heister, R.; Napier, L.; et al. Precision Medicine Integrating Whole-Genome Sequencing, Comprehensive Metabolomics, and Advanced Imaging. *Proc Natl Acad Sci U S A* **2020**, *117*, 3053–3062, doi:10.1073/pnas.1909378117.

28. Overgaard, M.; Brasen, C.L.; Svaneby, D.; Feddersen, S.; Nybo, M. Familial Lipoprotein Lipase Deficiency: A Case of Compound Heterozygosity of a Novel Duplication (R44Kfs*4) and a Common Mutation (N291S) in the Lipoprotein Lipase Gene. *Ann Clin Biochem* **2013**, *50*, 374–379, doi:10.1177/0004563213477393.

29. Reina, M.; Brunzell, J.; Deeb, S. Molecular Basis of Familial Chylomicronemia: Mutations in the Lipoprotein Lipase and Apolipoprotein C-II Genes. *Journal of Lipid Research* **1992**, *33*, 1823–1832, doi:10.1016/S0022-2275(20)41340-9.

30. Han, P.; Wei, G.; Cai, K.; Xiang, X.; Deng, W.P.; Li, Y.B.; Kuang, S.; Dong, Z.; Zheng, T.; Luo, Y.; et al. Identification and Functional Characterization of Mutations in LPL Gene Causing Severe Hypertriglyceridaemia and Acute Pancreatitis. *J Cell Mol Med* **2020**, *24*, 1286–1299, doi:10.1111/jcmm.14768.

31. Chan, A.O.; But, W.; Lau, G.T.; Tse, W.; Shek, C. A Novel Nonsense Mutation in the LPL Gene in a Chinese Neonate with Hypertriglyceridemia. *Clinica Chimica Acta* **2006**, *368*, 120–124, doi:10.1016/j.cca.2005.12.020.

32. Benlian, P.; Foubert, L.; Gagné, E.; Bernard, L.; De Gennes, J.L.; Langlois, S.; Robinson, W.; Hayden, M. Complete Paternal Isodisomy for Chromosome 8 Unmasked by Lipoprotein Lipase Deficiency. *Am J Hum Genet* **1996**, *59*, 431–436.

33. Kobayashi, J.; Inadera, H.; Fujita, Y.; Talley, G.; Morisaki, N.; Yoshida, S.; Saito, Y.; Fojo, S.S.; Brewer, H.B. A Naturally Occurring Mutation at the Second Base of Codon Asparagine 43 in the Proposed N-Linked Glycosylation Site of Human Lipoprotein Lipase: *In Vivo* Evidence That Asparagine 43 Is Essential for Catalysis and Secretion. *Biochemical and Biophysical Research Communications* **1994**, *205*, 506–515, doi:10.1006/bbrc.1994.2694.

34. Ben-Zeev, O.; Stahnke, G.; Liu, G.; Davis, R.C.; Doolittle, M.H. Lipoprotein Lipase and Hepatic Lipase: The Role of Asparagine-Linked Glycosylation in the Expression of a Functional Enzyme. *J Lipid Res* **1994**, *35*, 1511–1523.

35. Buscá, R.; Pujana, M.A.; Pognonec, P.; Auwerx, J.; Deeb, S.S.; Reina, M.; Vilaró, S. Absence of N-Glycosylation at Asparagine 43 in Human Lipoprotein Lipase Induces Its Accumulation in the Rough Endoplasmic Reticulum and Alters This Cellular Compartment. *Journal of Lipid Research* **1995**, *36*, 939–951, doi:10.1016/S0022-2275(20)39852-7.

36. Castro-Orós, I.D.; Civeira, F.; Pueyo, M.J.; Mateo-Gallego, R.; Bolado-Carrancio, A.; Lamíquiz-Moneo, I.; Álvarez-Sala, L.; Fabiani, F.; Cofán, M.; Cenarro, A.; et al. Rare Genetic Variants with Large Effect on Triglycerides in Subjects with a Clinical Diagnosis of Familial vs Nonfamilial Hypertriglyceridemia. *Journal of Clinical Lipidology* **2016**, *10*, 790–797, doi:10.1016/j.jacl.2016.02.010.

37. Ariza, M.J.; Pérez-López, C.; Almagro, F.; Sánchez-Tévar, A.M.; Muñiz-Grijalvo, O.; Álvarez-Sala Walter, L.A.; Rioja, J.; Sánchez-Chaparro, M.Á.; Valdivielso, P. Genetic Variants in the *LPL* and *GPIHBP1* Genes, in Patients with Severe Hypertriglyceridaemia, Detected with High Resolution Melting Analysis. *Clinica Chimica Acta* **2020**, *500*, 163–171, doi:10.1016/j.cca.2019.10.011.

38. Stefanutti, C.; Gozzer, M.; Pisciotta, L.; D'Eufemia, P.; Bosco, G.; Morozzi, C.; Papadia, F.; Shafii, M.; Di Giacomo, S.; Bertolini, S. A Three Month-Old Infant with Severe Hyperchylomicronemia: Molecular Diagnosis and Extracorporeal Treatment. *Atherosclerosis Supplements* **2013**, *14*, 73–76, doi:10.1016/j.atherosclerosissup.2012.10.020.

39. Mead, J.R.; Irvine, S.A.; Ramji, D.P. Lipoprotein Lipase: Structure, Function, Regulation, and Role in Disease. *J Mol Med* **2002**, *80*, 753–769, doi:10.1007/s00109-002-0384-9.

40. Hsu, H.; Roncolato, F.; Lee, S.-H. Lysosomal Acid Lipase Deficiency Diagnosed in a Patient Presenting with Acute Myeloid Leukaemia. *British Journal of Haematology* **2021**, *194*, 228–228, doi:10.1111/bjh.17389.

41. Rahalkar, A.R.; Giffen, F.; Har, B.; Ho, J.; Morrison, K.M.; Hill, J.; Wang, J.; Hegele, R.A.; Joy, T. Novel LPL Mutations Associated with Lipoprotein Lipase Deficiency: Two Case Reports and a Literature Review. *Can. J. Physiol. Pharmacol.* **2009**, *87*, 151–160, doi:10.1139/Y09-005.

42. Gotoda, T.; Yamada, N.; Kawamura, M.; Kozaki, K.; Mori, N.; Ishibashi, S.; Shimano, H.; Takaku, F.; Yazaki, Y.; Furuichi, Y. Heterogeneous Mutations in the Human Lipoprotein Lipase Gene in Patients with Familial Lipoprotein Lipase Deficiency. *J Clin Invest* **1991**, *88*, 1856–1864.

43. Hoshi, N.; Jain, A.; Persaud, J.W.; Defesche, J.; Nair, D.R. An Approach to Genetic Diagnosis of Severe Hypertriglyceridaemia. *Atherosclerosis* **2016**, *245*, e244, doi:10.1016/j.atherosclerosis.2015.10.030.

44. Marmontel, O.; Charrière, S.; Simonet, T.; Bonnet, V.; Dumont, S.; Mahl, M.; Jacobs, C.; Nony, S.; Chabane, K.; Bozon, D.; et al. Single, Short in-Del, and Copy Number Variations Detection in Monogenic Dyslipidemia Using a next-Generation Sequencing Strategy. *Clinical Genetics* **2018**, *94*, 132–140, doi:10.1111/cge.13250.

45. Martín-Campos, J.M.; Julve, J.; Roig, R.; Martínez, S.; Errico, T.L.; Martínez-Couselo, S.; Escolà-Gil, J.C.; Méndez-González, J.; Blanco-Vaca, F. Molecular Analysis of Chylomicronemia in a Clinical Laboratory Setting: Diagnosis of 13 Cases of Lipoprotein Lipase Deficiency. *Clinica Chimica Acta* **2014**, *429*, 61–68, doi:10.1016/j.cca.2013.11.025.

46. Langlois, S.; Deeb, S.; Brunzell, J.D.; Kastelein, J.J.; Hayden, M.R. A Major Insertion Accounts for a Significant Proportion of Mutations Underlying Human Lipoprotein Lipase Deficiency. *Proc. Natl. Acad. Sci. U.S.A.* **1989**, *86*, 948–952, doi:10.1073/pnas.86.3.948.

47. Hata, A.; Emi, M.; Luc, G.; Basdevant, A.; Gambert, P.; Iverius, P.H.; Lalouel, J.M. Compound Heterozygote for Lipoprotein Lipase Deficiency: Ser----Thr244 and Transition in 3’ Splice Site of Intron 2 (AG----AA) in the Lipoprotein Lipase Gene. *Am J Hum Genet* **1990**, *47*, 721–726.

48. Tanaka, S.; Ueno, T.; Tsunemi, A.; Nakamura, Y.; Kobayashi, H.; Hatanaka, Y.; Haketa, A.; Fukuda, N.; Soma, M.; Abe, M. Lipoprotein Lipase Deficiency Arising in Type V Dyslipidemia. *Intern Med* **2019**, *58*, 251–257, doi:10.2169/internalmedicine.0952-18.

49. Sprecher, D.L.; Kobayashi, J.; Rymaszewski, M.; Goldberg, I.J.; Harris, B.V.; Bellet, P.S.; Ameis, D.; Yunker, R.L.; Black, D.M.; Stein, E.A. Trp64----Nonsense Mutation in the Lipoprotein Lipase Gene. *J Lipid Res* **1992**, *33*, 859–866.

50. Ooi, E.M.M.; Russell, B.S.; Olson, E.; Sun, S.Z.; Diffenderfer, M.R.; Lichtenstein, A.H.; Keilson, L.; Barrett, P.H.R.; Schaefer, E.J.; Sprecher, D.L. Apolipoprotein B-100 Containing Lipoprotein Metabolism in Subjects with Lipoprotein Lipase Gene Mutations (106/120). *Arterioscler Thromb Vasc Biol* **2012**, *32*, 459–466, doi:10.1161/ATVBAHA.111.238493.

51. Bruin, T.; Tuzgöl, S.; Mulder, W.J.; Van Den Ende, A.E.; Jansen, H.; Hayden, M.R.; Kastelein, J.J. A Compound Heterozygote for Lipoprotein Lipase Deficiency, Val69–>Leu and Gly188–>Glu: Correlation between in Vitro LPL Activity and Clinical Expression. *Journal of Lipid Research* **1994**, *35*, 438–445, doi:10.1016/S0022-2275(20)41188-5.

52. Rodrigues, R.; Artieda, M.; Tejedor, D.; Martínez, A.; Konstantinova, P.; Petry, H.; Meyer, C.; Corzo, D.; Sundgreen, C.; Klor, H.U.; et al. Pathogenic Classification of LPL Gene Variants Reported to Be Associated with LPL Deficiency. *J Clin Lipidol* **2016**, *10*, 394–409, doi:10.1016/j.jacl.2015.12.015.

53. Perkins, B.A.; Caskey, C.T.; Brar, P.; Dec, E.; Karow, D.S.; Kahn, A.M.; Hou, Y.-C.C.; Shah, N.; Boeldt, D.; Coughlin, E.; et al. Precision Medicine Screening Using Whole-Genome Sequencing and Advanced Imaging to Identify Disease Risk in Adults. *Proc Natl Acad Sci U S A* **2018**, *115*, 3686–3691, doi:10.1073/pnas.1706096114.

54. Foubert, L.; De Gennes, J.L.; Lagarde, J.P.; Ehrenborg, E.; Raisonnier, A.; Girardet, J.P.; Hayden, M.R.; Benlian, P. Assessment of French Patients with LPL Deficiency for French Canadian Mutations. *J Med Genet* **1997**, *34*, 672–675.

55. Benlian, P.; Gennes, J.L.D.; Foubert, L.; Zhang, H.; Gagné, S.E.; Hayden, M. Premature Atherosclerosis in Patients with Familial Chylomicronemia Caused by Mutations in the Lipoprotein Lipase Gene. *New England Journal of Medicine* **1996**, *335*, 848–854, doi:10.1056/NEJM199609193351203.

56. Chan, L.Y.S.; Lam, C.-W.; Mak, Y.-T.; Tomlinson, B.; Tsang, M.-W.; Baum, L.; Masarei, J.R.L.; Pang, C.-P. Genotype-Phenotype Studies of Six Novel LPL Mutations in Chinese Patients with Hypertriglyceridemia. *Human Mutation* **2002**, *20*, 232–233, doi:10.1002/humu.9054.

57. Chen, T.-Z.; Xie, S.-L.; Jin, R.; Huang, Z.-M. A Novel Lipoprotein Lipase Gene Missense Mutation in Chinese Patients with Severe Hypertriglyceridemia and Pancreatitis. *Lipids Health Dis* **2014**, *13*, 52, doi:10.1186/1476-511X-13-52.

58. Tomar, S.; Klinzing, D.C.; Chen, C.K.; Gan, L.H.; Moscarello, T.; Reuter, C.; Ashley, E.A.; Foo, R. Causative Variants for Inherited Cardiac Conditions in a Southeast Asian Population Cohort. *Circulation: Genomic and Precision Medicine* **2022**, *15*, e003536, doi:10.1161/CIRCGEN.121.003536.

59. Yang, T.; Lam, C.; Tsang, M.; Chan, L.Y.S.; Poon, P.M.K.; Huang, S.; Pang, C. [Mutation-function analysis in the lipoprotein lipase gene of Chinese patients with hypertriglyceridemic type 2 diabetes]. *Zhongguo Yi Xue Ke Xue Yuan Xue Bao* **2003**, *25*, 134–141.

60. Wilson, D.E.; Hata, A.; Kwong, L.K.; Lingam, A.; Shuhua, J.; Ridinger, D.N.; Yeager, C.; Kaltenborn, K.C.; Iverius, P.H.; Lalouel, J.M. Mutations in Exon 3 of the Lipoprotein Lipase Gene Segregating in a Family with Hypertriglyceridemia, Pancreatitis, and Non-Insulin-Dependent Diabetes. *J Clin Invest* **1993**, *92*, 203–211.

61. Hu, X.; Chen, L.; Gong, C.; Guo, J.; Chen, Y.; Wang, Q.; Guo, R.; Li, W.; Hao, C. Whole Exome Sequencing for Non-Selective Pediatric Patients with Hyperlipidemia. *Gene* **2021**, *768*, 145310, doi:10.1016/j.gene.2020.145310.

62. Ariza, M.J.; Rioja, J.; Ibarretxe, D.; Camacho, A.; Díaz-Díaz, J.L.; Mangas, A.; Carbayo-Herencia, J.A.; Ruiz-Ocaña, P.; Lamíquiz-Moneo, I.; Mosquera, D.; et al. Molecular Basis of the Familial Chylomicronemia Syndrome in Patients from the National Dyslipidemia Registry of the Spanish Atherosclerosis Society. *Journal of Clinical Lipidology* **2018**, *12*, 1482-1492.e3, doi:10.1016/j.jacl.2018.07.013.

63. Ishimura-Oka, K.; Faustinella, F.; Kihara, S.; Smith, L.C.; Oka, K.; Chan, L. A Missense Mutation (Trp86----Arg) in Exon 3 of the Lipoprotein Lipase Gene: A Cause of Familial Chylomicronemia. *Am J Hum Genet* **1992**, *50*, 1275–1280.

64. Thajer, A.; Skacel, G.; de Gier, C.; Greber-Platzer, S. The Effect of a Fat-Restricted Diet in Four Patients with Familial Chylomicronemia Syndrome: A Long-Term Follow-Up Study. *Children (Basel)* **2021**, *8*, 1078, doi:10.3390/children8111078.

65. Caddeo, A.; Mancina, R.M.; Pirazzi, C.; Russo, C.; Sasidharan, K.; Sandstedt, J.; Maurotti, S.; Montalcini, T.; Pujia, A.; Leren, T.P.; et al. Molecular Analysis of Three Known and One Novel LPL Variants in Patients with Type I Hyperlipoproteinemia. *Nutrition, Metabolism and Cardiovascular Diseases* **2018**, *28*, 158–164, doi:10.1016/j.numecd.2017.11.003.

66. Mailly, F.; Palmen, J.; Muller, D.P.R.; Gibbs, T.; Lloyd, J.; Brunzell, J.; Durrington, P.; Mitropoulos, K.; Betteridge, J.; Watts, G.; et al. Familial lipoprotein lipase (LPL) deficiency: A catalogue of LPL gene mutations identified in 20 patients from the UK, Sweden, and Italy. *Human Mutation* **1997**, *10*, 465–473, doi:10.1002/(SICI)1098-1004(1997)10:6<465::AID-HUMU8>3.0.CO;2-C.

67. Jones, A.; Peers, K.; Wierzbicki, A.S.; Ramachandran, R.; Mansfield, M.; Dawson, C.; Ochoa-Ferraro, A.; Soran, H.; Jenkinson, F.; McDowell, I.; et al. Long-Term Effects of Volanesorsen on Triglycerides and Pancreatitis in Patients with Familial Chylomicronaemia Syndrome (FCS) in the UK Early Access to Medicines Scheme (EAMS). *Atherosclerosis* **2023**, *375*, 67–74, doi:10.1016/j.atherosclerosis.2023.05.008.

68. Zhu, L.; Li, G. [A case of neonatal lipoprotein lipase deficiency caused by novel compound heterozygous variants of LPL gene]. *Zhonghua Yi Xue Yi Chuan Xue Za Zhi* **2020**, *37*, 156–158, doi:10.3760/cma.j.issn.1003-9406.2020.02.014.

69. Wu, Y.Q.; Hu, Y.Y.; Li, G.N. Rare Novel LPL Mutations Are Associated with Neonatal Onset Lipoprotein Lipase (LPL) Deficiency in Two Cases. *BMC Pediatr* **2021**, *21*, 414, doi:10.1186/s12887-021-02875-x.

70. Li, X.-Y.; Pu, N.; Chen, W.-W.; Shi, X.-L.; Zhang, G.; Ke, L.; Ye, B.; Tong, Z.-H.; Wang, Y.-H.; Liu, G.; et al. Identification of a Novel LPL Nonsense Variant and Further Insights into the Complex Etiology and Expression of Hypertriglyceridemia-Induced Acute Pancreatitis. *Lipids Health Dis* **2020**, *19*, 63, doi:10.1186/s12944-020-01249-z.

71. Henderson, H.E.; Devlin, R.; Peterson, J.; Brunzell, J.D.; Hayden, M.R. Frameshift Mutation in Exon 3 of the Lipoprotein Lipase Gene Causes a Premature Stop Codon and Lipoprotein Lipase Deficiency. *Mol Biol Med* **1990**, *7*, 511–517.

72. IKEDA, Y.; GOJI, K.; TAKAGI, A. A Compound Heterozygote for a Novel Missense Mutation (G105R) in Exon 3 and a Missense Mutation (D204E) in Exon 5 of the Lipoprotein Lipase Gene in a Japanese Infant with Hyperchylomicronaemia. *Clinical Science* **2000**, *99*, 569–578, doi:10.1042/cs0990569.

73. Emi, M.; Hata, A.; Robertson, M.; Iverius, P.H.; Hegele, R.; Lalouel, J.M. Lipoprotein Lipase Deficiency Resulting from a Nonsense Mutation in Exon 3 of the Lipoprotein Lipase Gene. *Am J Hum Genet* **1990**, *47*, 107–111.

74. Liu, Y.; Lan, Z.; Zhao, F.; Zhang, S.; Zhang, W. Analysis of a Chinese Pedigree With Familial Chylomicronemia Syndrome Reveals Two Novel LPL Mutations by Whole-Exome Sequencing. *Front Genet* **2020**, *11*, 741, doi:10.3389/fgene.2020.00741.

75. Wang, M.; Zhou, Y.; He, X.; Deng, C.; Liu, X.; Li, J.; Zhou, L.; Li, Y.; Zhang, Y.; Liu, H.; et al. Two Novel Mutations of the *LPL* Gene in Two Chinese Family Cases with Familial Chylomicronemia Syndrome. *Clinica Chimica Acta* **2021**, *521*, 264–271, doi:10.1016/j.cca.2021.07.022.

76. Ma, Y.; Liu, M.-S.; Zhang, H.; J.Forsythe, I.; D.Brunzell, J.; R.Hayden, M. A 4 Basepair Deletion in Exon 4 of the Human Lipoprotein Lipase Gene Results in Type I Hyperlipoproteinemia. *Human Molecular Genetics* **1993**, *2*, 1049–1050, doi:10.1093/hmg/2.7.1049.

77. Li, Y.; Hu, M.; Han, L.; Feng, L.; Yang, L.; Chen, X.; Du, T.; Yao, H.; Chen, X. Case Report: Next-Generation Sequencing Identified a Novel Pair of Compound-Heterozygous Mutations of LPL Gene Causing Lipoprotein Lipase Deficiency. *Front Genet* **2022**, *13*, 831133, doi:10.3389/fgene.2022.831133.

78. Hooper, A.J.; Kurtkoti, J.; Hamilton-Craig, I.; Burnett, J.R. Clinical Features and Genetic Analysis of Three Patients with Severe Hypertriglyceridaemia. *Ann Clin Biochem* **2014**, *51*, 485–489, doi:10.1177/0004563214525767.

79. Kolářová, H.; Tesařová, M.; Švecová, Š.; Stránecký, V.; Přistoupilová, A.; Zima, T.; Uhrová, J.; Volgina, S.Y.; Zeman, J.; Honzík, T. Lipoprotein Lipase Deficiency: Clinical, Biochemical and Molecular Characteristics in Three Patients with Novel Mutations in the LPL Gene. *Fol. Biol.* **2014**, *60*, 235–243, doi:10.14712/fb2014060050235.

80. Shah, M.; Roshan, R.; Desai, R.; Kadam, S. Neonatal Hyperlipidemia with Pancreatitis: Novel Gene Mutation of Lipoprotein Lipase. *J Postgrad Med* **2018**, *64*, 247–249, doi:10.4103/jpgm.JPGM_731_17.

81. Surendran, R.P.; Visser, M.E.; Heemelaar, S.; Wang, J.; Peter, J.; Defesche, J.C.; Kuivenhoven, J.A.; Hosseini, M.; Péterfy, M.; Kastelein, J.J.; et al. Mutations in LPL, APOC2, APOA5, GPIHBP1 and LMF1 in Patients with Severe Hypertriglyceridaemia. *J Intern Med* **2012**, *272*, 185–196, doi:10.1111/j.1365-2796.2012.02516.x.

82. Wang, F.; Wang, F.; Zhou, X.; Yi, Y.; Zhao, J. A Novel Lipoprotein Lipase Mutation in an Infant With Glycogen Storage Disease Type-Ib and Severe Hypertriglyceridemia. *Front Pediatr* **2021**, *9*, 671536, doi:10.3389/fped.2021.671536.

83. Bijvoet, S.M.; Bruin, T.; Thzgöl, S.; Bakker, H.D.; Hayden, M.R.; Kastelein, J.J.P. Homozygosity for a Mutation in the Lipoprotein Lipase Gene (Gly139→Ser) Causes Chylomicronaemia in a Boy of Spanish Descent. *Hum Genet* **1994**, *93*, 339–343, doi:10.1007/BF00212035.

84. Ameis, D.; Kobayashi, J.; Davis, R.C.; Ben-Zeev, O.; Malloy, M.J.; Kane, J.P.; Lee, G.; Wong, H.; Havel, R.J.; Schotz, M.C. Familial Chylomicronemia (Type I Hyperlipoproteinemia) Due to a Single Missense Mutation in the Lipoprotein Lipase Gene. *J Clin Invest* **1991**, *87*, 1165–1170.

85. Buscà, R.; Martínez, M.; Vilella, E.; Pognonec, P.; Deeb, S.; Auwerx, J.; Reina, M.; Vilaró, S. The Mutation Gly142→ Glu in Human Lipoprotein Lipase Produces a Missorted Protein That Is Diverted to Lysosomes (∗). *Journal of Biological Chemistry* **1996**, *271*, 2139–2146, doi:10.1074/jbc.271.4.2139.

86. Blackett, P.; Tryggestad, J.; Krishnan, S.; Li, S.; Xu, W.; Alaupovic, P.; Quiroga, C.; Copeland, K. Lipoprotein Abnormalities in Compound Heterozygous Lipoprotein Lipase Deficiency after Treatment with a Low-Fat Diet and Orlistat. *Journal of Clinical Lipidology* **2013**, *7*, 132–139, doi:10.1016/j.jacl.2012.11.006.

87. Bruin, T.; Tuzgöl, S.; Van Diermen, D.E.; Hoogerbrugge-van Der Linden, N.; Brunzell, J.D.; Hayden, M.R.; Kastelein, J.J.P. Recurrent Pancreatitis and Chylomicronemia in an Extended Dutch Kindred Is Caused by a Gly154–>Ser Substitution in Lipoprotein Lipase. *Journal of Lipid Research* **1993**, *34*, 2109–2119, doi:10.1016/S0022-2275(20)35352-9.

88. Ikeda, Y.; Takagi, A.; Nakata, Y.; Sera, Y.; Hyoudou, S.; Hamamoto, K.; Nishi, Y.; Yamamoto, A. Novel Compound Heterozygous Mutations for Lipoprotein Lipase Deficiency: A G-to-T Transversion at the First Position of Exon 5 Causing G154V Missense Mutation and a 5′ Splice Site Mutation of Intron 8. *Journal of Lipid Research* **2001**, *42*, 1072–1081, doi:10.1016/S0022-2275(20)31596-0.

89. Ikeda, Y.; Takagi, A.; Nakata, Y.; Sera, Y.; Hyoudou, S.; Hamamoto, K.; Nishi, Y.; Yamamoto, A. A Family-Based Study of Hyperinsulinemia and Hypertriglyceridemia in Heterozygous Lipoprotein Lipase Deficiency. *Clinica Chimica Acta* **2002**, *316*, 179–185, doi:10.1016/S0009-8981(01)00746-X.

90. Ma, Y.H.; Bruin, T.; Tuzgol, S.; Wilson, B.I.; Roederer, G.; Liu, M.S.; Davignon, J.; Kastelein, J.J.; Brunzell, J.D.; Hayden, M.R. Two Naturally Occurring Mutations at the First and Second Bases of Codon Aspartic Acid 156 in the Proposed Catalytic Triad of Human Lipoprotein Lipase. In Vivo Evidence That Aspartic Acid 156 Is Essential for Catalysis. *Journal of Biological Chemistry* **1992**, *267*, 1918–1923, doi:10.1016/S0021-9258(18)46034-6.

91. Faustinella, F.; Chang, A.; Van Biervliet, J.P.; Rosseneu, M.; Vinaimont, N.; Smith, L.C.; Chen, S.H.; Chan, L. Catalytic Triad Residue Mutation (Asp156—-Gly) Causing Familial Lipoprotein Lipase Deficiency. Co-Inheritance with a Nonsense Mutation (Ser447—-Ter) in a Turkish Family. *Journal of Biological Chemistry* **1991**, *266*, 14418–14424, doi:10.1016/S0021-9258(18)98701-6.

92. Bruin, T.; Kas^TEL^ein, J.J.P.; Van Diermen, D.E.; Ma, Y.; Henderson, H.E.; Stuyt, P.M.J.; Stalenhoef, A.F.H.; Sturk, A.; Brunzell, J.D.; Hayden, M.R. A Missense Mutation Pro157Arg in Lipoprotein Lipase (LPLNijmegen) Resulting in Loss of Catalytic Activity. *European Journal of Biochemistry* **1992**, *208*, 267–272, doi:10.1111/j.1432-1033.1992.tb17182.x.

93. Santer, R.; Gokçay, G.; Demirkol, M.; Gal, A.; Lukacs, Z. Hyperchylomicronaemia Due to Lipoprotein Lipase Deficiency as a Cause of False-Positive Newborn Screening for Biotinidase Deficiency. *Journal of Inherited Metabolic Disease* **2005**, *28*, 137–140, doi:10.1007/s10545-005-7060-5.

94. Wiebusch, H.; Funke, H.; Santer, R.; Richter, W.; Assmann, G. A Novel Missense (E163G) Mutation in the Catalytic Subunit of Lipoprotein Lipase Causes Familial Chylomicronemia. *Hum Mutat* **1996**, *8*, 392, doi:10.1002/humu.1380080402.

95. Guo, D.; Zheng, Y.; Gan, Z.; Guo, Y.; Jiang, S.; Yang, F.; Xiong, F.; Zheng, H. A Heterozygous LMF1 Gene Mutation (c.1523C>T), Combined With an LPL Gene Mutation (c.590G>A), Aggravates the Clinical Symptoms in Hypertriglyceridemia. *Front Genet* **2022**, *13*, 814295, doi:10.3389/fgene.2022.814295.

96. Wright, W.T.; Young, I.S.; Nicholls, D.P.; Graham, C.A. Genetic Screening of the *LPL* Gene in Hypertriglyceridaemic Patients. *Atherosclerosis* **2008**, *199*, 187–192, doi:10.1016/j.atherosclerosis.2007.10.029.

97. Khera, A.V.; Won, H.-H.; Peloso, G.M.; O'Dushlaine, C.; Liu, D.; Stitziel, N.O.; Natarajan, P.; Nomura, A.; Emdin, C.A.; Gupta, N.; et al. Association of Rare and Common Variation in the Lipoprotein Lipase Gene With Coronary Artery Disease. *JAMA* **2017**, *317*, 937–946, doi:10.1001/jama.2017.0972.

98. Brites, F.; Henriksen, F.; Fernández, K.; Brusgaard, K.; Castro, G.; Wikinski, R. New Mutations in the Lipoprotein Lipase Gene in a Young Boy with Chylomicronaemia Syndrome and in His Family. *Acta Paediatrica* **2003**, *92*, 621–624, doi:10.1111/j.1651-2227.2003.tb02517.x.

99. Rip, J.; Nierman, M.C.; Ross, C.J.; Jukema, J.W.; Hayden, M.R.; Kastelein, J.J.P.; Stroes, E.S.G.; Kuivenhoven, J.A. Lipoprotein Lipase S447X: A Naturally Occurring Gain-of-Function Mutation. *Arterioscler Thromb Vasc Biol* **2006**, *26*, 1236–1245, doi:10.1161/01.ATV.0000219283.10832.43.

100. Ma, Y.; Liu, M.S.; Ginzinger, D.; Frohlich, J.; Brunzell, J.D.; Hayden, M.R. Gene-Environment Interaction in the Conversion of a Mild-to-Severe Phenotype in a Patient Homozygous for a Ser172-->Cys Mutation in the Lipoprotein Lipase Gene. *J Clin Invest* **1993**, *91*, 1953–1958.

101. Pruneta-Deloche, V.; Marçais, C.; Perrot, L.; Sassolas, A.; Delay, M.; Estour, B.; Lagarde, M.; Moulin, P. Combination of Circulating Antilipoprotein Lipase (Anti-LPL) Antibody and Heterozygous S172 fsX179 Mutation of LPL Gene Leading to Chronic Hyperchylomicronemia. *The Journal of Clinical Endocrinology & Metabolism* **2005**, *90*, 3995–3998, doi:10.1210/jc.2005-0205.

102. Di Filippo, M.; Marçais, C.; Charrière, S.; Marmontel, O.; Broyer, M.; Delay, M.; Merlin, M.; Nollace, A.; Valéro, R.; Lagarde, M.; et al. Post-Heparin LPL Activity Measurement Using VLDL As a Substrate: A New Robust Method for Routine Assessment of Plasma Triglyceride Lipolysis Defects. *PLoS One* **2014**, *9*, e96482, doi:10.1371/journal.pone.0096482.

103. Birrane, G.; Beigneux, A.P.; Dwyer, B.; Strack-Logue, B.; Kristensen, K.K.; Francone, O.L.; Fong, L.G.; Mertens, H.D.T.; Pan, C.Q.; Ploug, M.; et al. Structure of the Lipoprotein Lipase–GPIHBP1 Complex That Mediates Plasma Triglyceride Hydrolysis. *Proc Natl Acad Sci U S A* **2019**, *116*, 1723–1732, doi:10.1073/pnas.1817984116.

104. Abifadel, M.; Jambart, S.; Allard, D.; Rabès, J.-P.; Varret, M.; Derré, A.; Chouery, E.; Salem, N.; Junien, C.; Aydénian, H.; et al. Identification of the First Lebanese Mutation in the LPL Gene and Description of a Rapid Detection Method. *Clinical Genetics* **2004**, *65*, 158–161, doi:10.1111/j.0009-9163.2004.00205.x.

105. Beg, O.U.; Meng, M.S.; Skarlatos, S.I.; Previato, L.; Brunzell, J.D.; Brewer, H.B.; Fojo, S.S. Lipoprotein lipaseBethesda: A Single Amino Acid Substitution (Ala-176----Thr) Leads to Abnormal Heparin Binding and Loss of Enzymic Activity. *Proc Natl Acad Sci U S A* **1990**, *87*, 3474–3478.

106. Hata, A.; Ridinger, D.N.; Sutherland, S.D.; Emi, M.; Kwong, L.K.; Shuhua, J.; Lubbers, A.; Guy-Grand, B.; Basdevant, A.; Iverius, P.H. Missense Mutations in Exon 5 of the Human Lipoprotein Lipase Gene. Inactivation Correlates with Loss of Dimerization. *Journal of Biological Chemistry* **1992**, *267*, 20132–20139, doi:10.1016/S0021-9258(19)88676-3.

107. Ueda, M.; Burke, F.M.; Remaley, A.T.; Hegele, R.A.; Rader, D.J.; Dunbar, R.L. Familial Chylomicronemia Syndrome With a Novel Homozygous LPL Mutation Identified in Three Siblings in Their 50s. *Ann Intern Med* **2020**, *172*, 500–502, doi:10.7326/L19-0568.

108. Haubenwallner, S.; Hörl, G.; Shachter, N.S.; Presta, E.; Fried, S.K.; Höfler, G.; Kostner, G.M.; Breslow, J.L.; Zechner, R. A Novel Missense Mutation in the Gene for Lipoprotein Lipase Resulting in a Highly Conservative Amino Acid Substitution (Asp180→Glu) Causes Familial Chylomicronemia (Type I Hyperlipoproteinemia). *Genomics* **1993**, *18*, 392–396, doi:10.1006/geno.1993.1481.

109. Marmontel, O.; Rollat-Farnier, P.A.; Wozny, A.-S.; Charrière, S.; Vanhoye, X.; Simonet, T.; Chatron, N.; Collin-Chavagnac, D.; Nony, S.; Dumont, S.; et al. Development of a New Expanded Next-Generation Sequencing Panel for Genetic Diseases Involved in Dyslipidemia. *Clinical Genetics* **2020**, *98*, 589–594, doi:10.1111/cge.13832.

110. Shi, X.; Yang, Q.; Pu, N.; Li, X.; Chen, W.; Zhou, J.; Li, G.; Tong, Z.; Férec, C.; Cooper, D.N.; et al. Identification and Functional Characterization of a Novel Heterozygous Missense Variant in the LPL Associated with Recurrent Hypertriglyceridemia-induced Acute Pancreatitis in Pregnancy. *Mol Genet Genomic Med* **2020**, *8*, e1048, doi:10.1002/mgg3.1048.

111. Tenkanen, H.; Taskinen, M.R.; Antikainen, M.; Ulmanen, I.; Kontula, K.; Ehnholm, C. A Novel Amino Acid Substitution (His183–>Gln) in Exon 5 of the Lipoprotein Lipase Gene Results in Loss of Catalytic Activity: Phenotypic Expression of the Mutant Gene in a Heterozygous State. *Journal of Lipid Research* **1994**, *35*, 220–228, doi:10.1016/S0022-2275(20)41210-6.

112. Emi, M.; Wilson, D.E.; Iverius, P.H.; Wu, L.; Hata, A.; Hegele, R.; Williams, R.R.; Lalouel, J.M. Missense Mutation (Gly----Glu188) of Human Lipoprotein Lipase Imparting Functional Deficiency. *J Biol Chem* **1990**, *265*, 5910–5916.

113. Ishimura-Oka, K.; Semenkovich, C.; Faustinella, F.; Goldberg, I.; Shachter, N.; Smith, L.; Coleman, T.; Hide, W.; Brown, W.; Oka, K. A Missense (Asp250—-Asn) Mutation in the Lipoprotein Lipase Gene in Two Unrelated Families with Familial Lipoprotein Lipase Deficiency. *Journal of Lipid Research* **1992**, *33*, 745–754, doi:10.1016/S0022-2275(20)41438-5.

114. Sagoo, G.S.; Tatt, I.; Salanti, G.; Butterworth, A.S.; Sarwar, N.; van Maarle, M.; Jukema, J.W.; Wiman, B.; Kastelein, J.J.P.; Bennet, A.M.; et al. Seven Lipoprotein Lipase Gene Polymorphisms, Lipid Fractions, and Coronary Disease: A HuGE Association Review and Meta-Analysis. *American Journal of Epidemiology* **2008**, *168*, 1233–1246, doi:10.1093/aje/kwn235.

115. Johansen, C.T.; Dubé, J.B.; Loyzer, M.N.; MacDonald, A.; Carter, D.E.; McIntyre, A.D.; Cao, H.; Wang, J.; Robinson, J.F.; Hegele, R.A. LipidSeq: A next-Generation Clinical Resequencing Panel for Monogenic Dyslipidemias. *J Lipid Res* **2014**, *55*, 765–772, doi:10.1194/jlr.D045963.

116. Dong, W.; Wong, K.H.Y.; Liu, Y.; Levy-Sakin, M.; Hung, W.-C.; Li, M.; Li, B.; Jin, S.C.; Choi, J.; Lopez-Giraldez, F.; et al. Whole-Exome Sequencing Reveals Damaging Gene Variants Associated with Hypoalphalipoproteinemia. *J Lipid Res* **2022**, *63*, 100209, doi:10.1016/j.jlr.2022.100209.

117. Kars, M.E.; Başak, A.N.; Onat, O.E.; Bilguvar, K.; Choi, J.; Itan, Y.; Çağlar, C.; Palvadeau, R.; Casanova, J.-L.; Cooper, D.N.; et al. The Genetic Structure of the Turkish Population Reveals High Levels of Variation and Admixture. *Proc Natl Acad Sci U S A* **2021**, *118*, e2026076118, doi:10.1073/pnas.2026076118.

118. Monsalve, M.V.; Henderson, H.; Roederer, G.; Julien, P.; Deeb, S.; Kastelein, J.J.; Peritz, L.; Devlin, R.; Bruin, T.; Murthy, M.R. A Missense Mutation at Codon 188 of the Human Lipoprotein Lipase Gene Is a Frequent Cause of Lipoprotein Lipase Deficiency in Persons of Different Ancestries. *J Clin Invest* **1990**, *86*, 728–734, doi:10.1172/JCI114769.

119. Henderson, H.E.; Hassan, F.; Berger, G.M.; Hayden, M.R. The Lipoprotein Lipase Gly188----Glu Mutation in South Africans of Indian Descent: Evidence Suggesting Common Origins and an Increased Frequency. *Journal of Medical Genetics* **1992**, *29*, 119–122, doi:10.1136/jmg.29.2.119.

120. Bordugo, A.; Carlin, E.; Demarini, S.; Faletra, F.; Colonna, F. A Neonate with a 'Milky' Blood. What Can It Be? *Archives of Disease in Childhood - Fetal and Neonatal Edition* **2014**, *99*, F514–F514, doi:10.1136/archdischild-2014-305940.

121. Ashraf, A.P.; Hurst, A.C.E.; Garg, A. Extreme Hypertriglyceridemia, Pseudohyponatremia, and Pseudoacidosis in a Neonate with Lipoprotein Lipase Deficiency Due to Segmental Uniparental Disomy. *Journal of Clinical Lipidology* **2017**, *11*, 757–762, doi:10.1016/j.jacl.2017.03.015.

122. Chyzhyk, V.; Kozmic, S.; Brown, A.S.; Hudgins, L.C.; Starc, T.J.; Davila, A.D.; Blevins, T.C.; Diffenderfer, M.R.; He, L.; Geller, A.S.; et al. Extreme Hypertriglyceridemia: Genetic Diversity, Pancreatitis, Pregnancy, and Prevalence. *Journal of Clinical Lipidology* **2019**, *13*, 89–99, doi:10.1016/j.jacl.2018.09.007.

123. Murdock, D.R.; Venner, E.; Muzny, D.M.; Metcalf, G.A.; Murugan, M.; Hadley, T.D.; Chander, V.; de Vries, P.S.; Jia, X.; Hussain, A.; et al. Genetic Testing in Ambulatory Cardiology Clinics Reveals High Rate of Findings with Clinical Management Implications. *Genetics in Medicine* **2021**, *23*, 2404–2414, doi:10.1038/s41436-021-01294-8.

124. Paquette, M.; Amyot, J.; Fantino, M.; Baass, A.; Bernard, S. Rare Variants in Triglycerides-Related Genes Increase Pancreatitis Risk in Multifactorial Chylomicronemia Syndrome. *The Journal of Clinical Endocrinology & Metabolism* **2021**, *106*, e3473–e3482, doi:10.1210/clinem/dgab360.

125. Paquette, C.; Careau, A.-M.; Bergeron, J.; Carpentier, C.; Claveau, J. A Case of Eruptive Xanthomas Associated with Pregnancy Unmasking a G188E Heterozygous Mutation of the Lipoprotein Lipase Gene: A Case Report. *SAGE Open Medical Case Reports* **2022**, *10*, 2050313X221131865, doi:10.1177/2050313X221131865.

126. Valenzuela-Vallejo, L.; Meléndrez-Vásquez, D.; Durán-Ventura, P.; Rivera-Nieto, C.; Lema, A.; Fernandez, M. Severe Hypertriglyceridemia as a Cause of Necrotizing Pancreatitis in a Pediatric Patient with Familial Hyperchylomicronemia Syndrome: A Case Report. *SAGE Open Medical Case Reports* **2022**, *10*, 2050313X221109972, doi:10.1177/2050313X221109972.

127. Zhang, Q.; Cavallero, E.; Hoffmann, M.M.; Cavanna, J.; Kay, A.; Charles, A.; Braschi, S.; Marz, W.; Perlemuter, L.; Jacotot, B.; et al. Mutations at the Lipoprotein Lipase Gene Locus in Subjects with Diabetes Mellitus, Obesity and Lipaemia. *Clinical Science* **1997**, *93*, 335–341, doi:10.1042/cs0930335.

128. Dichek, H.L.; Fojo, S.S.; Beg, O.U.; Skarlatos, S.I.; Brunzell, J.D.; Cutler, G.B.; Brewer, H.B. Identification of Two Separate Allelic Mutations in the Lipoprotein Lipase Gene of a Patient with the Familial Hyperchylomicronemia Syndrome. *Journal of Biological Chemistry* **1991**, *266*, 473–477, doi:10.1016/S0021-9258(18)52459-5.

129. Peterson, J.; Ayyobi, A.F.; Ma, Y.; Henderson, H.; Reina, M.; Deeb, S.S.; Santamarina-Fojo, S.; Hayden, M.R.; Brunzell, J.D. Structural and Functional Consequences of Missense Mutations in Exon 5 of the Lipoprotein Lipase Gene. *Journal of Lipid Research* **2002**, *43*, 398–406, doi:10.1016/S0022-2275(20)30146-2.

130. Henderson, H.E.; Ma, Y.; Hassan, M.F.; Monsalve, M.V.; Marais, A.D.; Winkler, F.; Gubernator, K.; Peterson, J.; Brunzell, J.D.; Hayden, M.R. Amino Acid Substitution (Ile194----Thr) in Exon 5 of the Lipoprotein Lipase Gene Causes Lipoprotein Lipase Deficiency in Three Unrelated Probands. Support for a Multicentric Origin. *J Clin Invest* **1991**, *87*, 2005–2011.

131. Tada, H.; Nomura, A.; Okada, H.; Nakahashi, T.; Nozue, T.; Hayashi, K.; Nohara, A.; Yagi, K.; Inazu, A.; Michishita, I.; et al. Clinical Whole Exome Sequencing in Severe Hypertriglyceridemia. *Clinica Chimica Acta* **2019**, *488*, 31–39, doi:10.1016/j.cca.2018.10.041.

132. Minamizuka, T.; Kobayashi, J.; Tada, H.; Koshizaka, M.; Maezawa, Y.; Yokote, K. Homozygous Familial Lipoprotein Lipase Deficiency without Obvious Coronary Artery Stenosis. *Clinical Biochemistry* **2022**, *108*, 42–45, doi:10.1016/j.clinbiochem.2022.07.001.

133. Ayoub, C.; Azar, Y.; Maddah, D.; Ghaleb, Y.; Elbitar, S.; Abou-Khalil, Y.; Jambart, S.; Varret, M.; Boileau, C.; El Khoury, P.; et al. Low Circulating PCSK9 Levels in LPL Homozygous Children with Chylomicronemia Syndrome in a Syrian Refugee Family in Lebanon. *Front Genet* **2022**, *13*, 961028, doi:10.3389/fgene.2022.961028.

134. Maruyama, T.; Yamashita, S.; Matsuzawa, Y.; Bujo, H.; Takahashi, K.; Saito, Y.; Ishibashi, S.; Ohashi, K.; Shionoiri, F.; Gotoda, T.; et al. Mutations in Japanese Subjects with Primary Hyperlipidemia— Results from the Research Committee of the Ministry of Health and Welfare of Japan since 1996 —. *Journal of Atherosclerosis and Thrombosis* **2004**, *11*, 131–145, doi:10.5551/jat.11.131.

135. Teramoto, R.; Tada, H.; Kawashiri, M.; Nohara, A.; Nakahashi, T.; Konno, T.; Inazu, A.; Mabuchi, H.; Yamagishi, M.; Hayashi, K. Molecular and Functional Characterization of Familial Chylomicronemia Syndrome. *Atherosclerosis* **2018**, *269*, 272–278, doi:10.1016/j.atherosclerosis.2017.11.006.

136. Al-Waili, K.; Al-Rasadi, K.; Al-Bulushi, M.; Habais, M.; Al-Mujaini, A.; Al-Yaarubi, S.; Rimbert, A.; Zadjali, R.; Khaniabadi, P.M.; Al-Barwani, H.; et al. The Genetic Spectrum of Familial Hypertriglyceridemia in Oman. *Front Genet* **2022**, *13*, 886182, doi:10.3389/fgene.2022.886182.

137. Ma, Y.; Henderson, H.E.; Murthy, M.R.V.; Roederer, G.; Monsalve, M.V.; Clarke, L.A.; Normand, T.; Julien, P.; Gagné, C.; Lambert, M.; et al. A Mutation in the Human Lipoprotein Lipase Gene as the Most Common Cause of Familial Chylomicronemia in French Canadians. *New England Journal of Medicine* **1991**, *324*, 1761–1766, doi:10.1056/NEJM199106203242502.

138. Yang, Y.; Mu, Y.; Zhao, Y.; Liu, X.; Zhao, L.; Wang, J.; Xie, Y. Genetic Screening of the Lipoprotein Lipase Gene for Mutations in Chinese Subjects with or without Hypertriglyceridemia. *Journal of Genetics and Genomics* **2007**, *34*, 381–391, doi:10.1016/S1673-8527(07)60041-1.

139. Normand, T.; Bergeron, J.; Fernandez-Margallo, T.; Bharucha, A.; Ven Murthy, M.R.; Julien, P.; Gagné, C.; Dionne, C.; De Braekeleer, M.; Ma, R.; et al. Geographic Distribution and Genealogy of Mutation 207 of the Lipoprotein Lipase Gene in the French Canadian Population of Québec. *Hum Genet* **1992**, *89*, 671–675, doi:10.1007/BF00221960.

140. Bijvoet, S.; Gagné, S.E.; Moorjani, S.; Gagné, C.; Henderson, H.E.; Fruchart, J.C.; Dallongeville, J.; Alaupovic, P.; Prins, M.; Kastelein, J.J.; et al. Alterations in Plasma Lipoproteins and Apolipoproteins before the Age of 40 in Heterozygotes for Lipoprotein Lipase Deficiency. *Journal of Lipid Research* **1996**, *37*, 640–650, doi:10.1016/S0022-2275(20)37605-7.

141. Sacks, F.M.; Stanesa, M.; Hegele, R.A. Severe Hypertriglyceridemia With Pancreatitis: Thirteen Years' Treatment With Lomitapide. *JAMA Internal Medicine* **2014**, *174*, 443–447, doi:10.1001/jamainternmed.2013.13309.

142. Zhang, T.; Joubert, P.; Ansari-Pour, N.; Zhao, W.; Hoang, P.H.; Lokanga, R.; Moye, A.L.; Rosenbaum, J.; Gonzalez-Perez, A.; Martínez-Jiménez, F.; et al. Genomic and Evolutionary Classification of Lung Cancer in Never Smokers. *Nat Genet* **2021**, *53*, 1348–1359, doi:10.1038/s41588-021-00920-0.

143. Murthy, V.; Julien, P.; Gagné, C. Molecular Pathobiology of the Human Lipoprotein Lipase Gene. *Pharmacology & Therapeutics* **1996**, *70*, 101–135, doi:10.1016/0163-7258(96)00005-8.

144. Soto, A.G.; McIntyre, A.; Agrawal, S.; Bialo, S.R.; Hegele, R.A.; Boney, C.M. Severe Hypertriglyceridemia Due to a Novel p.Q240H Mutation in the Lipoprotein Lipase Gene. *Lipids Health Dis* **2015**, *14*, 102, doi:10.1186/s12944-015-0107-1.

145. Takagi, A.; Ikeda, Y.; Tsutsumi, Z.; Shoji, T.; Yamamoto, A. Molecular Studies on Primary Lipoprotein Lipase (LPL) Deficiency. One Base Deletion (G916) in Exon 5 of LPL Gene Causes No Detectable LPL Protein Due to the Absence of LPL mRNA Transcript. *J Clin Invest* **1992**, *89*, 581–591.

146. McLean, A.G.; Petersons, C.J.; Hooper, A.J.; Burnett, J.R.; Burt, M.G.; Doogue, M.P. Extreme Diabetic Lipaemia Associated with a Novel Lipoprotein Lipase Gene Mutation. *Clinica Chimica Acta* **2009**, *406*, 167–169, doi:10.1016/j.cca.2009.05.003.

147. Henderson, H.E.; Ma, Y.; Liu, M.S.; Clark-Lewis, I.; Maeder, D.L.; Kastelein, J.J.; Brunzell, J.D.; Hayden, M.R. Structure-Function Relationships of Lipoprotein Lipase: Mutation Analysis and Mutagenesis of the Loop Region. *Journal of Lipid Research* **1993**, *34*, 1593–1602, doi:10.1016/S0022-2275(20)36952-2.

148. Yang, Q.; Pu, N.; Li, X.-Y.; Shi, X.-L.; Chen, W.-W.; Zhang, G.-F.; Hu, Y.-P.; Zhou, J.; Chen, F.-X.; Li, B.-Q.; et al. Digenic Inheritance and Gene-Environment Interaction in a Patient With Hypertriglyceridemia and Acute Pancreatitis. *Front Genet* **2021**, *12*, 640859, doi:10.3389/fgene.2021.640859.

149. Pingitore, P.; Lepore, S.M.; Pirazzi, C.; Mancina, R.M.; Motta, B.M.; Valenti, L.; Berge, K.E.; Retterstøl, K.; Leren, T.P.; Wiklund, O.; et al. Identification and Characterization of Two Novel Mutations in the LPL Gene Causing Type I Hyperlipoproteinemia. *Journal of Clinical Lipidology* **2016**, *10*, 816–823, doi:10.1016/j.jacl.2016.02.015.

150. Laurie, A.D.; Kyle, C.V. A Novel Frameshift Mutation in the Lipoprotein Lipase Gene Is Rescued by Alternative Messenger RNA Splicing. *Journal of Clinical Lipidology* **2017**, *11*, 357–361, doi:10.1016/j.jacl.2017.01.013.

151. Cheema, H.; Bertoli-Avella, A.M.; Skrahina, V.; Anjum, M.N.; Waheed, N.; Saeed, A.; Beetz, C.; Perez-Lopez, J.; Rocha, M.E.; Alawbathani, S.; et al. Genomic Testing in 1019 Individuals from 349 Pakistani Families Results in High Diagnostic Yield and Clinical Utility. *NPJ Genom Med* **2020**, *5*, 44, doi:10.1038/s41525-020-00150-z.

152. Takagi, A.; Ikeda, Y.; Mori, A.; Tsutsumi, Z.; Oida, K.; Nakai, T.; Yamamoto, A. A Newly Identified Heterozygous Lipoprotein Lipase Gene Mutation (Cys239–>stop/TGC972–>TGA; LPLobama) in a Patient with Primary Type IV Hyperlipoproteinemia. *Journal of Lipid Research* **1994**, *35*, 2008–2018, doi:10.1016/S0022-2275(20)39947-8.

153. Hoffmann, M.M.; Jacob, S.; Luft, D.; Schmülling, R.-M.; Rett, K.; März, W.; Häring, H.-U.; Matthaei, S. Type I Hyperlipoproteinemia Due to a Novel Loss of Function Mutation of Lipoprotein Lipase, Cys239→Trp, Associated with Recurrent Severe Pancreatitis. *The Journal of Clinical Endocrinology & Metabolism* **2000**, *85*, 4795–4798, doi:10.1210/jcem.85.12.7069.

154. Vidanapathirana, D.M.; Rodrigo, T.; Waidyanatha, S.; Jasinge, E.; Hooper, A.J.; Burnett, J.R. Lipoprotein Lipase Deficiency in an Infant With Chylomicronemia, Hepatomegaly, and Lipemia Retinalis. *Glob Pediatr Health* **2017**, *4*, 2333794X17715839, doi:10.1177/2333794X17715839.

155. Shen, J.; Chen, R.; Hu, W.; Bingshen, K.E.; Li, L.; Du, Y.; Liu, Y. [A gene analysis of familial lipoprotein lipase deficiency in China]. *Zhonghua Yi Xue Yi Chuan Xue Za Zhi* **1999**, *16*, 233–235.

156. Liu, Y.; Lun, Y.; Lv, W.; Hou, X.; Wang, Y. A Chinese Patient with Recurrent Pancreatitis during Pregnancy Induced by Hypertriglyceridemia Associated with Compound Heterozygosity (Glu242Lys and Leu252VaL) in the Lipoprotein Lipase Gene. *Journal of Clinical Lipidology* **2016**, *10*, 199-203.e1, doi:10.1016/j.jacl.2015.09.010.

157. Ma, Y.; Liu, M.-S.; Chitayat, D.; Bruin, T.; Beisiegel, U.; Benlian, P.; Foubert, L.; De Gennes, J.L.; Funke, H.; Forsythe, I.; et al. Recurrent Missense Mutations at the First and Second Base of Codon Arg243 in Human Lipoprotein Lipase in Patients of Different Ancestries. *Human Mutation* **1994**, *3*, 52–58, doi:10.1002/humu.1380030109.

158. Plengpanich, W.; Kiateprungvej, A.; Charoen, S.; Khovidhunkit, W. Clinical and Functional Studies of Two Novel Variants in the *LPL* Gene in Subjects with Severe Hypertriglyceridemia. *Clinica Chimica Acta* **2018**, *487*, 22–27, doi:10.1016/j.cca.2018.08.041.

159. Khovidhunkit, W.; Charoen, S.; Kiateprungvej, A.; Chartyingcharoen, P.; Muanpetch, S.; Plengpanich, W. Rare and Common Variants in *LPL* and *APOA5* in Thai Subjects with Severe Hypertriglyceridemia: A Resequencing Approach. *Journal of Clinical Lipidology* **2016**, *10*, 505-511.e1, doi:10.1016/j.jacl.2015.11.007.

160. Thowfeek Zeenath Thaneefa, M.; Amarakoon, G.; Mendis, D.; Jasinge, E.; Hooper, A.J.; Burnett, J.R. Incidental Diagnosis of LPL Deficiency in an Infant Presenting with an Acute Respiratory Infection. *Clinica Chimica Acta* **2022**, *529*, 1–3, doi:10.1016/j.cca.2022.01.016.

161. Sirisena, N.D.; Neththikumara, N.; Wetthasinghe, K.; Dissanayake, V.H.W. Implementation of Genomic Medicine in Sri Lanka: Initial Experience and Challenges. *Applied & Translational Genomics* **2016**, *9*, 33–36, doi:10.1016/j.atg.2016.05.003.

162. Behar, D.M.; Adler, L.; Basel-Vanagaite, L. Severe Hypertriglyceridemia in an Infant of Arab Descent. *Isr Med Assoc J* **2013**, *15*, 53–54.

163. Zhang, Y.; Zhou, J.; Zheng, W.; Lan, Z.; Huang, Z.; Yang, Q.; Liu, C.; Gao, R.; Zhang, Y. Clinical, Biochemical and Molecular Analysis of Two Infants with Familial Chylomicronemia Syndrome. *Lipids Health Dis* **2016**, *15*, 88, doi:10.1186/s12944-016-0254-z.

164. Ma, Y.; Wilson, B.I.; Bijvoet, S.; Henderson, H.E.; Cramb, E.; Roederer, G.; Ven Murthy, M.R.; Julien, P.; Bakker, H.D.; Kastelein, J.J.P.; et al. A Missense Mutation (Asp250→Asn) in Exon 6 of the Human Lipoprotein Lipase Gene Causes Chylomicronemia in Patients of Different Ancestries. *Genomics* **1992**, *13*, 649–653, doi:10.1016/0888-7543(92)90136-G.

165. Bijvoet, S.M.; Wiebusch, H.; Ma, Y.; Reymer, P.W.A.; Bruin, T.; Bakker, H.D.; Funke, H.; Assmann, G.; Hayden, M.R.; Kastelein, J.J.P. Compound Heterozygosity for a Known and a Novel Defect in the Lipoprotein Lipase Gene (Asp250 → Asn; Ser251 → Cys) Resulting in Lipoprotein Lipase (LPL) Deficiency. *The Netherlands Journal of Medicine* **1996**, *49*, 189–195, doi:10.1016/0300-2977(96)00043-5.

166. Siu-ying, N. Familial Lipoprotein Lipase Deficiency – Neonatal Presentation. *Int J Pediatr Endocrinol* **2015**, *2015*, P77, doi:10.1186/1687-9856-2015-S1-P77.

167. Kao, J.T.; Hsiao, W.H.; Yu, C.J.; Chiang, F.T. Newly Identified Missense Mutation Reduces Lipoprotein Lipase Activity in Taiwanese Patients with Hypertriglyceridemia. *J Formos Med Assoc* **1999**, *98*, 606–612.

168. Ma, Y.; Ooi, T.C.; Liu, M.S.; Zhang, H.; McPherson, R.; Edwards, A.L.; Forsythe, I.J.; Frohlich, J.; Brunzell, J.D.; Hayden, M.R. High Frequency of Mutations in the Human Lipoprotein Lipase Gene in Pregnancy-Induced Chylomicronemia: Possible Association with Apolipoprotein E2 Isoform. *Journal of Lipid Research* **1994**, *35*, 1066–1075, doi:10.1016/S0022-2275(20)40102-6.

169. Qin, Y.; Wei, A.; Shan, Q.; Xian, X.; Wu, Y.; Liao, L.; Yan, J.; Lai, Z.; Lin, F. Rare LPL Gene Missense Mutation in an Infant with Hypertriglyceridemia. *Journal of Clinical Laboratory Analysis* **2018**, *32*, e22414, doi:10.1002/jcla.22414.

170. Yang, L.; Wei, Z.; Chen, X.; Hu, L.; Peng, X.; Wang, J.; Lu, C.; Kong, Y.; Dong, X.; Ni, Q.; et al. Use of Medical Exome Sequencing for Identification of Underlying Genetic Defects in NICU: Experience in a Cohort of 2303 Neonates in China. *Clinical Genetics* **2022**, *101*, 101–109, doi:10.1111/cge.14075.

171. Evans, D.; Wendt, D.; Ahle, S.; Guerra, A.; Beisiegel, U. Compound Heterozygosity for a New (S259G) and a Previously Described (G188E) Mutation in Lipoprotein Lipase (LpL) as a Cause of Chylomicronemia. Mutations in Brief No. 183. Online. *Hum Mutat* **1998**, *12*, 217.

172. Foubert, L.; Bruin, T.; Gennes, J.L.D.; Ehrenborg, E.; Furioli, J.; Kastelein, J.; Benlian, P.; Hayden, M. A Single Ser259Arg Mutation in the Gene for Lipoprotein Lipase Causes Chylomicronemia in Moroccans of Berber Ancestry. *Human Mutation* **1997**, *10*, 179–185, doi:10.1002/(SICI)1098-1004(1997)10:3<179::AID-HUMU1>3.0.CO;2-E.

173. Bouabdellah, M.; Iraqi, H.; Benlian, P.; Berqia, I.; Benchekroun, L.; Chraïbi, A.; Chabraoui, L. [Familial hypertriglyceridemia: biochemical, clinical and molecular study in a Moroccan family]. *Ann Biol Clin (Paris)* **2015**, *73*, 474–484, doi:10.1684/abc.2015.1058.

174. Hu, Y.; Zhang, G.; Yang, Q.; Pu, N.; Li, K.; Li, B.; Cooper, D.N.; Tong, Z.; Li, W.; Chen, J.-M. The East Asian-Specific LPL p.Ala288Thr (c.862G > A) Missense Variant Exerts a Mild Effect on Protein Function. *Lipids Health Dis* **2023**, *22*, 119, doi:10.1186/s12944-023-01875-3.

175. Rouis, M.; Lohse, P.; Dugi, K.A.; Lohse, P.; Beg, O.U.; Ronan, R.; Talley, G.D.; Brunzell, J.D.; Santamarina-Fojo, S. Homozygosity for Two Point Mutations in the Lipoprotein Lipase (LPL) Gene in a Patient with Familial LPL Deficiency: LPL(Asp9–>Asn, Tyr262–>His). *Journal of Lipid Research* **1996**, *37*, 651–661, doi:10.1016/S0022-2275(20)37606-9.

176. Murugasu, C.G.; Armstrong, G.; Creedon, G.; Cavanna, J.S.; Galton, D.J.; Tomkin, G.H. Acute Hypertriglyceridaemic Pancreatitis in a Pregnant Indian: A New Lipoprotein Lipase Gene Mutation. *J R Soc Med* **1998**, *91*, 205–207.

177. Takagi, A.; Ikeda, Y.; Takeda, E.; Yamamoto, A. A Newly Identified Lipoprotein Lipase (LPL) Gene Mutation (F270L) in a Japanese Patient with Familial LPL Deficiency. *Biochimica et Biophysica Acta (BBA) - Molecular Basis of Disease* **2000**, *1502*, 433–446, doi:10.1016/S0925-4439(00)00067-3.

178. Stranneheim, H.; Lagerstedt-Robinson, K.; Magnusson, M.; Kvarnung, M.; Nilsson, D.; Lesko, N.; Engvall, M.; Anderlid, B.-M.; Arnell, H.; Johansson, C.B.; et al. Integration of Whole Genome Sequencing into a Healthcare Setting: High Diagnostic Rates across Multiple Clinical Entities in 3219 Rare Disease Patients. *Genome Med* **2021**, *13*, 40, doi:10.1186/s13073-021-00855-5.

179. Devlin, R.H.; Deeb, S.; Brunzell, J.; Hayden, M.R. Partial Gene Duplication Involving Exon-Alu Interchange Results in Lipoprotein Lipase Deficiency. *Am J Hum Genet* **1990**, *46*, 112–119.

180. Ayyavoo, A.; Raghupathy, P.; Agarwal, M.; Hofman, P. Severe Familial Hypertriglyceridemia: Successful Treatment With Insulin and a Modified Meal Plan. *J Endocr Soc* **2018**, *2*, 1357–1362, doi:10.1210/js.2018-00299.

181. Johansen, C.T.; Wang, J.; Lanktree, M.B.; Cao, H.; McIntyre, A.D.; Ban, M.R.; Martins, R.A.; Kennedy, B.A.; Hassell, R.G.; Visser, M.E.; et al. Excess of Rare Variants in Genes Identified by Genome-Wide Association Study of Hypertriglyceridemia. *Nat Genet* **2010**, *42*, 684–687, doi:10.1038/ng.628.

182. Saika, Y.; Sakai, N.; Takahashi, M.; Maruyama, T.; Kihara, S.; Ouchi, N.; Ishigami, M.; Hiraoka, H.; Nakamura, T.; Yamashita, S.; et al. Novel LPL Mutation (L303F) Found in a Patient Associated with Coronary Artery Disease and Severe Systemic Atherosclerosis. *European Journal of Clinical Investigation* **2003**, *33*, 216–222, doi:10.1046/j.1365-2362.2003.01129.x.

183. Takagi, A.; Ikeda, Y.; Tsushima, M.; Yamamoto, A. 1.P.54 Molecular and Environmental Bases of Primary Type IV Hyperlipoproteinemia: Heterozygous Lipoprotein Lipase Deficiency as a Causal Genetic Disorder. *Atherosclerosis* **1997**, *134*, 27–28, doi:10.1016/S0021-9150(97)88233-0.

184. Lee, J.; Ko, K.; Ryu, S.; Lee, J.; Lee, K.; Son, C.; Lee, J. Compound Heterozygosity of Novel Missense Mutations (Ser45 → Gly, Cys278 → Arg) in the Lipoprotein Lipase Gene in a Newborn Korean Infant. *Clinica Chimica Acta* **2008**, *387*, 172–174, doi:10.1016/j.cca.2007.08.007.

185. Lun, Y.; Sun, X.; Wang, P.; Chi, J.; Hou, X.; Wang, Y. Severe Hypertriglyceridemia Due to Two Novel Loss-of-Function Lipoprotein Lipase Gene Mutations (C310R/E396V) in a Chinese Family Associated with Recurrent Acute Pancreatitis. *Oncotarget* **2017**, *8*, 47741–47754, doi:10.18632/oncotarget.17762.

186. Yue, X.-Y.; Sun, X.-F.; Che, K.; Hu, J.-X.; Lv, W.-S.; Sun, X.-L.; Geng, Z.; Chi, J.-W.; Wang, Y.-G. *Lpl-C310R* Mutation Is Associated with Impaired Glucose Tolerance and Endoplasmic Reticulum Stress in Skeletal Muscle. *Biochemical and Biophysical Research Communications* **2020**, *529*, 480–486, doi:10.1016/j.bbrc.2020.06.055.

187. Shin, J.-M.; Kim, K.H.; Kim, S.-M.; Hong, D.; Park, J.; Lee, H.-Y.; Lim, W.-J.; Shin, Y.-A.; Kim, C.-D.; Seo, Y.-J.; et al. Exome Sequencing Reveals Novel Candidate Gene Variants Associated with Clinical Characteristics in Alopecia Areata Patients. *Journal of Dermatological Science* **2020**, *99*, 216–220, doi:10.1016/j.jdermsci.2020.08.003.

188. Causeret, A.S.; Souillet, A.L.; Marcais, C.; Prunetta, V.; Lachaux, A.; Faure, M.; Claudy, A. [Familial hyperchylomicronemia with a new mutation of the lipoprotein lipase gene]. *Ann Dermatol Venereol* **2001**, *128*, 1343–1345.

189. Kobayashi, J.; Nagashima, I.; Taira, K.; Hikita, M.; Tamura, K.; Bujo, H.; Morisaki, N.; Saito, Y. A Novel Frameshift Mutation in Exon 6 (the Site of Asn 291) of the Lipoprotein Lipase Gene in Type I Hyperlipidemia. *Clinica Chimica Acta* **1999**, *285*, 173–182, doi:10.1016/S0009-8981(99)00060-1.

190. Kavazarakis, E.; Stabouli, S.; Gourgiotis, D.; Roumeliotou, K.; Traeger-Synodinos, J.; Bossios, A.; Fretzayas, A.; Kanavakis, E. Severe Hypertriglyceridaemia in a Greek Infant: A Clinical, Biochemical and Genetic Study. *Eur J Pediatr* **2004**, *163*, 462–466, doi:10.1007/s00431-004-1474-1.

191. Vigna, G.B.; Citroni, N.; Tarugi, P.; Fellin, R. Familial Chylomicronemia Syndrome. A Sixty Year Follow-up in Two Siblings and Their Kindreds. Nosological and Clinical Considerations. *Journal of Clinical Lipidology* **2022**, *16*, 591–595, doi:10.1016/j.jacl.2022.07.013.

192. Feng, L.; Sun, Y.; Liu, F.; Wang, C.; Zhang, C.; Liu, J.; Jiang, L. Clinical Features and Functions of a Novel Lpl Mutation C.986A>C (p.Y329S) in Patient with Hypertriglyceridemia. *Current Research in Translational Medicine* **2022**, *70*, 103337, doi:10.1016/j.retram.2022.103337.

193. Bertolini, S.; Simone, M.; Pes, G.; Ghisellini, M.; Rolleri, M.; Bellocchio, A.; Elicio, N.; Masturzo, P.; Calandra, S. Pseudodominance of Lipoprotein Lipase (LPL) Deficiency Due to a Nonsense Mutation (Tyr302>Term) in Exon 6 of LPL Gene in an Italian Family from Sardinia (LPLOlbia). *Clinical Genetics* **2000**, *57*, 140–147, doi:10.1034/j.1399-0004.2000.570209.x.

194. Monies, D.; Abouelhoda, M.; Assoum, M.; Moghrabi, N.; Rafiullah, R.; Almontashiri, N.; Alowain, M.; Alzaidan, H.; Alsayed, M.; Subhani, S.; et al. Lessons Learned from Large-Scale, First-Tier Clinical Exome Sequencing in a Highly Consanguineous Population. *The American Journal of Human Genetics* **2019**, *104*, 1182–1201, doi:10.1016/j.ajhg.2019.04.011.

195. Hu, Y.; Ren, Y.; Luo, R.Z.; Mao, X.; Li, X.; Cao, X.; Guan, L.; Chen, X.; Li, J.; Long, Y.; et al. Novel Mutations of the Lipoprotein Lipase Gene Associated with Hypertriglyceridemia in Members of Type 2 Diabetic Pedigrees S.□ *Journal of Lipid Research* **2007**, *48*, 1681–1688, doi:10.1194/jlr.M600382-JLR200.

196. Lamiquiz-Moneo, I.; Blanco-Torrecilla, C.; Bea, A.M.; Mateo-Gallego, R.; Pérez-Calahorra, S.; Baila-Rueda, L.; Cenarro, A.; Civeira, F.; de Castro-Orós, I. Frequency of Rare Mutations and Common Genetic Variations in Severe Hypertriglyceridemia in the General Population of Spain. *Lipids Health Dis* **2016**, *15*, 82, doi:10.1186/s12944-016-0251-2.

197. Hölzl, B.; Huber, R.; Paulweber, B.; Patsch, J.R.; Sandhofer, F. Lipoprotein Lipase Deficiency Due to a 3’ Splice Site Mutation in Intron 6 of the Lipoprotein Lipase Gene. *Journal of Lipid Research* **1994**, *35*, 2161–2169, doi:10.1016/S0022-2275(20)39922-3.

198. Kobayashi, J.; Sasaki, N.; Tashiro, J.; Inadera, H.; Saito, Y.; Yoshida, S. A Missense Mutation (Ala334⇒Thr) in Exon 7 of the Lipoprotein Lipase Gene in a Case with Type I Hyperlipidemia. *Biochemical and Biophysical Research Communications* **1993**, *191*, 1046–1054, doi:10.1006/bbrc.1993.1323.

199. Avis, H.; Scheffer, H.; Kastelein, J.; Dallinga-Thie, G.; Wijburg, F. Pink-Creamy Whole Blood in a 3-Month-Old Infant with a Homozygous Deletion in the Lipoprotein Lipase Gene. *Clinical Genetics* **2010**, *77*, 430–433, doi:10.1111/j.1399-0004.2009.01369.x.

200. Pepe, G.; Chimienti, G.; Resta, F.; Diperna, V.; Tarricone, C.; Lovecchio, M.; Colacicco, A.M.; Capurso, A. A New Italian Case of Lipoprotein Lipase Deficiency: A Leu365→Val Change Resulting in Loss of Enzyme Activity. *Biochemical and Biophysical Research Communications* **1994**, *199*, 570–576, doi:10.1006/bbrc.1994.1266.

201. Zhang, Q.; Chang, G.; Tang, Y.; Gu, S.; Ding, Y.; Chen, Y.; Wang, Y.; Liu, S.; Wang, J.; Wang, X. Genotypic and Phenotypic Features of Dyslipidemia in a Sample of Pediatric Patients in China. *BMC Pediatr* **2023**, *23*, 138, doi:10.1186/s12887-023-03952-z.

202. Minamizuka, T.; Kobayashi, J.; Tada, H.; Miyashita, K.; Koshizaka, M.; Maezawa, Y.; Ono, H.; Yokote, K. Detailed Analysis of Lipolytic Enzymes in a Japanese Woman of Familial Lipoprotein Lipase Deficiency – Effects of Pemafibrate Treatment. *Clinica Chimica Acta* **2020**, *510*, 216–219, doi:10.1016/j.cca.2020.07.031.

203. Hooper, A.J.; Crawford, G.M.; Brisbane, J.M.; Robertson, K.; Watts, G.F.; van Bockxmeer, F.M.; Burnett, J.R. Familial Lipoprotein Lipase Deficiency Caused by Known (G188E) and Novel (W394X) LPL Gene Mutations. *Ann Clin Biochem* **2008**, *45*, 102–105, doi:10.1258/acb.2007.007080.

204. Wiebusch, H.; Funke, H.; Bruin, T.; Bucher, H.; von Eckardstein, A.; Kastelein, J.J.P.; Assmann, G. Compound Heterozygosity for a Known (D250N) and a Novel (E410K) Missense Mutation in the C-Terminal Domain of Lipoprotein Lipase Causes Familial Chylomicronemia. *Human Mutation* **1996**, *8*, 381–383, doi:10.1002/(SICI)1098-1004(1996)8:4<381::AID-HUMU16>3.0.CO;2-Z.

205. Previato, L.; Guardamagna, O.; Dugi, K.A.; Ronan, R.; Talley, G.D.; Santamarina-Fojo, S.; Brewer, H.B. A Novel Missense Mutation in the C-Terminal Domain of Lipoprotein Lipase (Glu410–>Val) Leads to Enzyme Inactivation and Familial Chylomicronemia. *Journal of Lipid Research* **1994**, *35*, 1552–1560, doi:10.1016/S0022-2275(20)41153-8.

206. Gin, P.; Goulbourne, C.N.; Adeyo, O.; Beigneux, A.P.; Davies, B.S.J.; Tat, S.; Voss, C.V.; Bensadoun, A.; Fong, L.G.; Young, S.G. Chylomicronemia Mutations Yield New Insights into Interactions between Lipoprotein Lipase and GPIHBP1. *Human Molecular Genetics* **2012**, *21*, 2961–2972, doi:10.1093/hmg/dds127.

207. Nierman, M.C.; Peter, J.; Khoo, K.-L.; Defesche, J.C. Lipoprotein Lipase Gene Analyses in One Turkish Family and Three Different Chinese Families with Severe Hypertriglyceridaemia: One Novel and Several Established Mutations. *Journal of Inherited Metabolic Disease* **2006**, *29*, 686–686, doi:10.1007/s10545-006-0310-3.

208. Benlian, P.; Etienne, J.; De Gennes, J.L.; Noé, L.; Brault, D.; Raisonnier, A.; Arnault, F.; Hamelin, J.; Foubert, L.; Chuat, J.C. Homozygous Deletion of Exon 9 Causes Lipoprotein Lipase Deficiency: Possible Intron-Alu Recombination. *Journal of Lipid Research* **1995**, *36*, 356–366, doi:10.1016/S0022-2275(20)39913-2.

209. Henderson, H.E.; Hassan, F.; Marais, D.; Hayden, M.R. A New Mutation Destroying Disulphide Bridging in the C-Terminal Domain of Lipoprotein Lipase. *Biochemical and Biophysical Research Communications* **1996**, *227*, 189–194, doi:10.1006/bbrc.1996.1487.

210. Voss, C.V.; Davies, B.S.J.; Tat, S.; Gin, P.; Fong, L.G.; Pelletier, C.; Mottler, C.D.; Bensadoun, A.; Beigneux, A.P.; Young, S.G. Mutations in Lipoprotein Lipase That Block Binding to the Endothelial Cell Transporter GPIHBP1. *Proceedings of the National Academy of Sciences* **2011**, *108*, 7980–7984, doi:10.1073/pnas.1100992108.

211. Henderson, H.; Leisegang, F.; Hassan, F.; Hayden, M.; Marais, D. A Novel Glu421Lys Substitution in the Lipoprotein Lipase Gene in Pregnancy-Induced Hypertriglyceridemic Pancreatitis. *Clinica Chimica Acta* **1998**, *269*, 1–12, doi:10.1016/S0009-8981(97)00144-7.

212. Abedi, A.H.; Yıldırım Şimşir, I.; Bayram, F.; Onay, H.; Özgür, S.; Mcintyre, A.; Toth, P.; Hegele, R. Genetic Variants Associated with Severe Hypertriglyceridemia: LPL, APOC2, APOA5, GPIHBP1, LMF1, and APOE. *Archives of the Turkish Society of Cardiology* **2023**, *51*, 10–21, doi:10.5543/tkda.2022.98544.

213. Yang, W.S.; Nevin, D.N.; Iwasaki, L.; Peng, R.; Brown, B.G.; Brunzell, J.D.; Deeb, S.S. Regulatory Mutations in the Human Lipoprotein Lipase Gene in Patients with Familial Combined Hyperlipidemia and Coronary Artery Disease. *Journal of Lipid Research* **1996**, *37*, 2627–2637, doi:10.1016/S0022-2275(20)37466-6.

214. Geller, A.S.; Polisecki, E.Y.; Diffenderfer, M.R.; Asztalos, B.F.; Karathanasis, S.K.; Hegele, R.A.; Schaefer, E.J. Genetic and Secondary Causes of Severe HDL Deficiency and Cardiovascular Disease1. *J Lipid Res* **2018**, *59*, 2421–2435, doi:10.1194/jlr.M088203.

215. Gagné, E.; Genest, J.; Zhang, H.; Clarke, L.A.; Hayden, M.R. Analysis of DNA Changes in the LPL Gene in Patients with Familial Combined Hyperlipidemia. *Arteriosclerosis and Thrombosis: A Journal of Vascular Biology* **1994**, *14*, 1250–1257, doi:10.1161/01.ATV.14.8.1250.

216. Koopal, C.; Bemelmans, R.; Marais, A.D.; Visseren, F.L. Severe Hypertriglyceridaemia and Pancreatitis in a Patient with Lipoprotein Lipase Deficiency Based on Mutations in Lipoprotein Lipase (LPL) and Apolipoprotein A5 (APOA5) Genes. *BMJ Case Rep* **2019**, *12*, e228199, doi:10.1136/bcr-2018-228199.

217. Henneman, P.; Schaap, F.G.; Rensen, P.C.N.; van Dijk, K.W.; Smelt, A.H.M. Estrogen Induced Hypertriglyceridemia in an Apolipoprotein AV Deficient Patient. *J Intern Med* **2008**, *263*, 107–108, doi:10.1111/j.1365-2796.2007.01889.x.

218. Perera, S.D.; Wang, J.; McIntyre, A.D.; Hegele, R.A. Variability of Longitudinal Triglyceride Phenotype in Patients Heterozygous for Pathogenic *APOA5* Variants. *Journal of Clinical Lipidology* **2023**, *17*, 659–665, doi:10.1016/j.jacl.2023.08.003.

219. Montali, A.; Truglio, G.; Martino, F.; Ceci, F.; Ferraguti, G.; Ciociola, E.; Maranghi, M.; Gianfagna, F.; Iacoviello, L.; Strom, R.; et al. Atherogenic Dyslipidemia in Children: Evaluation of Clinical, Biochemical and Genetic Aspects. *PLoS One* **2015**, *10*, e0120099, doi:10.1371/journal.pone.0120099.

220. Merkel, M.; Eckel, R.H.; Goldberg, I.J. Lipoprotein Lipase: Genetics, Lipid Uptake, and Regulation. *J Lipid Res* **2002**, *43*, 1997–2006, doi:10.1194/jlr.r200015-jlr200.

221. Taghizadeh, E.; Ghayour-Mobarhan, M.; Ferns, G.A.; Pasdar, A. A Novel Variant in LPL Gene Is Associated with Familial Combined Hyperlipidemia. *BioFactors* **2020**, *46*, 94–99, doi:10.1002/biof.1570.

222. Su, Z.G.; Zhang, S.Z.; Hou, Y.P.; Li, T.; Nebert, D.W.; Zhang, L.; Huang, D.J.; Liao, L.C.; Xiao, C.Y. Single-Nucleotide Polymorphisms in the Lipoprotein Lipase Gene Associated with Coronary Heart Disease in Chinese. *European Journal of Pharmacology* **2002**, *454*, 9–18, doi:10.1016/S0014-2999(02)02427-5.

223. Kaplanis, J.; Samocha, K.E.; Wiel, L.; Zhang, Z.; Arvai, K.J.; Eberhardt, R.Y.; Gallone, G.; Lelieveld, S.H.; Martin, H.C.; McRae, J.F.; et al. Evidence for 28 Genetic Disorders Discovered by Combining Healthcare and Research Data. *Nature* **2020**, *586*, 757–762, doi:10.1038/s41586-020-2832-5.

224. Zhou, X.; Feliciano, P.; Shu, C.; Wang, T.; Astrovskaya, I.; Hall, J.B.; Obiajulu, J.U.; Wright, J.R.; Murali, S.C.; Xu, S.X.; et al. Integrating de Novo and Inherited Variants in 42,607 Autism Cases Identifies Mutations in New Moderate-Risk Genes. *Nat Genet* **2022**, *54*, 1305–1319, doi:10.1038/s41588-022-01148-2.

225. Li, J.; Kobori, K.; Kondo, A.; Yonekawa, O.; Kanno, T. [The application of end user computing (EUC) for detection of lipoprotein lipase gene abnormality]. *Rinsho Byori* **1999**, *47*, 737–743.

226. Kassner, U.; Salewsky, B.; Wühle-Demuth, M.; Szijarto, I.A.; Grenkowitz, T.; Binner, P.; März, W.; Steinhagen-Thiessen, E.; Demuth, I. Severe Hypertriglyceridemia in a Patient Heterozygous for a Lipoprotein Lipase Gene Allele with Two Novel Missense Variants. *Eur J Hum Genet* **2015**, *23*, 1259–1261, doi:10.1038/ejhg.2014.295.

227. Pan-Lizcano, R.; Mariñas-Pardo, L.; Núñez, L.; Rebollal-Leal, F.; López-Vázquez, D.; Pereira, A.; Molina-Nieto, A.; Calviño, R.; Vázquez-Rodríguez, J.M.; Hermida-Prieto, M. Rare Variants in Genes of the Cholesterol Pathway Are Present in 60% of Patients with Acute Myocardial Infarction. *International Journal of Molecular Sciences* **2022**, *23*, 16127, doi:10.3390/ijms232416127.

228. Schmella, M.J.; Ferrell, R.E.; Gallaher, M.J.; Lykins, D.L.; Althouse, A.D.; Roberts, J.M.; Hubel, C.A. The -93T/G LPL Promoter Polymorphism Is Associated With Lower Third-Trimester Triglycerides in Pregnant African American Women. *Biol Res Nurs* **2015**, *17*, 429–437, doi:10.1177/1099800414561475.

229. Kastelein, J.J.; Groenemeyer, B.E.; Hallman, D.M.; Henderson, H.; Reymer, P.; Gagné, S.E.; Jansen, H.; Seidell, J.C.; Kromhouf, D.; Jukema, J.W.; et al. The Asn9 Variant of Lipoprotein Lipase Is Associated with the — 93G Promoter Mutation and an Increased Risk of Coronary Artery Disease. *Clinical Genetics* **1998**, *53*, 27–33, doi:10.1111/j.1399-0004.1998.tb02577.x.

230. Samuels, M.E.; Forbey, K.C.; Reid, J.E.; Abkevich, V.; Bulka, K.; Wardell, B.R.; Bowen, B.R.; Hopkins, P.N.; Hunt, S.C.; Ballinger, D.G.; et al. Identification of a Common Variant in the Lipoprotein Lipase Gene in a Large Utah Kindred Ascertained for Coronary Heart Disease: The -93G/D9N Variant Predisposes to Low HDL-C/High Triglycerides. *Clin Genet* **2001**, *59*, 88–98, doi:10.1034/j.1399-0004.2001.590205.x.

231. Karczewski, K.J.; Francioli, L.C.; Tiao, G.; Cummings, B.B.; Alföldi, J.; Wang, Q.; Collins, R.L.; Laricchia, K.M.; Ganna, A.; Birnbaum, D.P.; et al. The Mutational Constraint Spectrum Quantified from Variation in 141,456 Humans. *Nature* **2020**, *581*, 434–443, doi:10.1038/s41586-020-2308-7.

232. Mailly, F.; Tugrul, Y.; Reymer, P.W.; Bruin, T.; Seed, M.; Groenemeyer, B.F.; Asplund-Carlson, A.; Vallance, D.; Winder, A.F.; Miller, G.J. A Common Variant in the Gene for Lipoprotein Lipase (Asp9-->Asn). Functional Implications and Prevalence in Normal and Hyperlipidemic Subjects. *Arterioscler Thromb Vasc Biol* **1995**, *15*, 468–478, doi:10.1161/01.atv.15.4.468.

233. Elbein, S.C.; Yeager, C.; Kwong, L.K.; Lingam, A.; Inoue, I.; Lalouel, J.M.; Wilson, D.E. Molecular Screening of the Lipoprotein Lipase Gene in Hypertriglyceridemic Members of Familial Noninsulin-Dependent Diabetes Mellitus Families. *The Journal of Clinical Endocrinology & Metabolism* **1994**, *79*, 1450–1456, doi:10.1210/jcem.79.5.7962342.

234. Bruin, T.W. a. D.; Mailly, F.; Barlingen, H.H.J.J.V.; Fisher, R.; Cabezas, M.C.; Talmud, P.; Dallinga-Thie, G.M.; Humphries, S.E. Lipoprotein Lipase Gene Mutations D9N and N291S in Four Pedigrees with Familial Combined Hyperlipidaemia. *European Journal of Clinical Investigation* **1996**, *26*, 631–639, doi:10.1111/j.1365-2362.1996.tb02146.x.

235. Hoffer, M.J.V.; Bredie, S.J.H.; Snieder, H.; Reymer, P.W.A.; Demacker, P.N.M.; Havekes, L.M.; Boomsma, D.I.; Stalenhoef, A.F.H.; Frants, R.R.; Kastelein, J.J.P. Gender-Related Association between the −93T→G/D9N Haplotype of the Lipoprotein Lipase Gene and Elevated Lipid Levels in Familial Combined Hyperlipidemia. *Atherosclerosis* **1998**, *138*, 91–99, doi:10.1016/S0021-9150(98)00007-0.

236. Matern, D.; Seydewitz, H.; Niederhoff, H.; Wiebusch, H.; Brandis, M. Dyslipidaemia in a Boy with Recurrent Abdominal Pain, Hypersalivation and Decreased Lipoprotein Lipase Activity. *Eur J Pediatr* **1996**, *155*, 660–664, doi:10.1007/BF01957148.

237. Fisher, R.M.; Benhizia, F.; Schreiber, R.; Makoveichuk, E.; Putt, W.; Al-Haideri, M.; Deckelbaum, R.J.; Olivecrona, G.; Humphries, S.E.; Talmud, P.J. Enhanced Bridging Function and Augmented Monocyte Adhesion by Lipoprotein Lipase N9: Insights into Increased Risk of Coronary Artery Disease in N9 Carriers. *Atherosclerosis* **2003**, *166*, 243–251, doi:10.1016/S0021-9150(02)00337-4.

238. Karagianni, C.; Stabouli, S.; Roumeliotou, K.; Traeger-Synodinos, J.; Kavazarakis, E.; Gourgiotis, D.; Lambrou, J.; Kanavakis, E. Severe Hypertriglyceridaemia in Diabetic Ketoacidosis: Clinical and Genetic Study. *Diabetic Medicine* **2004**, *21*, 380–382, doi:10.1111/j.1464-5491.2004.1111.x.

239. Izar, M.C.; Helfenstein, T.; Ihara, S.S.; Relvas, W.G.; Santos, A.O.; Fischer, S.C.; Pinto, L.E.; Lopes, I.E.; Pomaro, D.R.; Fonseca, M.I.; et al. Association of Lipoprotein Lipase D9N Polymorphism with Myocardial Infarction in Type 2 Diabetes: The

Genetics, Outcomes, and Lipids in Type 2 Diabetes (GOLD) Study. *Atherosclerosis* **2009**, *204*, 165–170, doi:10.1016/j.atherosclerosis.2008.08.006.

240. Corsetti, J.P.; Gansevoort, R.T.; Navis, G.; Sparks, C.E.; Dullaart, R.P.F. LPL Polymorphism (D9N) Predicts Cardiovascular Disease Risk Directly and through Interaction with CETP Polymorphism (TaqIB) in Women with High HDL Cholesterol and CRP. *Atherosclerosis* **2011**, *214*, 373–376, doi:10.1016/j.atherosclerosis.2010.11.029.

241. Rodriguez-Flores, J.L.; Fakhro, K.; Hackett, N.R.; Salit, J.; Fuller, J.; Agosto-Perez, F.; Gharbiah, M.; Malek, J.A.; Zirie, M.; Jayyousi, A.; et al. Exome Sequencing Identifies Potential Risk Variants for Mendelian Disorders at High Prevalence in Qatar. *Hum Mutat* **2014**, *35*, 105–116, doi:10.1002/humu.22460.

242. Dewey, F.E.; Murray, M.F.; Overton, J.D.; Habegger, L.; Leader, J.B.; Fetterolf, S.N.; O'Dushlaine, C.; Van Hout, C.V.; Staples, J.; Gonzaga-Jauregui, C.; et al. Distribution and Clinical Impact of Functional Variants in 50,726 Whole-Exome Sequences from the DiscovEHR Study. *Science* **2016**, *354*, aaf6814, doi:10.1126/science.aaf6814.

243. Myocardial Infarction Genetics and CARDIoGRAM Exome Consortia Investigators; Stitziel, N.O.; Stirrups, K.E.; Masca, N.G.D.; Erdmann, J.; Ferrario, P.G.; König, I.R.; Weeke, P.E.; Webb, T.R.; Auer, P.L.; et al. Coding Variation in ANGPTL4, LPL, and SVEP1 and the Risk of Coronary Disease. *N Engl J Med* **2016**, *374*, 1134–1144, doi:10.1056/NEJMoa1507652.

244. Rutkowska, L.; Sałacińska, K.; Salachna, D.; Matusik, P.; Pinkier, I.; Kępczyński, Ł.; Piotrowicz, M.; Starostecka, E.; Lewiński, A.; Gach, A. Identification of New Genetic Determinants in Pediatric Patients with Familial Hypercholesterolemia Using a Custom NGS Panel. *Genes (Basel)* **2022**, *13*, 999, doi:10.3390/genes13060999.

245. Selvaraj, M.S.; Li, X.; Li, Z.; Pampana, A.; Zhang, D.Y.; Park, J.; Aslibekyan, S.; Bis, J.C.; Brody, J.A.; Cade, B.E.; et al. Whole Genome Sequence Analysis of Blood Lipid Levels in >66,000 Individuals. *Nat Commun* **2022**, *13*, 5995, doi:10.1038/s41467-022-33510-7.

246. Vasiluev, P.A.; Ivanova, O.N.; Semenova, N.A.; Strokova, T.V.; Taran, N.N.; Chubykina, U.V.; Ezhov, M.V.; Zakharova, E.Y.; Dadli, E.L.; Kutsev, S.I. A Clinical Case of a Homozygous Deletion in the APOA5 Gene with Severe Hypertriglyceridemia. *Genes (Basel)* **2022**, *13*, 1062, doi:10.3390/genes13061062.

247. Minicocci, I.; Prisco, C.; Montali, A.; Di Costanzo, A.; Ceci, F.; Pigna, G.; Arca, M. Contribution of Mutations in Low Density Lipoprotein Receptor (*LDLR*) and Lipoprotein Lipase (*LPL*) Genes to Familial Combined Hyperlipidemia (FCHL): A Reappraisal by Using a Resequencing Approach. *Atherosclerosis* **2015**, *242*, 618–624, doi:10.1016/j.atherosclerosis.2015.06.036.

248. Reymer, P.W.A.; Gagné, E.; Groenemeyer, B.E.; Zhang, H.; Forsyth, I.; Jansen, H.; Seidell, J.C.; Kromhout, D.; Lie, K.E.; Kastelein, J.; et al. A Lipoprotein Lipase Mutation (Asn291Ser) Is Associated with Reduced HDL Cholesterol Levels in Premature Atherosclerosis. *Nat Genet* **1995**, *10*, 28–34, doi:10.1038/ng0595-28.

249. Buscà, R.; Peinado, J.; Vilella, E.; Auwerx, J.; Deeb, S.S.; Vilaró, S.; Reina, M. The Mutant Asn291 → Ser Human Lipoprotein Lipase Is Associated with Reduced Catalytic Activity and Does Not Influence Binding to Heparin. *FEBS Letters* **1995**, *367*, 257–262, doi:10.1016/0014-5793(95)00582-T.

250. Syvänne, M.; Antikainen, M.; Ehnholm, S.; Tenkanen, H.; Lahdenperä, S.; Ehnholm, C.; Taskinen, M.R. Heterozygosity for Asn291–>Ser Mutation in the Lipoprotein Lipase Gene in Two Finnish Pedigrees: Effect of Hyperinsulinemia on the Expression of Hypertriglyceridemia. *Journal of Lipid Research* **1996**, *37*, 727–738, doi:10.1016/S0022-2275(20)37571-4.

251. Hoffer, M.J.V.; Bredie, S.J.H.; Boomsma, D.I.; Reymer, P.W.A.; Kastelein, J.J.P.; de Knijff, P.; Demacker, P.N.M.; Stalenhoef, A.F.H.; Havekes, L.M.; Frants, R.R. The Lipoprotein Lipase (Asn291 → Ser) Mutation Is Associated with Elevated Lipid Levels in Families with Familial Combined Hyperlipidaemia. *Atherosclerosis* **1996**, *119*, 159–167, doi:10.1016/0021-9150(95)05641-6.

252. López-Ruiz, A.; Jarabo, M.M.; Martínez-Triguero, M.L.; Morales-Suárez-Varela, M.; Solá, E.; Bañuls, C.; Casado, M.; Hernández-Mijares, A. Small and Dense LDL in Familial Combined Hyperlipidemia and N291S Polymorphism of the Lipoprotein Lipase Gene. *Lipids Health Dis* **2009**, *8*, 12, doi:10.1186/1476-511X-8-12.

253. Smith, A.J.P.; Palmen, J.; Putt, W.; Talmud, P.J.; Humphries, S.E.; Drenos, F. Application of Statistical and Functional Methodologies for the Investigation of Genetic Determinants of Coronary Heart Disease Biomarkers: Lipoprotein Lipase Genotype and Plasma Triglycerides as an Exemplar. *Human Molecular Genetics* **2010**, *19*, 3936–3947, doi:10.1093/hmg/ddq308.

254. Fung, M.; Hill, J.; Cook, D.; Frohlich, J. Case Series of Type III Hyperlipoproteinemia in Children. *Case Reports* **2011**, *2011*, bcr0220113895, doi:10.1136/bcr.02.2011.3895.

255. Peloso, G.M.; Auer, P.L.; Bis, J.C.; Voorman, A.; Morrison, A.C.; Stitziel, N.O.; Brody, J.A.; Khetarpal, S.A.; Crosby, J.R.; Fornage, M.; et al. Association of Low-Frequency and Rare Coding-Sequence Variants with Blood Lipids and Coronary Heart Disease in 56,000 Whites and Blacks. *The American Journal of Human Genetics* **2014**, *94*, 223–232, doi:10.1016/j.ajhg.2014.01.009.

256. Ren, L.; Ren, X. Meta-Analyses of Four Polymorphisms of Lipoprotein Lipase Associated with the Risk of Alzheimer's Disease. *Neuroscience Letters* **2016**, *619*, 73–78, doi:10.1016/j.neulet.2016.03.021.

257. Berg, S.M.; Havelund, J.; Hasler-Sheetal, H.; Kruse, V.; Pedersen, A.J.T.; Hansen, A.B.; Nybo, M.; Beck-Nielsen, H.; Højlund, K.; Færgeman, N.J. The Heterozygous N291S Mutation in the Lipoprotein Lipase Gene Impairs Whole-Body Insulin Sensitivity and Affects a Distinct Set of Plasma Metabolites in Humans. *Journal of Clinical Lipidology* **2017**, *11*, 515-523.e6, doi:10.1016/j.jacl.2017.02.009.

258. Reuter, M.S.; Walker, S.; Thiruvahindrapuram, B.; Whitney, J.; Cohn, I.; Sondheimer, N.; Yuen, R.K.C.; Trost, B.; Paton, T.A.; Pereira, S.L.; et al. The Personal Genome Project Canada: Findings from Whole Genome Sequences of the Inaugural 56 Participants. *CMAJ* **2018**, *190*, E126–E136, doi:10.1503/cmaj.171151.

259. Härtl, J.; Hartberger, J.; Wunderlich, S.; Cordts, I.; Bafligil, C.; Sturm, M.; Westphal, D.; Haack, T.; Hemmer, B.; Ikenberg, B.D.; et al. Exome-Based Gene Panel Analysis in a Cohort of Acute Juvenile Ischemic Stroke Patients:Relevance of NOTCH3 and GLA Variants. *J Neurol* **2023**, *270*, 1501–1511, doi:10.1007/s00415-022-11401-7.

260. Perfilyeva, A.; Bespalova, K.; Perfilyeva, Y.; Skvortsova, L.; Musralina, L.; Zhunussova, G.; Khussainova, E.; Iskakova, U.; Bekmanov, B.; Djansugurova, L. Integrative Functional Genomic Analysis in Multiplex Autism Families from Kazakhstan. *Disease Markers* **2022**, *2022*, 1509994, doi:10.1155/2022/1509994.

261. Nocoń-Bohusz, J.; Wikiera, B.; Basiak, A.; Śmigiel, R.; Noczyńska, A. LPL Gene Mutation as the Cause of Severe Hypertriglyceridemia in the Course of Ketoacidosis in a Patient with Newly Diagnosed Type 1 Diabetes Mellitus. *Pediatr Endocrinol Diabetes Metab* **2016**, *21*, 89–92, doi:10.18544/PEDM-21.02.0029.

262. Chen, Q.; Razzaghi, H.; Demirci, F.Y.; Kamboh, M.I. Functional Significance of Lipoprotein Lipase HindIII Polymorphism Associated with the Risk of Coronary Artery Disease. *Atherosclerosis* **2008**, *200*, 102–108, doi:10.1016/j.atherosclerosis.2007.12.011.

263. Ahn, Y.I.; Kamboh, M.I.; Hamman, R.F.; Cole, S.A.; Ferrell, R.E. Two DNA Polymorphisms in the Lipoprotein Lipase Gene and Their Associations with Factors Related to Cardiovascular Disease. *J Lipid Res* **1993**, *34*, 421–428.

264. Guo, S.; Yang, Z.; Guo, H.; Zhang, J.; Tang, J.; Rui, D.; Ma, R. [Association of lipoprotein lipase gene Hind III and S447X polymorphisms in metabolic syndrome patients among Kazakh and Han ethnics from Xinjiang]. *Zhonghua Liu Xing Bing Xue Za Zhi* **2010**, *31*, 992–996.

265. Smith, A.J.P.; Drenos, F.; Palmen, J.; Putt, W.; Talmud, P.J.; Humphries, S.E. FUNCTIONAL ANALYSIS OF REGULATORY LPL SNPS. *Atherosclerosis* **2009**, *207*, e3–e4, doi:10.1016/j.atherosclerosis.2009.09.037.

266. Ranganathan, G.; Unal, R.; Pokrovskaya, I.D.; Tripathi, P.; Rotter, J.I.; Goodarzi, M.O.; Kern, P.A. The Lipoprotein Lipase (LPL) S447X Gain of Function Variant Involves Increased mRNA Translation. *Atherosclerosis* **2011**, *221*, 143, doi:10.1016/j.atherosclerosis.2011.12.028.

267. Goodarzi, M.O.; Wong, H.; Quiñones, M.J.; Taylor, K.D.; Guo, X.; Castellani, L.W.; Antoine, H.J.; Yang, H.; Hsueh, W.A.; Rotter, J.I. The 3′ Untranslated Region of the Lipoprotein Lipase Gene: Haplotype Structure and Association with Post-Heparin Plasma Lipase Activity. *The Journal of Clinical Endocrinology & Metabolism* **2005**, *90*, 4816–4823, doi:10.1210/jc.2005-0389.

268. Caussy, C.; Charrière, S.; Meirhaeghe, A.; Dallongeville, J.; Lefai, E.; Rome, S.; Cuerq, C.; Euthine, V.; Delay, M.; Marmontel, O.; et al. Multiple microRNA Regulation of Lipoprotein Lipase Gene Abolished by 3'UTR Polymorphisms in a Triglyceride-Lowering Haplotype Harboring p.Ser474Ter. *Atherosclerosis* **2016**, *246*, 280–286, doi:10.1016/j.atherosclerosis.2016.01.010.

269. Hata, A.; Robertson, M.; Emi, M.; Lalouel, J.M. Direct Detection and Automated Sequencing of Individual Alleles after Electrophoretic Strand Separation: Identification of a Common Nonsense Mutation in Exon 9 of the Human Lipoprotein Lipase Gene. *Nucleic Acids Res* **1990**, *18*, 5407–5411, doi:10.1093/nar/18.18.5407.

270. Gagné, S.; Larson, M.; Pimstone, S.; Schaefer, E.; Kastelein, J.; Wilson, P.; Ordovas, J.; Hayden, M. A Common Truncation Variant of Lipoprotein Lipase (Ser447X) Confers Protection against Coronary Heart Disease: The Framingham Offspring Study. *Clinical Genetics* **1999**, *55*, 450–454, doi:10.1034/j.1399-0004.1999.550609.x.

271. Liu, A.; Li, L.; Cao, W.; Shan, S.; Lu, J.; Guo, X.; Hu, Y. [The association of S447X and Hind III polymorphism in the lipoprotein lipase gene with dyslipidemia of the metabolic syndrome in patients with essential hypertension]. *Zhonghua Yi Xue Yi Chuan Xue Za Zhi* **2005**, *22*, 151–157.

272. Guan, G.; Xu, E.; Wang, X.; Xu, Y.; Qiu, S. Associations between Ser447Ter Gene Polymorphism of Lipoprotein Lipase and Atherosclerotic Cerebral Infarction. *Zhonghua yi xue yi chuan xue za zhi = Zhonghua yixue yichuanxue zazhi = Chinese journal of medical genetics* **2006**, *23*, 519–522.

273. Herbeth, B.; Gueguen, S.; Leroy, P.; Siest, G.; Visvikis-Siest, S. The Lipoprotein Lipase Serine 447 Stop Polymorphism Is Associated with Altered Serum Carotenoid Concentrations in the Stanislas Family Study. *J Am Coll Nutr* **2007**, *26*, 655–662, doi:10.1080/07315724.2007.10719644.

274. Komurcu-Bayrak, E.; Onat, A.; Poda, M.; Humphries, S.E.; Acharya, J.; Hergenc, G.; Coban, N.; Can, G.; Erginel-Unaltuna, N. The S447X Variant of Lipoprotein Lipase Gene Is Associated with Metabolic Syndrome and Lipid Levels among Turks. *Clinica Chimica Acta* **2007**, *383*, 110–115, doi:10.1016/j.cca.2007.05.007.

275. Talmud, P.J.; Flavell, D.M.; Alfakih, K.; Cooper, J.A.; Balmforth, A.J.; Sivananthan, M.; Montgomery, H.E.; Hall, A.S.; Humphries, S.E. The Lipoprotein Lipase Gene Serine 447 Stop Variant Influences Hypertension-Induced Left Ventricular Hypertrophy and Risk of Coronary Heart Disease. *Clin Sci (Lond)* **2007**, *112*, 617–624, doi:10.1042/cs20060344.

276. Aydogan, H.Y.; Isbir, S.; Kurnaz, O.; Gormus, U.; Isbir, T. Associations of Lipoprotein Lipase S447X and Apolipoprotein E Genotypes with Low-Density Lipoprotein Subfractions in Turkish Patients with Coronary Artery Disease. *In Vivo* **2009**, *23*, 155–161.

277. Chang, Y.-T.; Chang, M.-C.; Su, T.-C.; Liang, P.-C.; Su, Y.-N.; Kuo, C.-H.; Wei, S.-C.; Wong, J.-M. Lipoprotein Lipase Mutation S447X Associated with Pancreatic Calcification and Steatorrhea in Hyperlipidemic Pancreatitis. *J Clin Gastroenterol* **2009**, *43*, 591–596, doi:10.1097/MCG.0b013e3181734a30.

278. Deo, R.C.; Reich, D.; Tandon, A.; Akylbekova, E.; Patterson, N.; Waliszewska, A.; Kathiresan, S.; Sarpong, D.; Taylor, H.A.; Wilson, J.G. Genetic Differences between the Determinants of Lipid Profile Phenotypes in African and European Americans: The Jackson Heart Study. *PLoS Genet* **2009**, *5*, e1000342, doi:10.1371/journal.pgen.1000342.

279. Fujiwara, S.; Kotani, K.; Sano, Y.; Matsuoka, Y.; Tsuzaki, K.; Domichi, M.; Kajii, E.; Sakane, N. S447X Polymorphism in the Lipoprotein Lipase Gene and the Adiponectin Level in the General Population: Results from the Mima Study. *JAT* **2009**, *16*, 188–193, doi:10.5551/jat.E593.

280. Jensen, M.K.; Rimm, E.B.; Rader, D.; Schmidt, E.B.; Sørensen, T.I.A.; Vogel, U.; Overvad, K.; Mukamal, K.J. S447X Variant of the Lipoprotein Lipase Gene, Lipids, and Risk of Coronary Heart Disease in 3 Prospective Cohort Studies. *Am Heart J* **2009**, *157*, 384–390, doi:10.1016/j.ahj.2008.10.008.

281. Salah, A.; Khan, M.; Esmail, N.; Habibullah, S.; Al Lahham, Y. Genetic Polymorphism of S447X Lipoprotein Lipase (LPL) and the Susceptibility to Hypertension. *J Crit Care* **2009**, *24*, e11-14, doi:10.1016/j.jcrc.2009.06.005.

282. van Hoek, M.; Dallinga-Thie, G.M.; Steyerberg, E.W.; Sijbrands, E.J.G. Diagnostic Value of Post-Heparin Lipase Testing in Detecting Common Genetic Variants in the LPL and LIPC Genes. *Eur J Hum Genet* **2009**, *17*, 1386–1393, doi:10.1038/ejhg.2009.61.

283. Webster, R.J.; Warrington, N.M.; Weedon, M.N.; Hattersley, A.T.; McCaskie, P.A.; Beilby, J.P.; Palmer, L.J.; Frayling, T.M. The Association of Common Genetic Variants in the APOA5, LPL and GCK Genes with Longitudinal Changes in Metabolic and Cardiovascular Traits. *Diabetologia* **2009**, *52*, 106–114, doi:10.1007/s00125-008-1175-9.

284. Wang, C.; Sun, T.; Li, H.; Bai, J.; Li, Y. Lipoprotein Lipase Ser447Ter Polymorphism Associated with the Risk of Ischemic Stroke: A Meta-Analysis. *Thromb Res* **2011**, *128*, e107-112, doi:10.1016/j.thromres.2011.07.016.

285. Takeuchi, F.; Isono, M.; Katsuya, T.; Yokota, M.; Yamamoto, K.; Nabika, T.; Shimokawa, K.; Nakashima, E.; Sugiyama, T.; Rakugi, H.; et al. Association of Genetic Variants Influencing Lipid Levels with Coronary Artery Disease in Japanese Individuals. *PLoS ONE* **2012**, *7*, e46385, doi:10.1371/journal.pone.0046385.

286. Brownstein, C.A.; Towne, M.C.; Luquette, L.J.; Harris, D.J.; Marinakis, N.S.; Meinecke, P.; Kutsche, K.; Campeau, P.M.; Yu, T.W.; Margulies, D.M.; et al. Mutation of *KCNJ8* in a Patient with Cantú Syndrome with Unique Vascular Abnormalities – Support for the Role of K(ATP) Channels in This Condition. *European Journal of Medical Genetics* **2013**, *56*, 678–682, doi:10.1016/j.ejmg.2013.09.009.

287. Velapasamy, S.; Alex, L.; Chahil, J.K.; Lye, S.H.; Munretnam, K.; Nor Hashim, N.A.; Ramzi, N.H.; Mohd Nordin, N.; Visvalingam, V.; Ler, L.W. Influences of Multiple Genetic Polymorphisms on Ovarian Cancer Risk in Malaysia. *Genetic Testing and Molecular Biomarkers* **2013**, *17*, 62–68, doi:10.1089/gtmb.2012.0223.

288. Xu, X.; Wang, Y.; Wang, L.; Liao, Q.; Chang, L.; Xu, L.; Huang, Y.; Ye, H.; Xu, L.; Chen, C.; et al. Meta-Analyses of 8 Polymorphisms Associated with the Risk of the Alzheimer's Disease. *PLoS One* **2013**, *8*, e73129, doi:10.1371/journal.pone.0073129.

289. Bentley, A.R.; Chen, G.; Shriner, D.; Doumatey, A.P.; Zhou, J.; Huang, H.; Mullikin, J.C.; Blakesley, R.W.; Hansen, N.F.; Bouffard, G.G.; et al. Gene-Based Sequencing Identifies Lipid-Influencing Variants with Ethnicity-Specific Effects in African Americans. *PLoS Genet* **2014**, *10*, e1004190, doi:10.1371/journal.pgen.1004190.

290. Turlo, K.; Leung, C.S.; Seo, J.J.; Goulbourne, C.N.; Adeyo, O.; Gin, P.; Voss, C.; Bensadoun, A.; Fong, L.G.; Young, S.G.; et al. Equivalent Binding of Wild-Type Lipoprotein Lipase (LPL) and S447X-LPL to GPIHBP1, the Endothelial Cell LPL Transporter. *Biochimica et biophysica acta* **2014**, *1841*, 963, doi:10.1016/j.bbalip.2014.03.011.

291. Zambrano Morales, M.; Fernández Salgado, E.; Balzán Urdaneta, L.; Labastidas, N.; Aranguren-Méndez, J.; Connell, L.; Molero Paredes, T.; Rojas, A.; Panunzio, A. [Lack of association between the S447X variant of the lipoprotein lipase gene and plasma lipids. A preliminary study]. *Invest Clin* **2014**, *55*, 133–141.

292. Emamian, M.; Avan, A.; Pasdar, A.; Mirhafez, S.R.; Sadeghzadeh, M.; Moghadam, M.S.; Parizadeh, S.M.R.; Ferns, G.A.; Ghayour-Mobarhan, M. The Lipoprotein Lipase S447X and Cholesteryl Ester Transfer Protein Rs5882 Polymorphisms and Their Relationship with Lipid Profile in Human Serum of Obese Individuals. *Gene* **2015**, *558*, 195–199, doi:10.1016/j.gene.2014.12.070.

293. Dewey, F.E.; Murray, M.F.; Overton, J.D.; Habegger, L.; Leader, J.B.; Fetterolf, S.N.; O'Dushlaine, C.; Van Hout, C.V.; Staples, J.; Gonzaga-Jauregui, C.; et al. Distribution and Clinical Impact of Functional Variants in 50,726 Whole-Exome Sequences from the DiscovEHR Study. *Science* **2016**, *354*, aaf6814, doi:10.1126/science.aaf6814.

294. Lu, X.; Li, J.; Li, H.; Chen, Y.; Wang, L.; He, M.; Wang, Y.; Sun, L.; Hu, Y.; Huang, J.; et al. Coding-Sequence Variants Are Associated with Blood Lipid Levels in 14,473 Chinese. *Human Molecular Genetics* **2016**, *25*, 4107–4116, doi:10.1093/hmg/ddw261.

295. Shatwan, I.M.; Minihane, A.-M.; Williams, C.M.; Lovegrove, J.A.; Jackson, K.G.; Vimaleswaran, K.S. Impact of Lipoprotein Lipase Gene Polymorphism, S447X, on Postprandial Triacylglycerol and Glucose Response to Sequential Meal Ingestion. *Int J Mol Sci* **2016**, *17*, 397, doi:10.3390/ijms17030397.

296. Verma, A.; Verma, S.S.; Pendergrass, S.A.; Crawford, D.C.; Crosslin, D.R.; Kuivaniemi, H.; Bush, W.S.; Bradford, Y.; Kullo, I.; Bielinski, S.J.; et al. eMERGE Phenome-Wide Association Study (PheWAS) Identifies Clinical Associations and Pleiotropy for Stop-Gain Variants. *BMC Med Genomics* **2016**, *9 Suppl 1*, 32, doi:10.1186/s12920-016-0191-8.

297. Hayne, C.K.; Lafferty, M.J.; Eglinger, B.J.; Kane, J.P.; Neher, S.B. Biochemical Analysis of the Lipoprotein Lipase Truncation Variant, LPLS447X, Reveals Increased Lipoprotein Uptake. *Biochemistry* **2017**, *56*, 525–533, doi:10.1021/acs.biochem.6b00945.

298. Liu, D.J.; Peloso, G.M.; Yu, H.; Butterworth, A.S.; Wang, X.; Mahajan, A.; Saleheen, D.; Emdin, C.; Alam, D.; Alves, A.C.; et al. Exome-Wide Association Study of Plasma Lipids in >300,000 Individuals. *Nat Genet* **2017**, *49*, 1758–1766, doi:10.1038/ng.3977.

299. Lu, X.; Peloso, G.M.; Liu, D.J.; Wu, Y.; Zhang, H.; Zhou, W.; Li, J.; Tang, C.S.-M.; Dorajoo, R.; Li, H.; et al. Exome Chip Meta-Analysis Identifies Novel Loci and East Asian-Specific Coding Variants That Contribute to Lipid Levels and Coronary Artery Disease. *Nat Genet* **2017**, *49*, 1722–1730, doi:10.1038/ng.3978.

300. Wang, J.; Du, S.; Wang, J.; Zhu, M.; Wen, X.; Yang, W. Association of the Lipoprotein Lipase Gene Ser447Ter Polymorphism with Hypertension and Blood Pressure Variation: Evidence from an Updated Meta-Analysis. *Clinical and Experimental Hypertension* **2017**, *39*, 655–664, doi:10.1080/10641963.2017.1313848.

301. Mahajan, A.; Wessel, J.; Willems, S.M.; Zhao, W.; Robertson, N.R.; Chu, A.Y.; Gan, W.; Kitajima, H.; Taliun, D.; Rayner, N.W.; et al. Refining the Accuracy of Validated Target Identification through Coding Variant Fine-Mapping in Type 2 Diabetes. *Nat Genet* **2018**, *50*, 559–571, doi:10.1038/s41588-018-0084-1.

302. Sun, W.; Wu, Y.; Wen, Y.; Guo, M.; Zhang, H. The Association of the S447X Mutation in LPL with Coronary Artery Disease: A Meta-Analysis. *Minerva Cardioangiol* **2019**, *67*, 246–253, doi:10.23736/S0026-4725.18.04668-6.

303. Nielsen, J.B.; Rom, O.; Surakka, I.; Graham, S.E.; Zhou, W.; Roychowdhury, T.; Fritsche, L.G.; Gagliano Taliun, S.A.; Sidore, C.; Liu, Y.; et al. Loss-of-Function Genomic Variants Highlight Potential Therapeutic Targets for Cardiovascular Disease. *Nat Commun* **2020**, *11*, 6417, doi:10.1038/s41467-020-20086-3.

304. Selvaraj, M.S.; Li, X.; Li, Z.; Pampana, A.; Zhang, D.Y.; Park, J.; Aslibekyan, S.; Bis, J.C.; Brody, J.A.; Cade, B.E.; et al. Whole Genome Sequence Analysis of Blood Lipid Levels in >66,000 Individuals. *Nat Commun* **2022**, *13*, 5995, doi:10.1038/s41467-022-33510-7.

305. Yang, L.-X.; Razzaghi, H.; Hokanson, J.E.; Kamboh, M.I. Identification and Characterization of a Novel 5 Bp Deletion in a Putative Insulin Response Element in the Lipoprotein Lipase Gene. *Biochim Biophys Acta* **2009**, *1791*, 1057–1065, doi:10.1016/j.bbalip.2009.06.003.

306. Ghanbari, M.; Franco, O.H.; de Looper, H.W.J.; Hofman, A.; Erkeland, S.J.; Dehghan, A. Genetic Variations in MicroRNA-Binding Sites Affect MicroRNA-Mediated Regulation of Several Genes Associated With Cardio-Metabolic Phenotypes. *Circulation: Cardiovascular Genetics* **2015**, *8*, 473–486, doi:10.1161/CIRCGENETICS.114.000968.

307. Richardson, K.; Nettleton, J.A.; Rotllan, N.; Tanaka, T.; Smith, C.E.; Lai, C.-Q.; Parnell, L.D.; Lee, Y.-C.; Lahti, J.; Lemaitre, R.N.; et al. Gain-of-Function Lipoprotein Lipase Variant Rs13702 Modulates Lipid Traits through Disruption of a microRNA-410 Seed Site. *Am J Hum Genet* **2013**, *92*, 5–14, doi:10.1016/j.ajhg.2012.10.020.

308. Corella, D.; Sorlí, J.V.; Estruch, R.; Coltell, O.; Ortega-Azorín, C.; Portolés, O.; Martínez-González, M.Á.; Bulló, M.; Fitó, M.; Arós, F.; et al. MicroRNA-410 Regulated Lipoprotein Lipase Variant Rs13702 Is Associated with Stroke Incidence and Modulated by Diet in the Randomized Controlled PREDIMED Trial. *Am J Clin Nutr* **2014**, *100*, 719–731, doi:10.3945/ajcn.113.076992.

309. Kraja, A.T.; Vaidya, D.; Pankow, J.S.; Goodarzi, M.O.; Assimes, T.L.; Kullo, I.J.; Sovio, U.; Mathias, R.A.; Sun, Y.V.; Franceschini, N.; et al. A Bivariate Genome-Wide Approach to Metabolic Syndrome: STAMPEED Consortium. *Diabetes* **2011**, *60*, 1329–1339, doi:10.2337/db10-1011.

310. Mo, X.; Liu, X.; Wang, L.; Li, H.; Lu, X.; Huang, J.; Chen, J.; Cao, J.; Li, J.; Chen, S.; et al. Lipoprotein Lipase Gene Polymorphism Rs1059611 Functionally Influences Serum Lipid Concentrations. *Atherosclerosis* **2013**, *229*, 511–516, doi:10.1016/j.atherosclerosis.2013.05.005.
